# Supplementary material for: Synthesis, Cytotoxic Activity and In Silico Study of Novel Dihydropyridine Carboxylic Acids Derivatives
Source: Int J Mol Sci. 2023 Oct 21;24(20):15414. doi: 10.3390/ijms242015414 (PMC10607468; doi:10.3390/ijms242015414)
Supplement: Supplementary file 1 [file ijms-24-15414-s001.zip › ijms-2627441-supplementary.pdf]

Synthesis, Cytotoxic Activity and in Silico Study of Novel Dihydropyridine Carboxylic Acids Derivatives

Ricardo Ballinas-Indili, María Inés Nicolás-Vázquez, Joel Martínez, María Teresa Ramírez-Apan, Cecilio Álvarez-Toledano, Maricarmen Hernández-Rodríguez, Elvia Mera Jiménez, and René Miranda Ruvalcaba.

Content

Figure S1. ^{1}H NMR (300 MHz/$CDCl_3$/TMS) of **3a**.
Figure S2. ^{13}C NMR (75 MHz/$CDCl_3$/TMS) of **3a**.
Figure S3. FTIR of **3a**.
Figure S4. HRMS-DART^{+} (19 eV) of **3a**.
Figure S5. ^{1}H NMR (300 MHz/$CDCl_3$/TMS) of **3b**.
Figure S6. ^{13}C NMR (75 MHz/$CDCl_3$/TMS) of **3b**.
Figure S7. FTIR of **3b**.
Figure S8. HRMS-DART^{+} (19 eV) of **3b**.
Figure S9. ^{1}H NMR (300 MHz/$CDCl_3$/TMS) of **3c**.
Figure S10. ^{13}C NMR (75 MHz/$CDCl_3$/TMS) of **3c**.
Figure S11. FTIR of **3c**.
Figure S12. HRMS-DART^{+} (19 eV) of **3c**.
Figure S13. ^{1}H NMR (300 MHz/$CDCl_3$/TMS) of **3d**.
Figure S14. ^{13}C NMR (75 MHz/$CDCl_3$/TMS) of **3d**.
Figure S15. FTIR of **3d**.
Figure S16. HRMS-DART^{+} (19 eV) of **3d**.
Figure S17. ^{1}H NMR (300 MHz/$CDCl_3$/TMS) of **3e**.
Figure S18. ^{13}C NMR (75 MHz/$CDCl_3$/TMS) of **3e**.
Figure S19. FTIR of **3e**.
Figure S20. HRMS-DART^{+} (19 eV) of **3e**.
Figure S21. ^{1}H NMR (300 MHz/$CDCl_3$/TMS) of **3f**.
Figure S22. ^{13}C NMR (75 MHz/$CDCl_3$/TMS) of **3f**.
Figure S23. FTIR of **3f**.
Figure S24. HRMS-DART^{+} (19 eV) of **3f**.
Figure S25. ^{1}H NMR (300 MHz/$CDCl_3$/TMS) of **3g**.
Figure S26. ^{13}C NMR (75 MHz/$CDCl_3$/TMS) of **3g**.
Figure S27. FTIR of **3g**.
Figure S28. HRMS-DART^{+} (19 eV) of **3g**.
Figure S29. ^{1}H NMR (300 MHz/$CDCl_3$/TMS) of **3h**.
Figure S30. ^{13}C NMR (75 MHz/$CDCl_3$/TMS) of **3h**.
Figure S31. FTIR of **3h**.
Figure S32. HRMS-DART^{+} (19 eV) of **3h**.
Figure S33. ^{1}H NMR (300 MHz/$CDCl_3$/TMS) of **3i**.
Figure S34. ^{13}C NMR (75 MHz/$CDCl_3$/TMS) of **3i**.
Figure S35. FTIR of **3i**.
Figure S36. HRMS-DART^{+} (19 eV) of **3i**.

Figure S37. ^{1}H NMR (300 MHz/$CDCl_3$/TMS) of **3j**.
Figure S38. ^{13}C NMR (75 MHz/$CDCl_3$/TMS) of **3j**.
Figure S39. FTIR of **3j**.
Figure S40. HRMS-DART^{+} (19 eV) of **3j**.
Figure S41. ^{1}H NMR (300 MHz/$CDCl_3$/TMS) of **3k**.
Figure S42. ^{13}C NMR (75 MHz/$CDCl_3$/TMS) of **3k**.
Figure S43. FTIR of **3k**.
Figure S44. HRMS-DART^{+} (19 eV) of **3k**.
Figure S45. ^{1}H NMR (300 MHz/$CDCl_3$/TMS) of **3l**.
Figure S46. ^{13}C NMR (75 MHz/$CDCl_3$/TMS) of **3l**.
Figure S47. FTIR of **3l**.
Figure S48. HRMS-DART^{+} (19 eV) of **3l**.
Figure S49. ^{1}H NMR (300 MHz/$CDCl_3$/TMS) of **3m**.
Figure S50. ^{13}C NMR (75 MHz/$CDCl_3$/TMS) of **3m**.
Figure S51. FTIR of **3m**.
Figure S52. HRMS-DART^{+} (19 eV) of **3m**.
Figure S53. ^{1}H NMR (300 MHz/$CDCl_3$/TMS) of **3n**.
Figure S54. ^{13}C NMR (75 MHz/$CDCl_3$/TMS) of **3n**.
Figure S55. FTIR of **3n**.
Figure S56. HRMS-DART^{+} (19 eV) of **3n**.
Figure S57. ^{1}H NMR (300 MHz/$CDCl_3$/TMS) of **3o**.
Figure S58. ^{13}C NMR (75 MHz/$CDCl_3$/TMS) of **3o**.
Figure S59. FTIR of **3o**.
Figure S60. HRMS-DART^{+} (19 eV) of **3o**.
Figure S61. ^{1}H NMR (300 MHz/$CDCl_3$/TMS) of **3p**.
Figure S62. ^{13}C NMR (75 MHz/$CDCl_3$/TMS) of **3p**.
Figure S63. FTIR of **3p**.
Figure S64. HRMS-DART^{+} (19 eV) of **3p**.

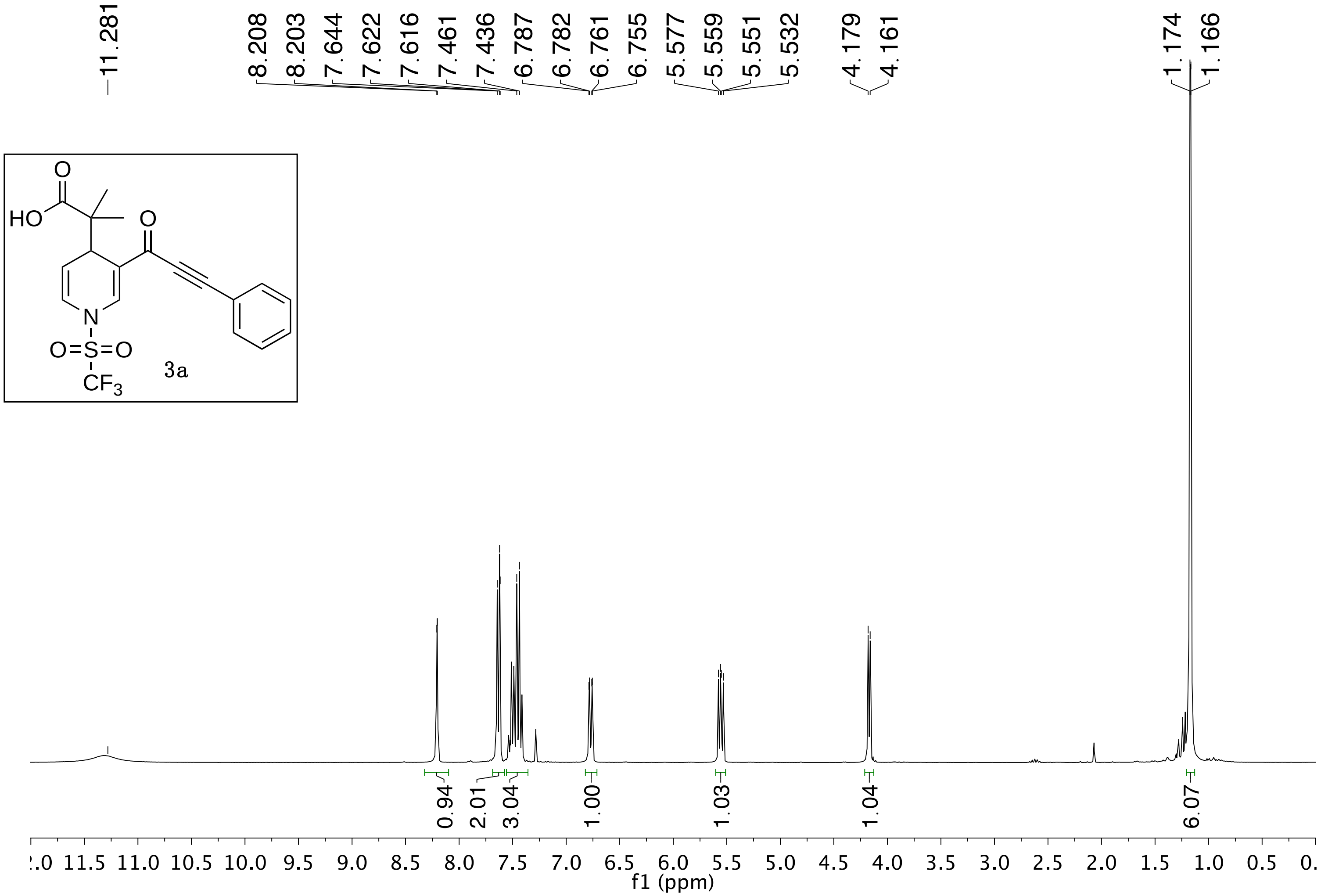

Figure S1. ^{1}H NMR (300 MHz/$CDCl_3$/TMS) of **3a**.

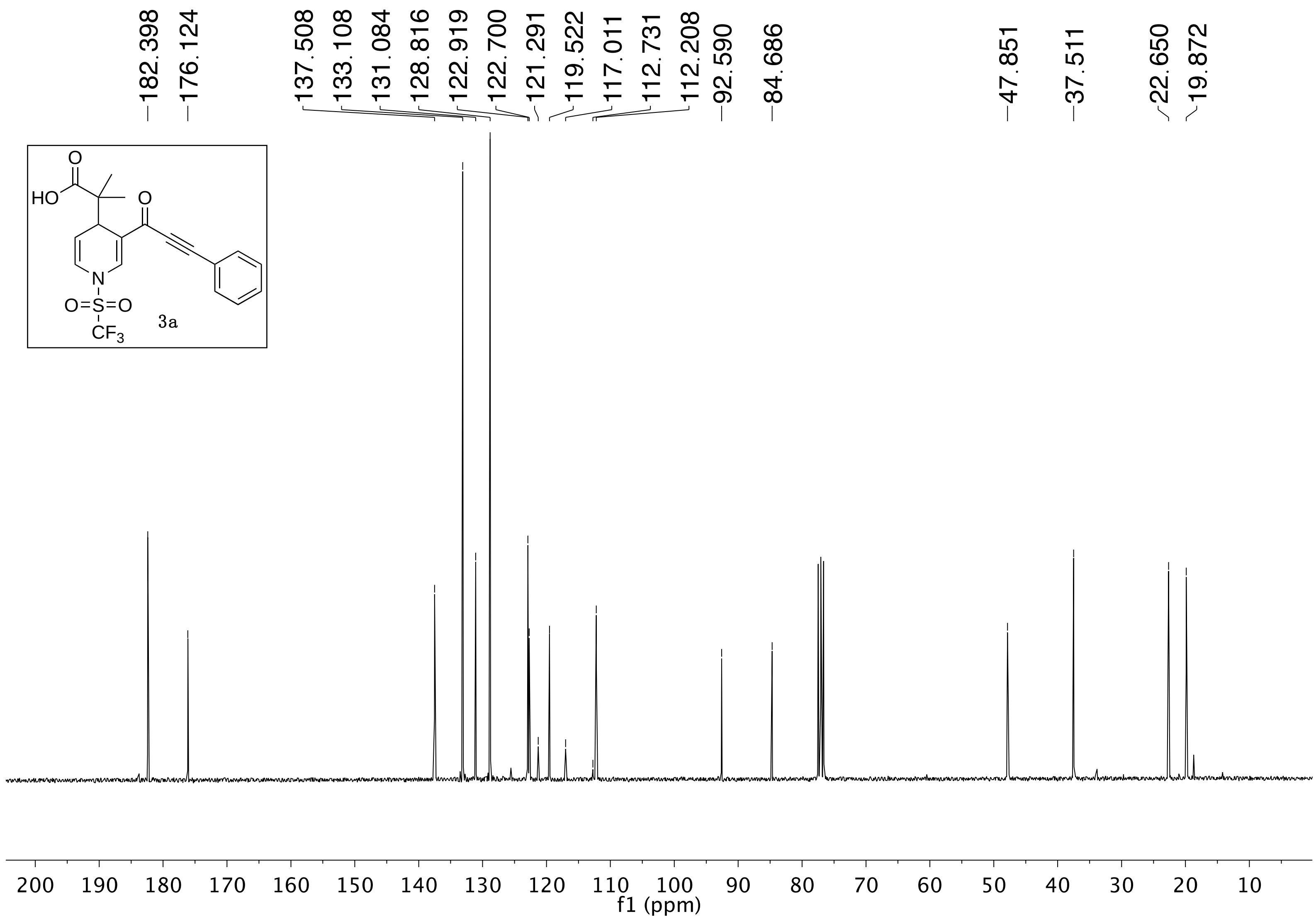

Figure S2. ^{13}C NMR (75 MHz/$CDCl_3$/TMS) of **3a**.

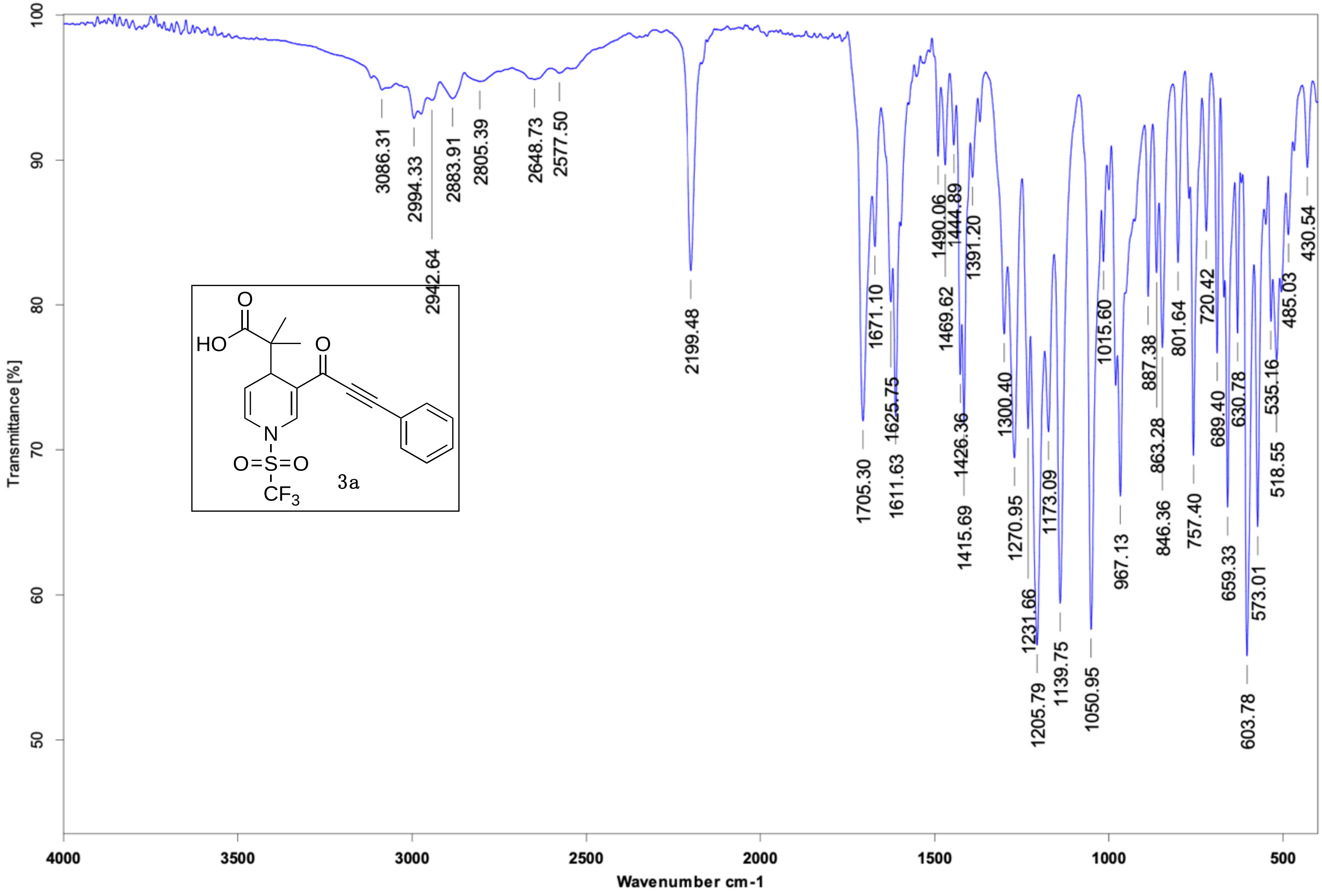

Figure S3. FTIR of **3a**.

Description:
Ionization Mode:ESI+
History:Determine m/z[Peak Detect[Centroid,30,Area];Correct Base[1.0%];Smooth[5]];Correct Base[5.0%];Average...

Mass Calibration data:Cal_Peg_600
Created:11/22/2018 1:46:29 PM
Created by:AccuTOF

Charge number:1
Tolerance:3.00(mmu)
Unsaturation Number:0.0 .. 50.0 (Fraction:Both)
Element:^{12}C:0 .. 19, ^{1}H:0 .. 17, ^{19}F:0 .. 3, ^{14}N:0 .. 1, ^{16}O:0 .. 5, ^{32}S:0 .. 1

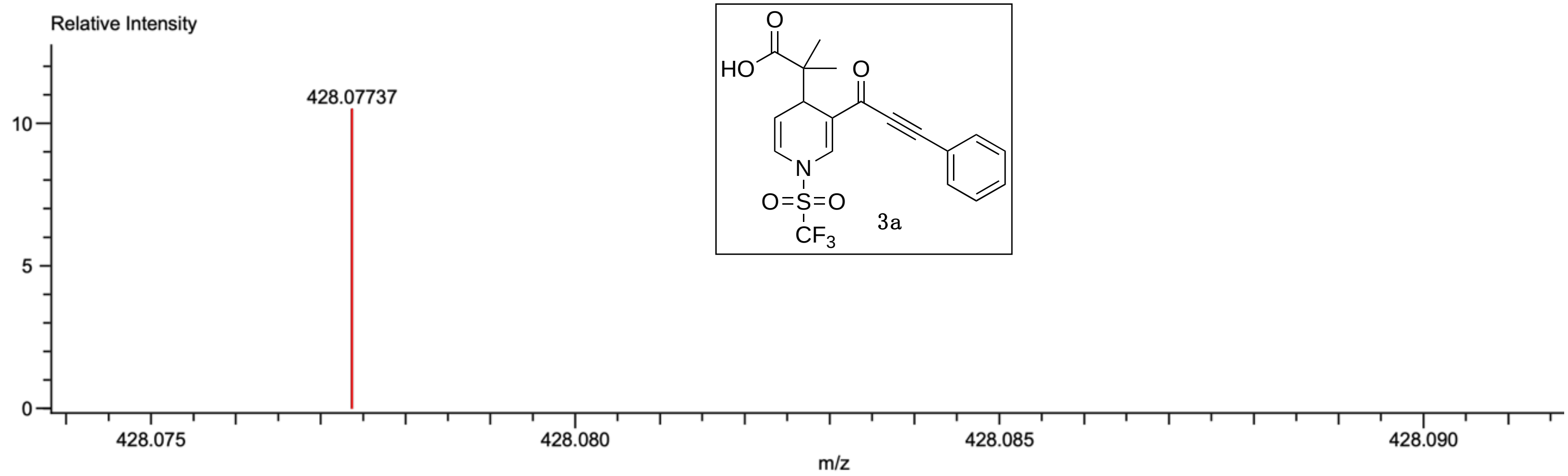

Mass	Intensity	Calc. Mass	Mass Difference (mmu)	Mass Difference (ppm)	Possible Formula	Unsaturation Number
428.07737	303481.46	428.07795	-0.58	-1.37	$^{12}C_{19}{}^{1}H_{17}{}^{19}F_{3}{}^{14}N_{1}{}^{16}O_{5}{}^{32}S_{1}$	11.5

Figure S4. HRMS-DART^{+} (19 eV) of **3a**.

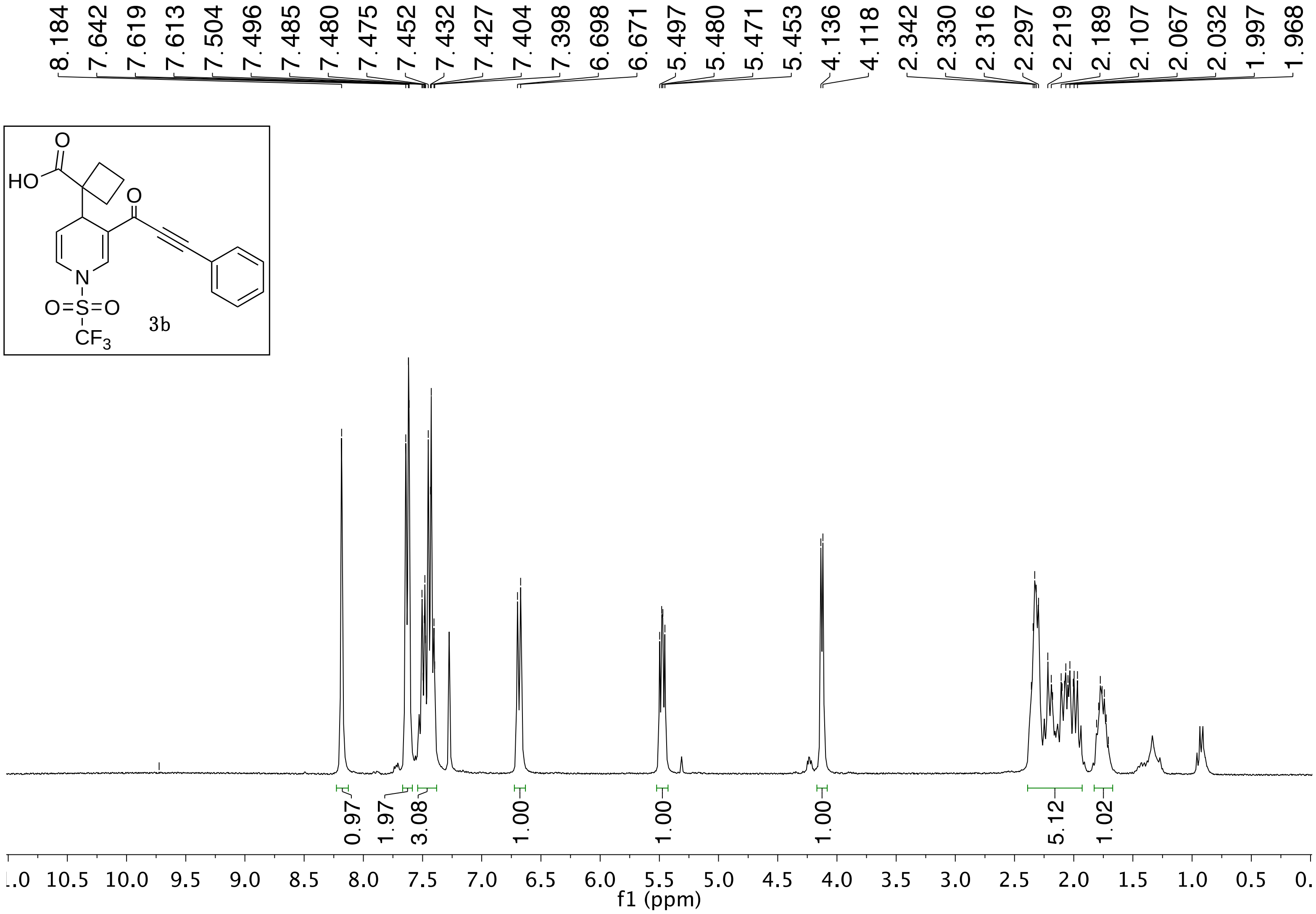

Figure S5. ^{1}H NMR (300 MHz/$CDCl_3$/TMS) of **3b**.

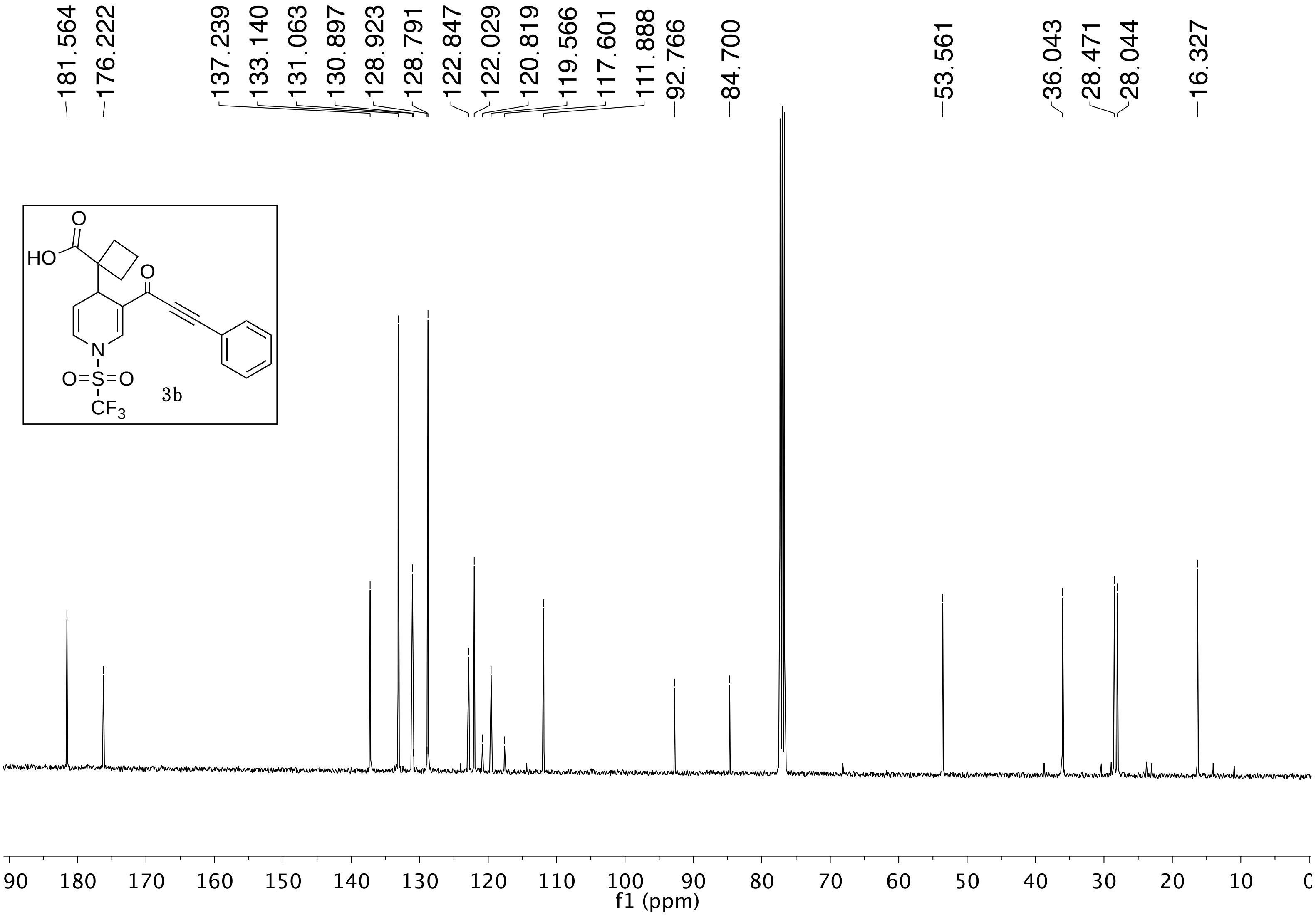

Figure S6. ^{13}C NMR (75 MHz/$CDCl_3$/TMS) of **3b**.

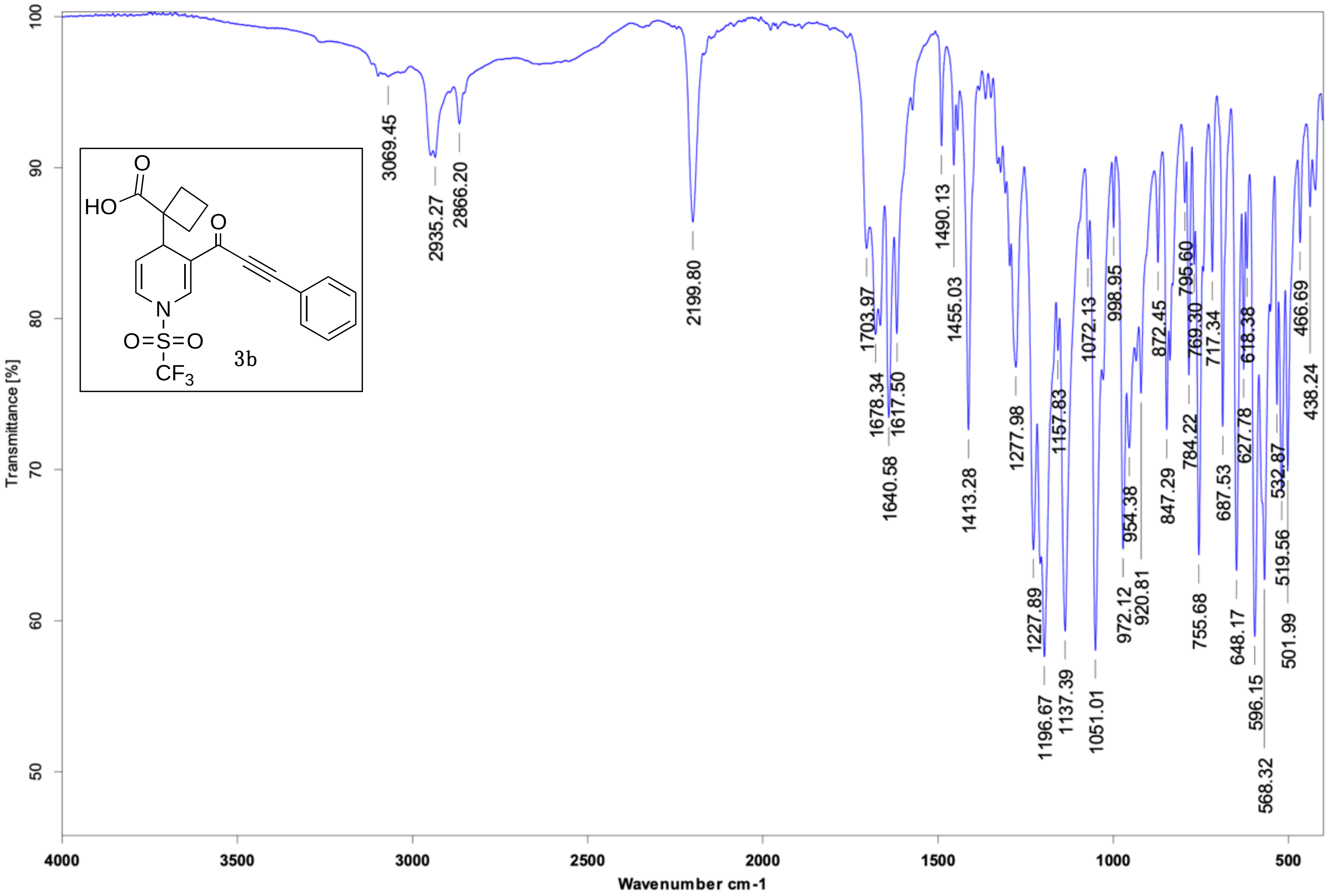

Figure S7. FTIR of **3b**.

Description:
Ionization Mode:ESI+
History:Determine m/z[Peak Detect[Centroid,30,Area];Correct Base[];Smooth[5]];Correct Base[5.0%];Average(MS[...

Mass Calibration data:Cal_PEG_600
Created:10/6/2022 11:38:15 AM
Created by:AccuTOF

Charge number:1
Tolerance:5.00(mmu)
Unsaturation Number:0.0 .. 50.0 (Fraction:Both)
Element:^{12}C:0 .. 20, ^{1}H:0 .. 22, ^{19}F:0 .. 3, ^{14}N:0 .. 1, ^{16}O:1 .. 6, ^{32}S:1 .. 1

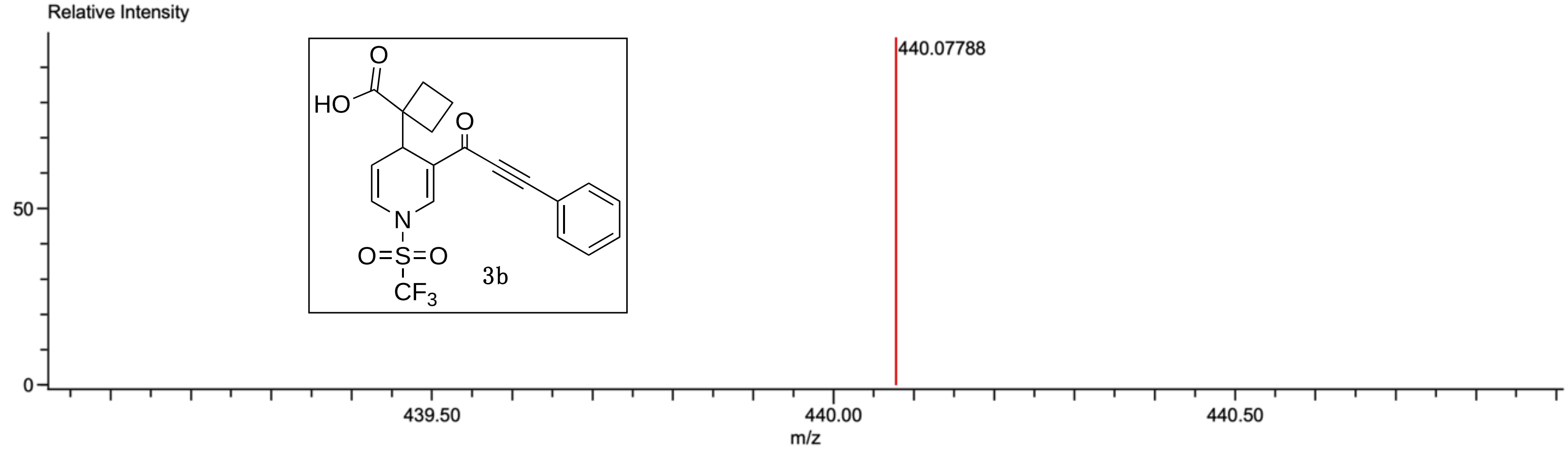

Mass	Intensity	Calc. Mass	Mass Difference (mmu)	Mass Difference (ppm)	Possible Formula	Unsaturation Number
440.07788	40138.38	440.07795	-0.08	-0.17	$^{12}C_{20}{}^{1}H_{17}{}^{19}F_{3}{}^{14}N_{1}{}^{16}O_{5}{}^{32}S_{1}$	12.5

Figure S8. HRMS-DART^{+} (19 eV) of **3b**.

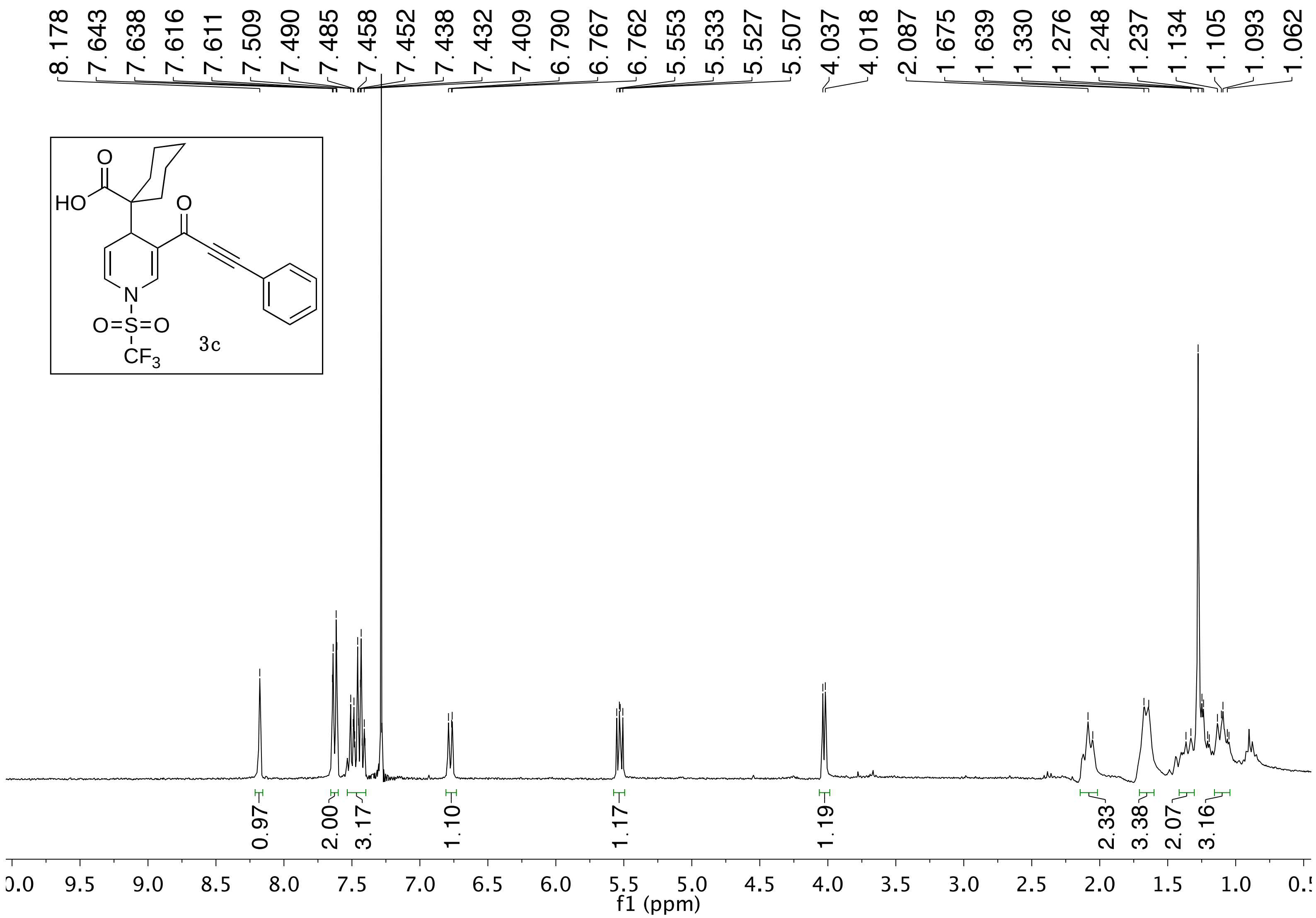

Figure S9. ^{1}H NMR (300 MHz/$CDCl_3$/TMS) of **3c**.

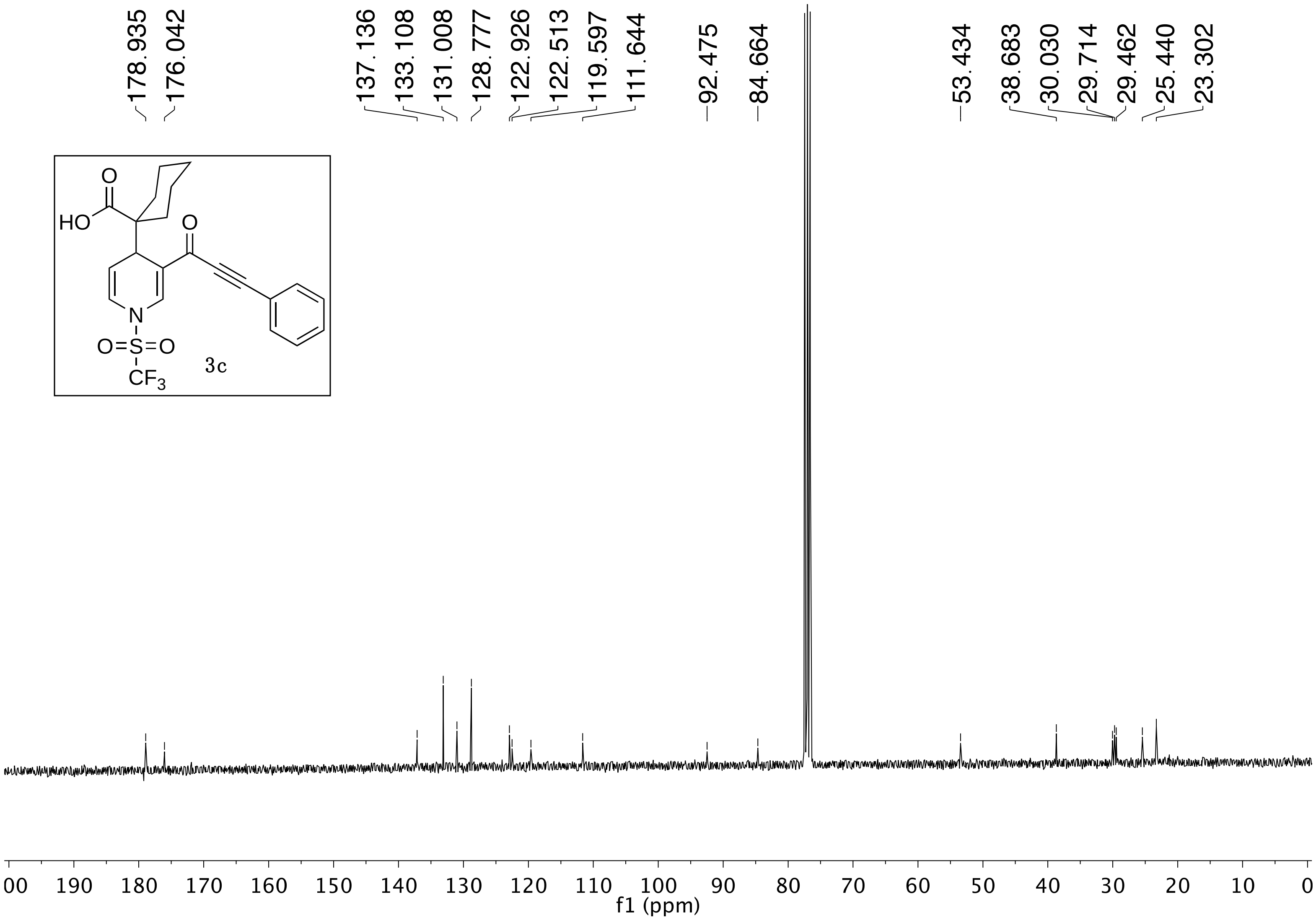

Figure S10. ^{13}C NMR (75 MHz/$CDCl_3$/TMS) of **3c**.

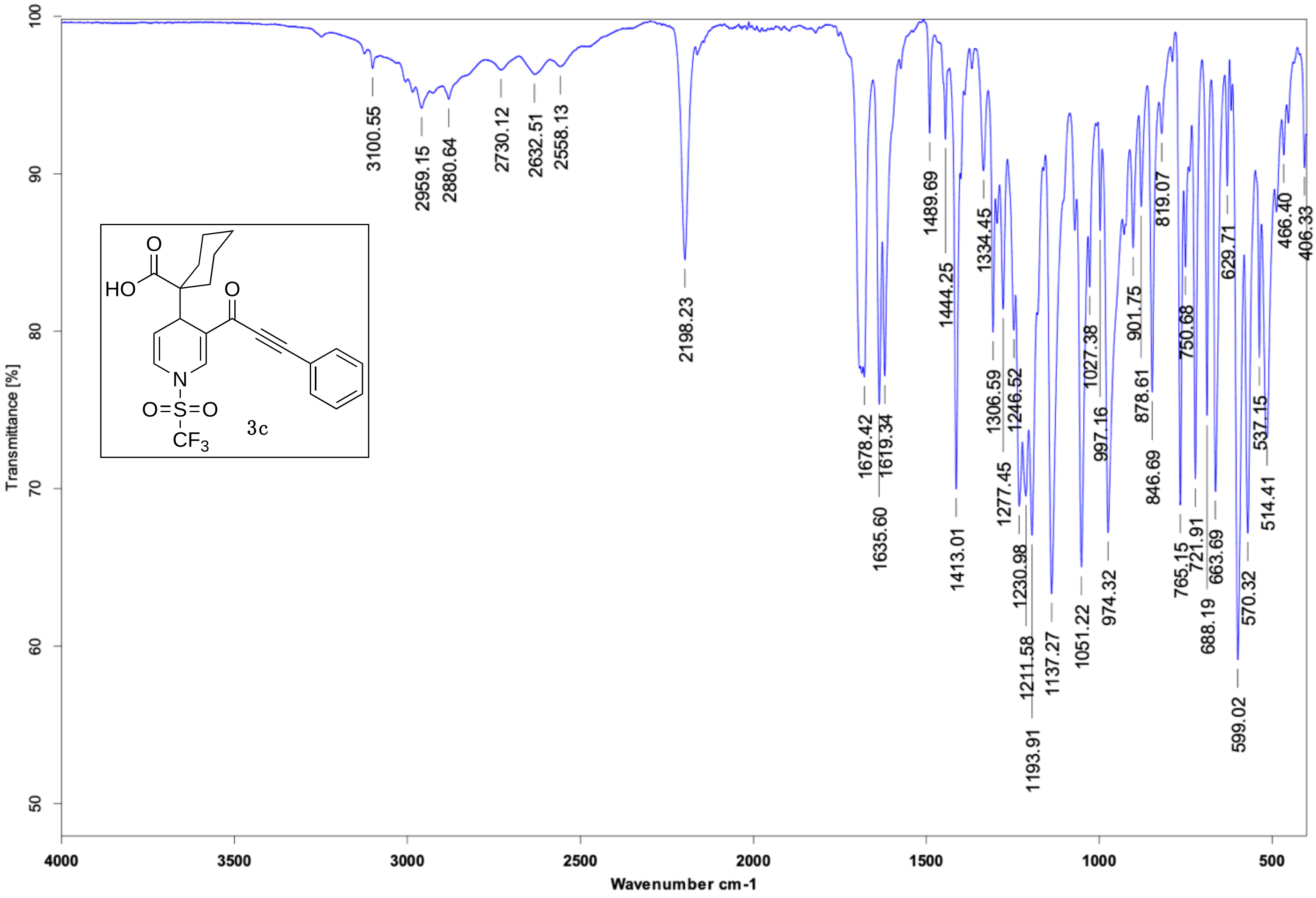

Figure S11. FTIR of **3c**.

Description:
Ionization Mode:ESI+
History:Determine m/z[Peak Detect[Centroid,30,Area];Correct Base[1.0%];Smooth[5]];Correct Base[5.0%];Average...

Mass Calibration data:Cal_Peg_600
Created:11/22/2018 1:54:44 PM
Created by:AccuTOF

Charge number:1
Tolerance:3.00(mmu)
Unsaturation Number:0.0 .. 50.0 (Fraction:Both)
Element:^{12}C:0 .. 22, ^{1}H:20 .. 21, ^{19}F:0 .. 3, ^{14}N:0 .. 1, ^{16}O:0 .. 5, ^{32}S:0 .. 1

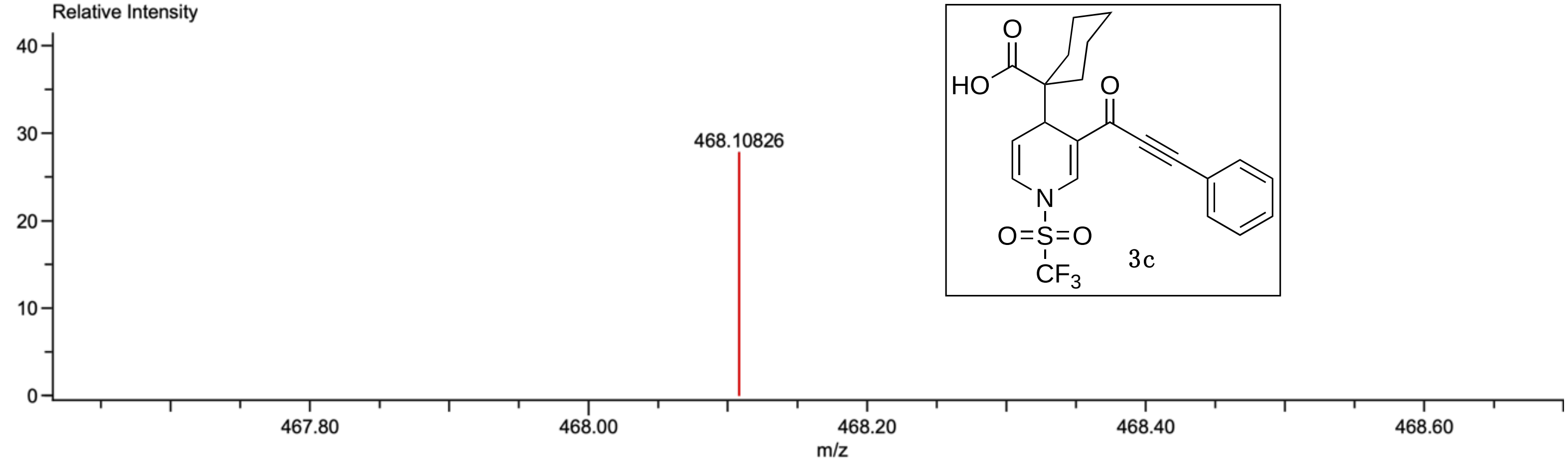

Mass	Intensity	Calc. Mass	Mass Difference (mmu)	Mass Difference (ppm)	Possible Formula	Unsaturation Number
468.10826	337932.81	468.10925	-0.99	-2.12	$^{12}C_{22}{}^{1}H_{21}{}^{19}F_{3}{}^{14}N_{1}{}^{16}O_{5}{}^{32}S_{1}$	12.5

Figure S12. HRMS-DART$^+$ (19 eV) of **3c**.

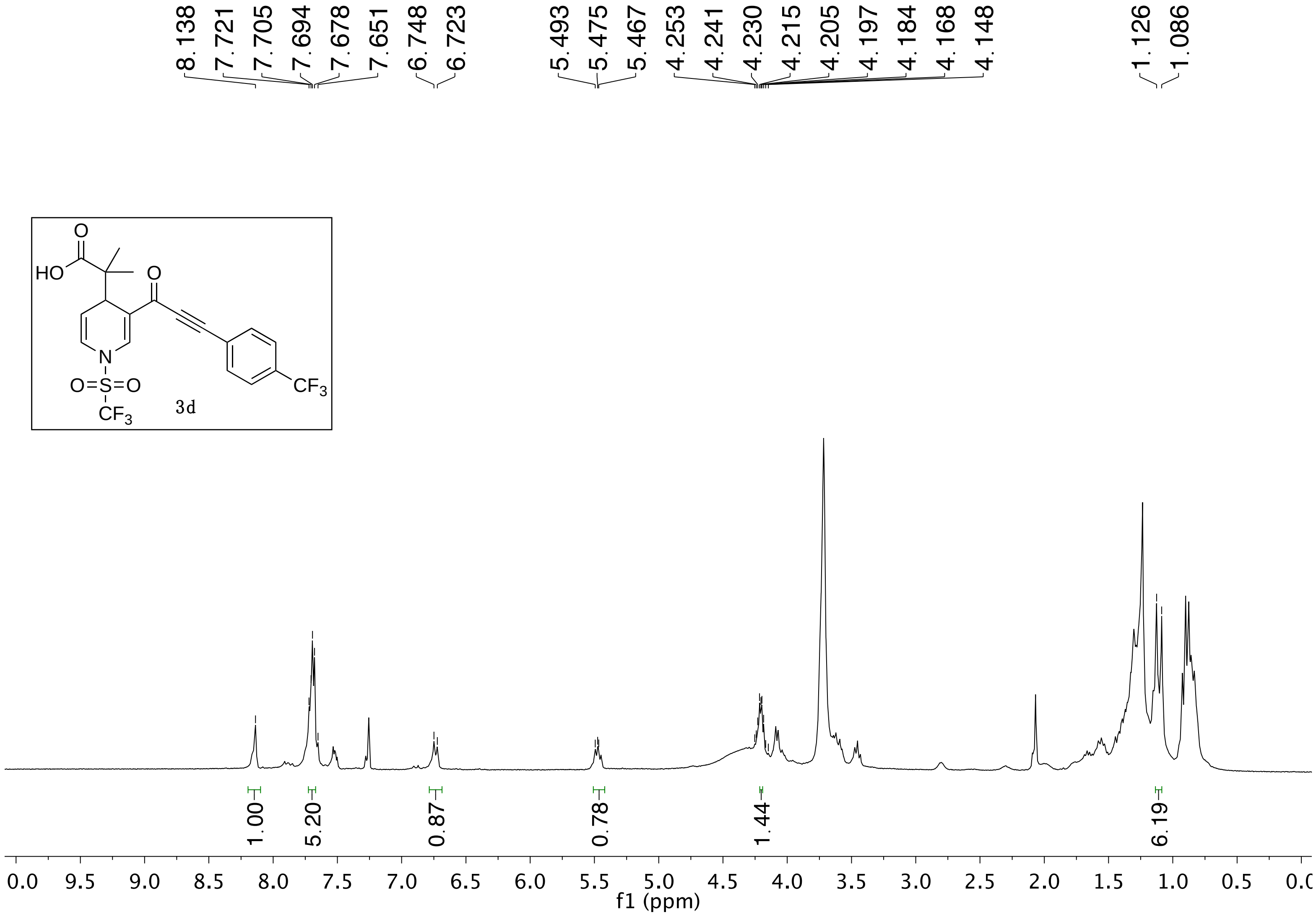

Figure S13. ^{1}H NMR (300 MHz/CDCl$_3$/TMS) of **3d**.

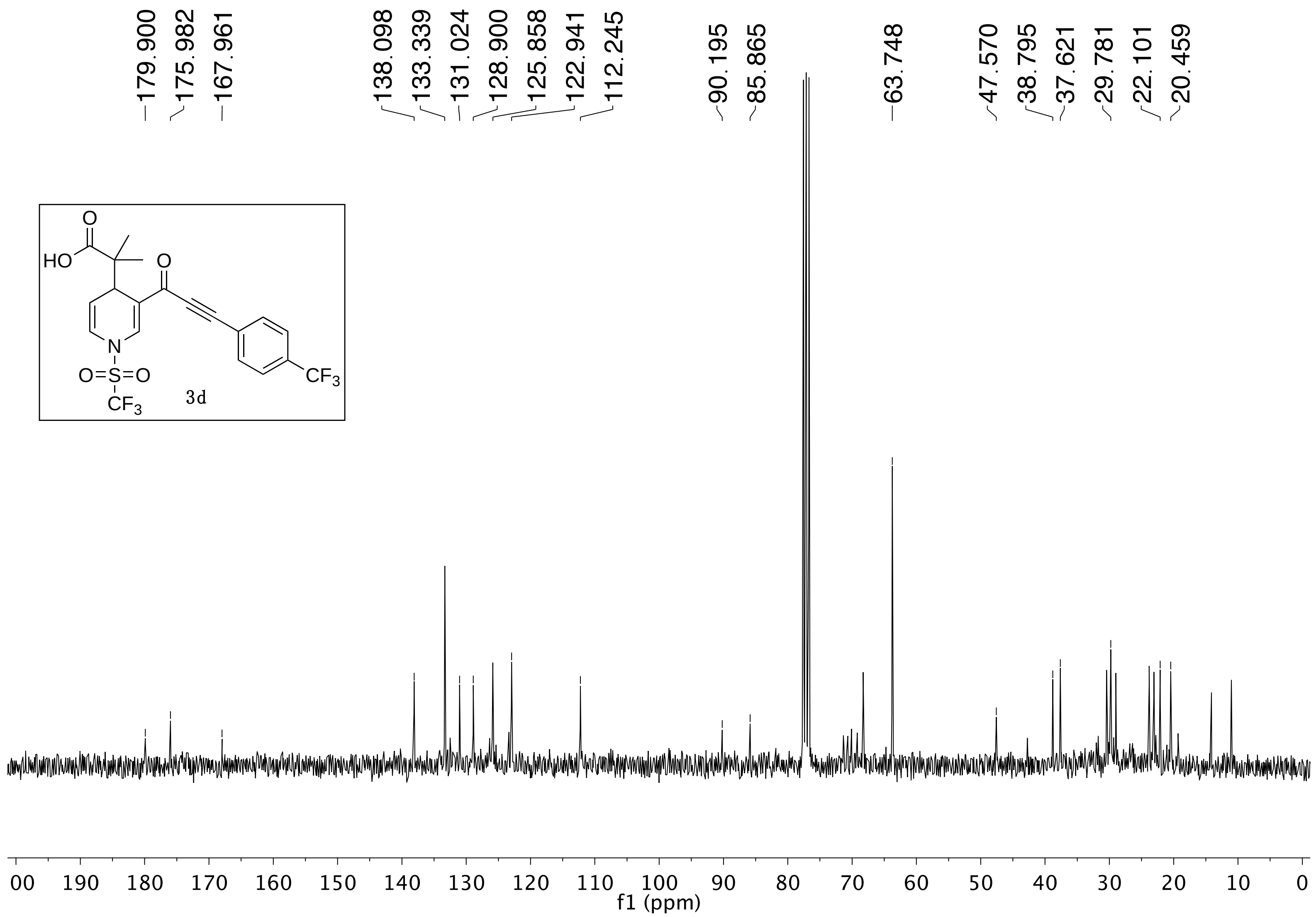

Figure S14. ^{13}C NMR (75 MHz/$CDCl_3$/TMS) of **3d**.

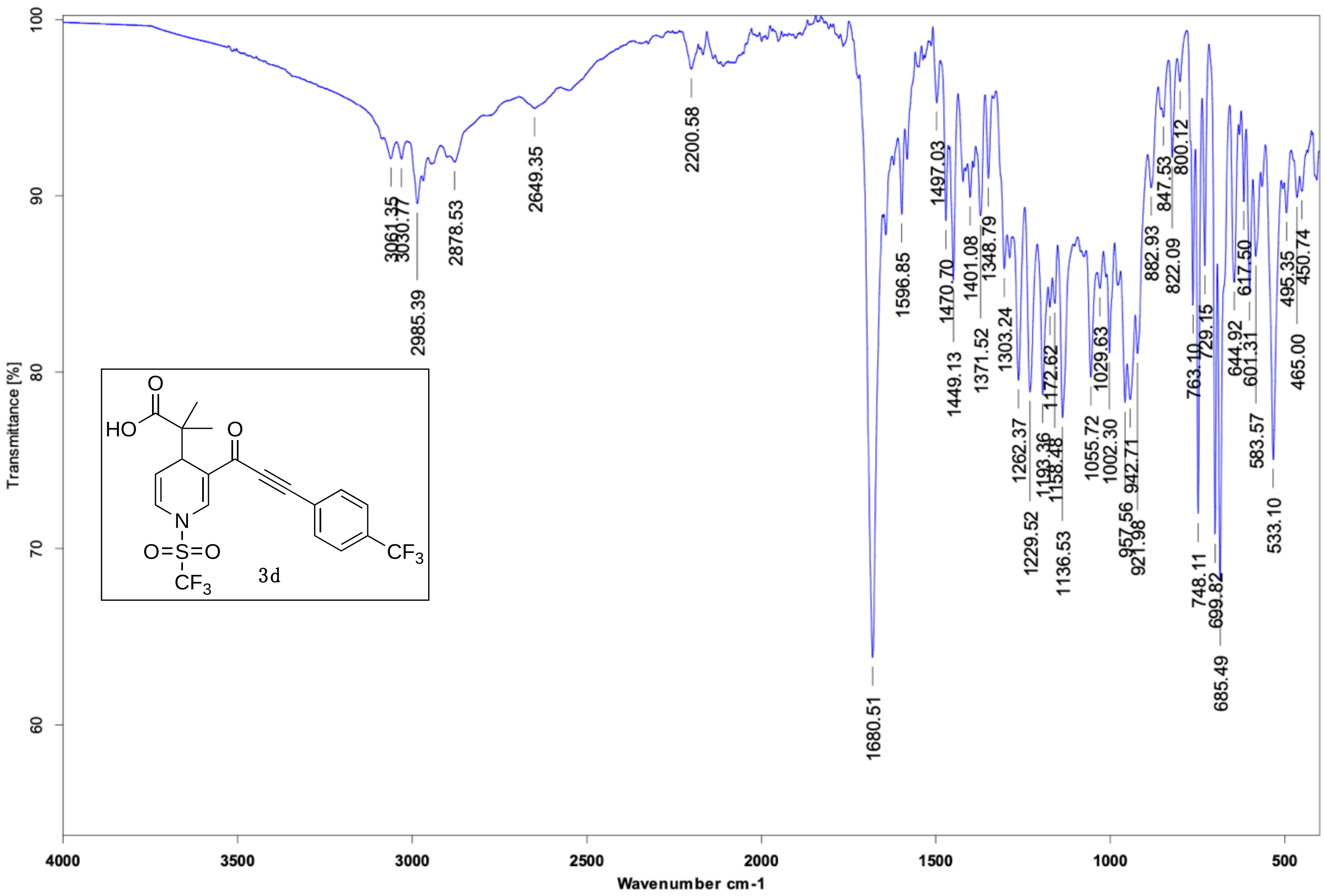

Figure S15. FTIR of **3d**.

Description:
Ionization Mode:ESI+
History:Determine m/z[Peak Detect[Centroid,30,Area];Correct Base[1.0%];Smooth[5]];Correct Base[5.0%];Average...

Mass Calibration data:Cal_Peg_600
Created:11/22/2018 1:48:38 PM
Created by:AccuTOF

Charge number:1
Tolerance:3.00(mmu)
Unsaturation Number:0.0 .. 50.0 (Fraction:Both)
Element:^{12}C:0 .. 20, ^{1}H:0 .. 16, ^{19}F:0 .. 6, ^{14}N:0 .. 1, ^{16}O:0 .. 5, ^{32}S:0 .. 1

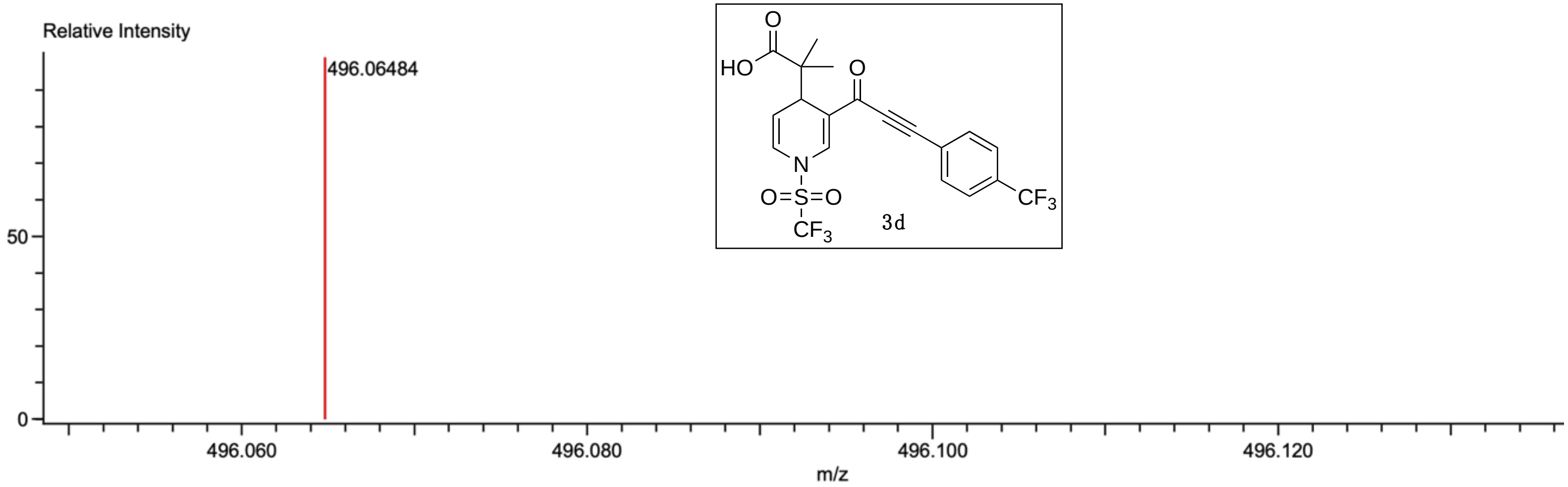

Mass	Intensity	Calc. Mass	Mass Difference (mmu)	Mass Difference (ppm)	Possible Formula	Unsaturation Number
496.06484	877924.67	496.06534	-0.50	-1.01	$^{12}C_{20}{}^{1}H_{16}{}^{19}F_{6}{}^{14}N_{1}{}^{16}O_{5}{}^{32}S_{1}$	11.5

Figure S16. HRMS-DART+ (19 eV) of **3d**.

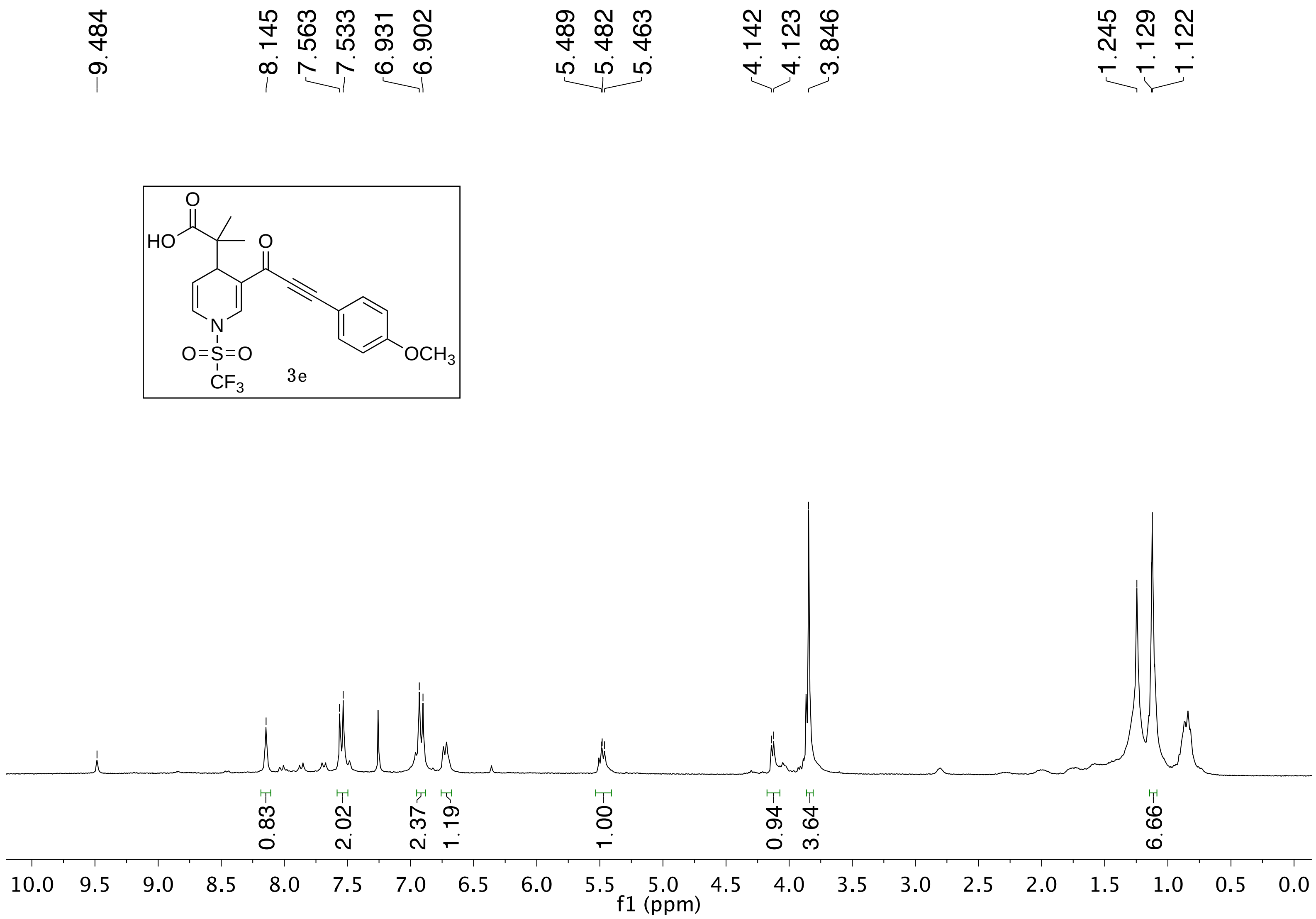

Figure S17. ^{1}H NMR (300 MHz/$CDCl_3$/TMS) of **3e**.

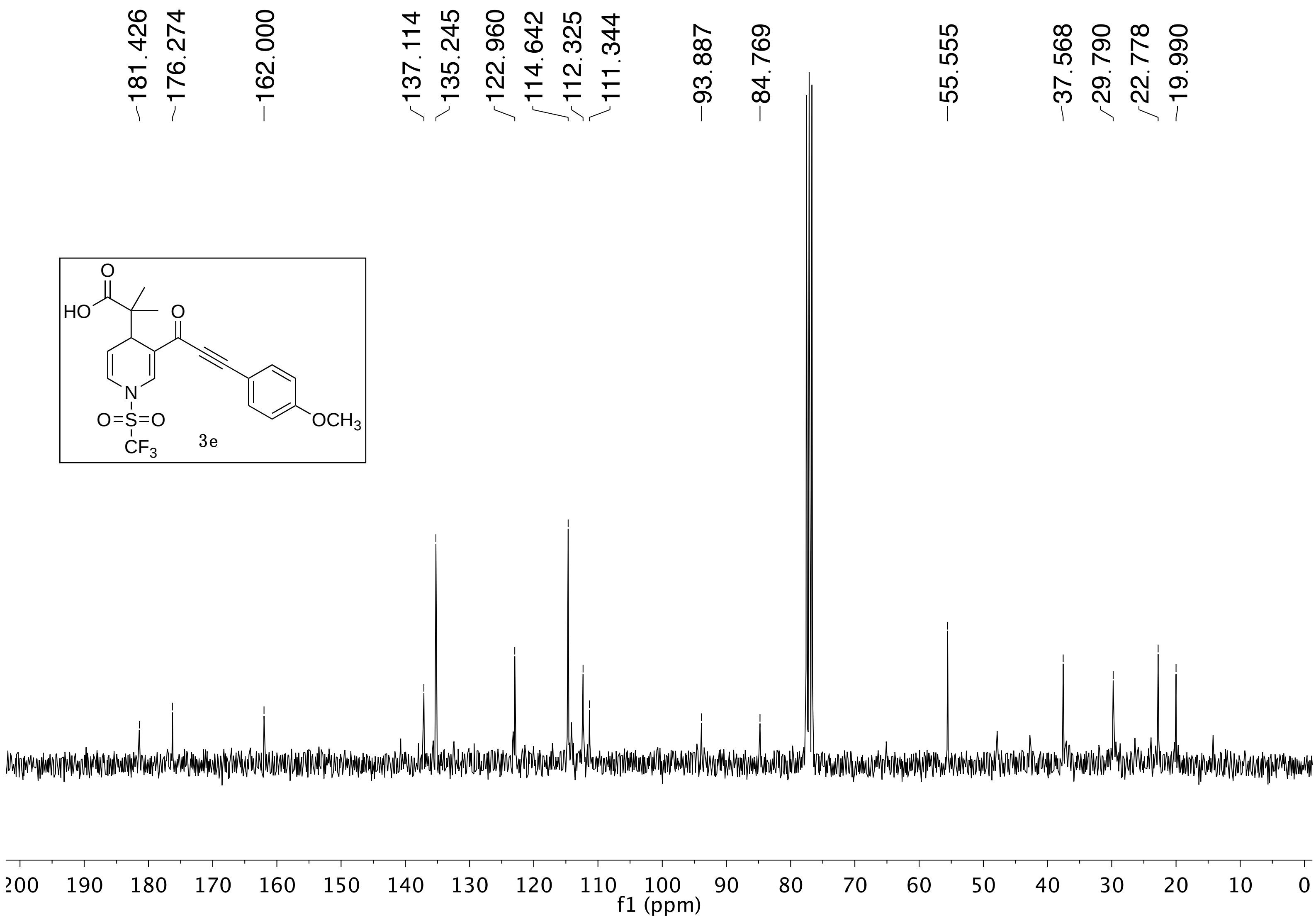

Figure S18. ^{13}C NMR (75 MHz/$CDCl_3$/TMS) of **3e**.

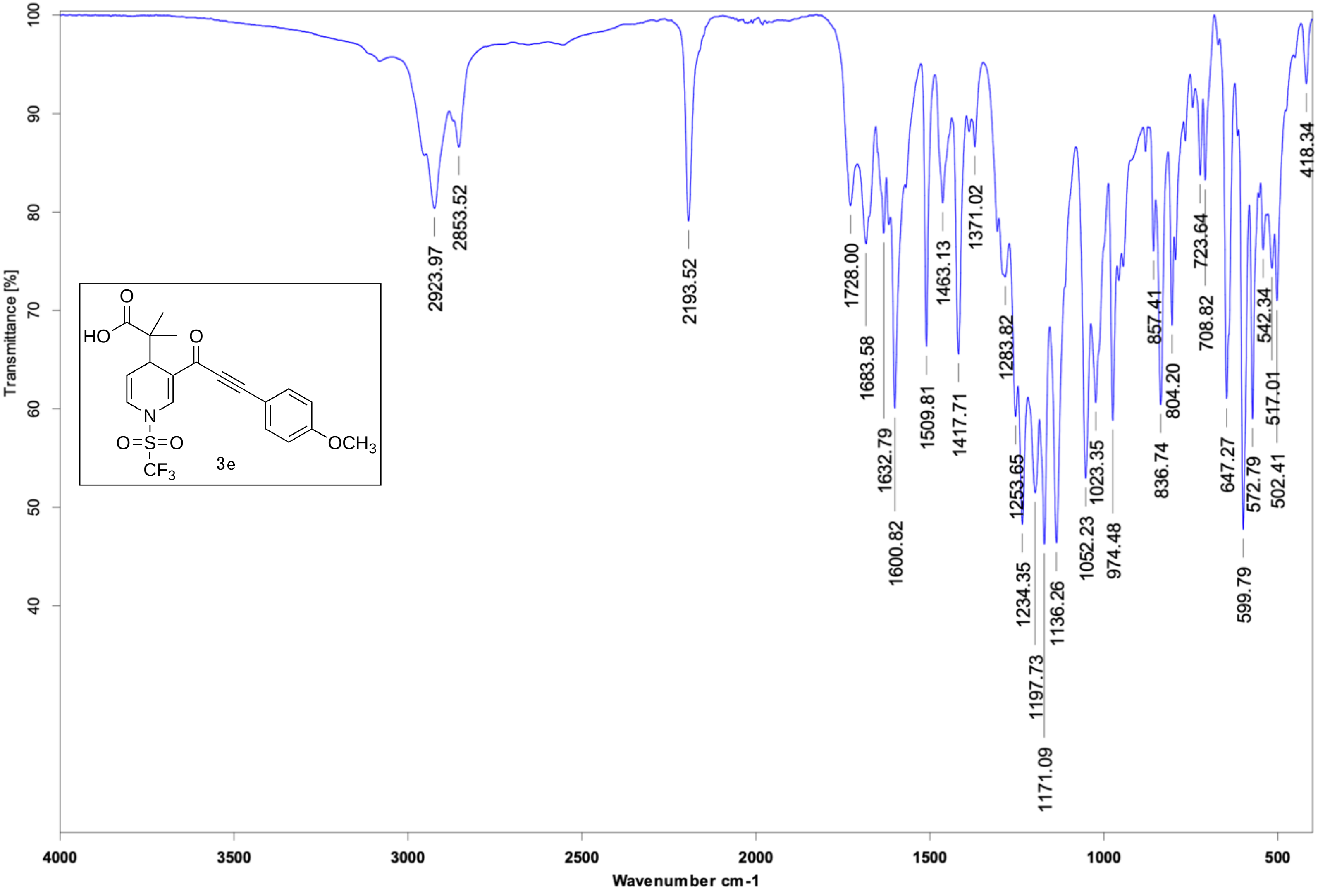

Figure S19. FTIR of **3e**.

Description:
Ionization Mode:ESI+
History:Determine m/z[Peak Detect[Centroid,30,Area];Correct Base[];Smooth[5]];Correct Base[5.0%];Average(MS[...

Mass Calibration data:Cal_PEG_600
Created:10/6/2022 11:33:09 AM
Created by:AccuTOF

Charge number:1
Tolerance:5.00(mmu)
Unsaturation Number:0.0 .. 50.0 (Fraction:Both)
Element:^{12}C:0 .. 20, ^{1}H:0 .. 22, ^{19}F:0 .. 3, ^{14}N:0 .. 1, ^{16}O:1 .. 6, ^{32}S:1 .. 1

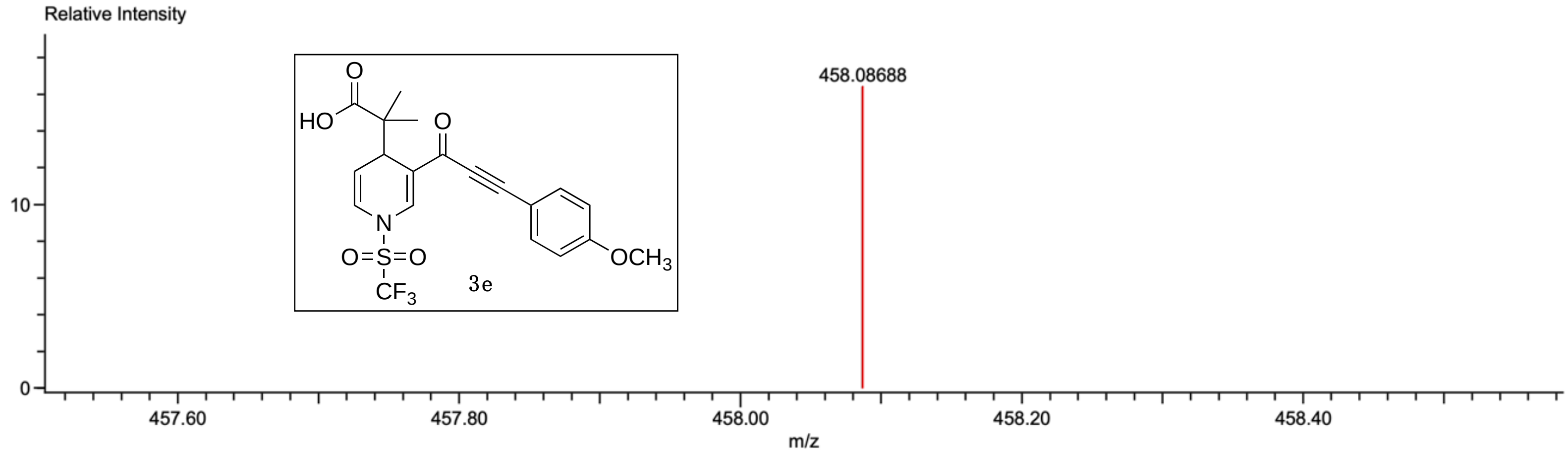

Mass	Intensity	Calc. Mass	Mass Difference (mmu)	Mass Difference (ppm)	Possible Formula	Unsaturation Number
458.08688	4525.12	458.08852	-1.64	-3.57	$^{12}C_{20}{}^{1}H_{19}{}^{19}F_{3}{}^{14}N_{1}{}^{16}O_{6}{}^{32}S_{1}$	11.5

Figure S20. HRMS-DART^{+} (19 eV) of **3e**.

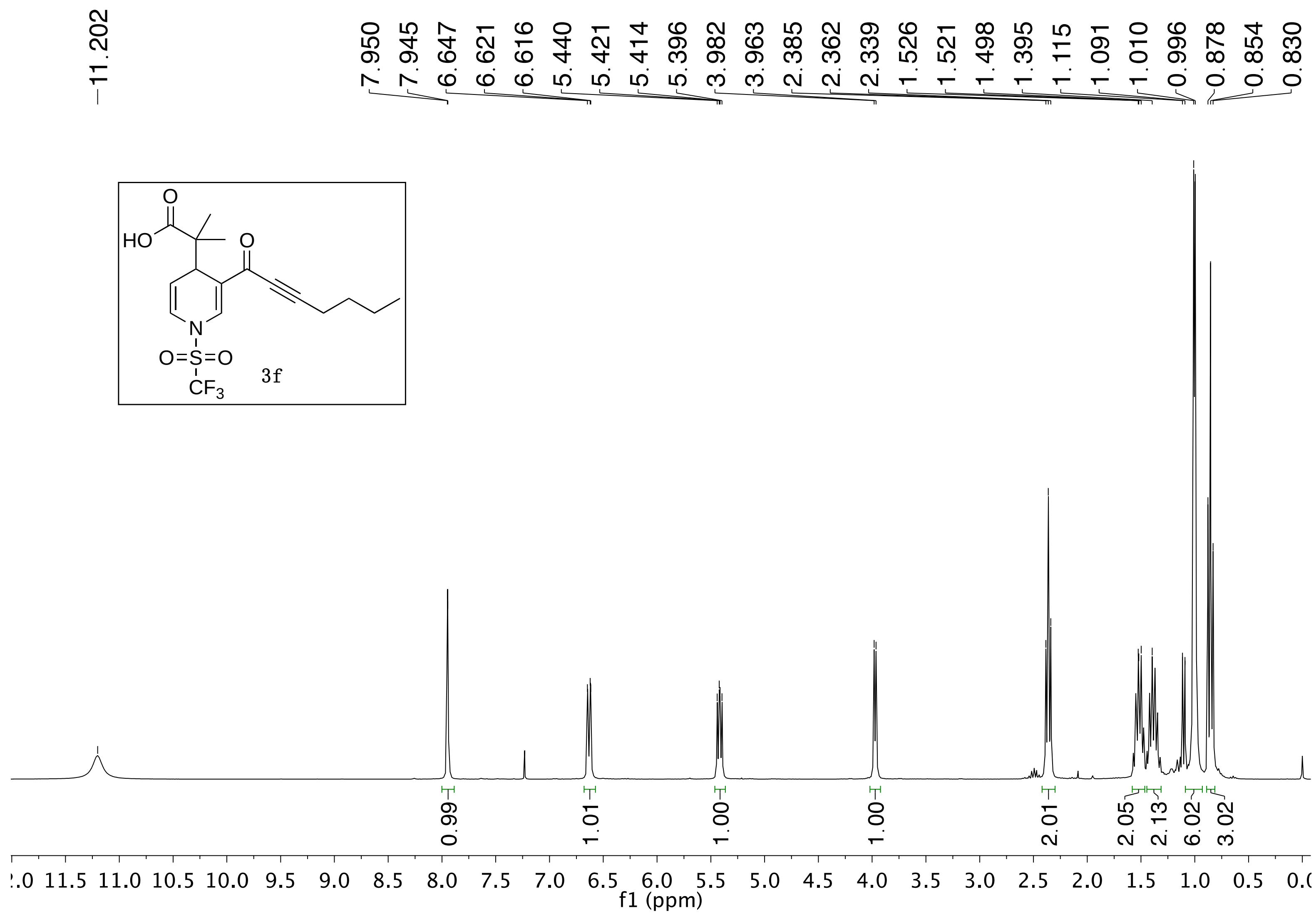

Figure S21. ^{1}H NMR (300 MHz/$CDCl_3$/TMS) of **3f**.

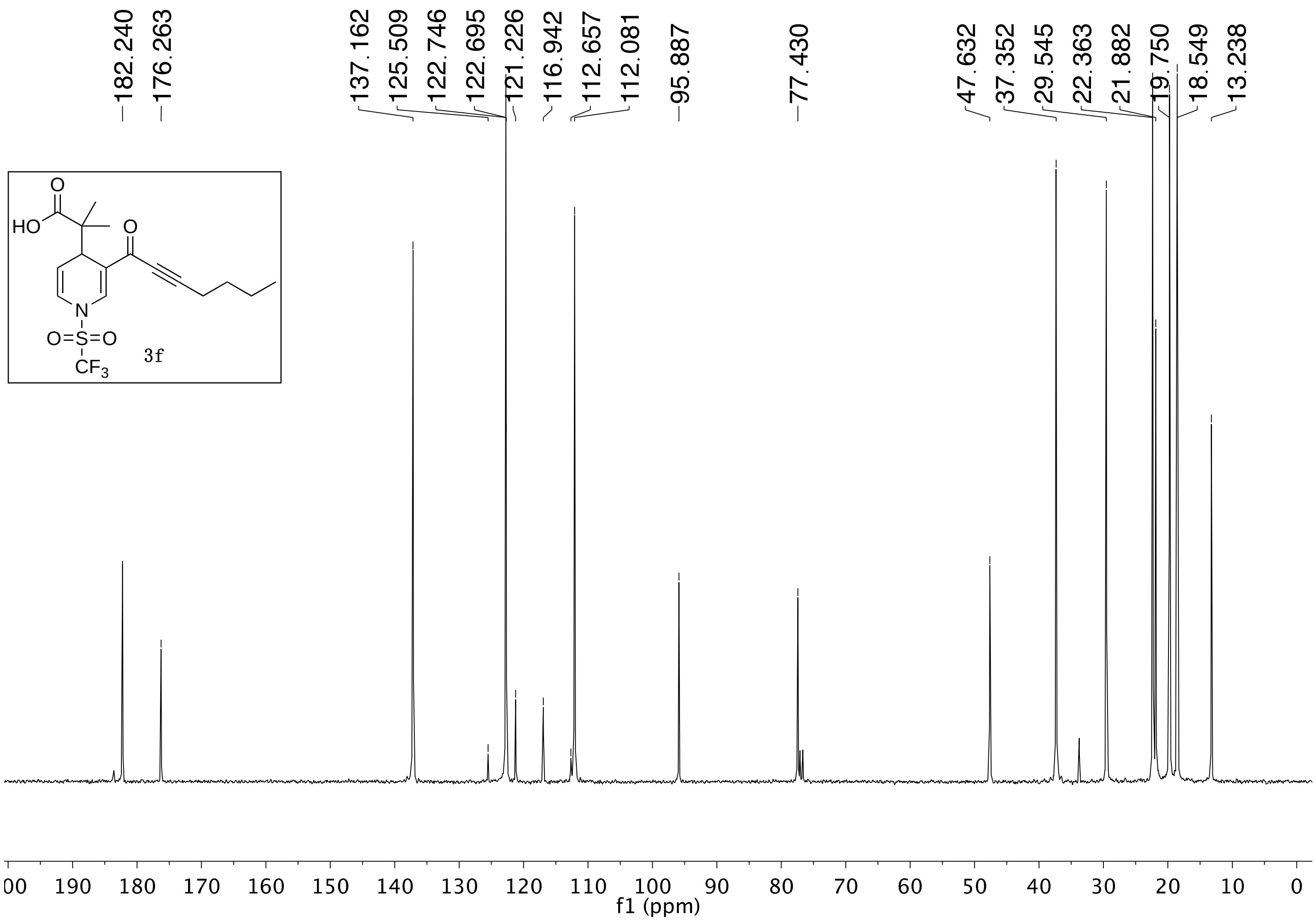

Figure S22. ^{13}C NMR (75 MHz/$CDCl_3$/TMS) of **3f**.

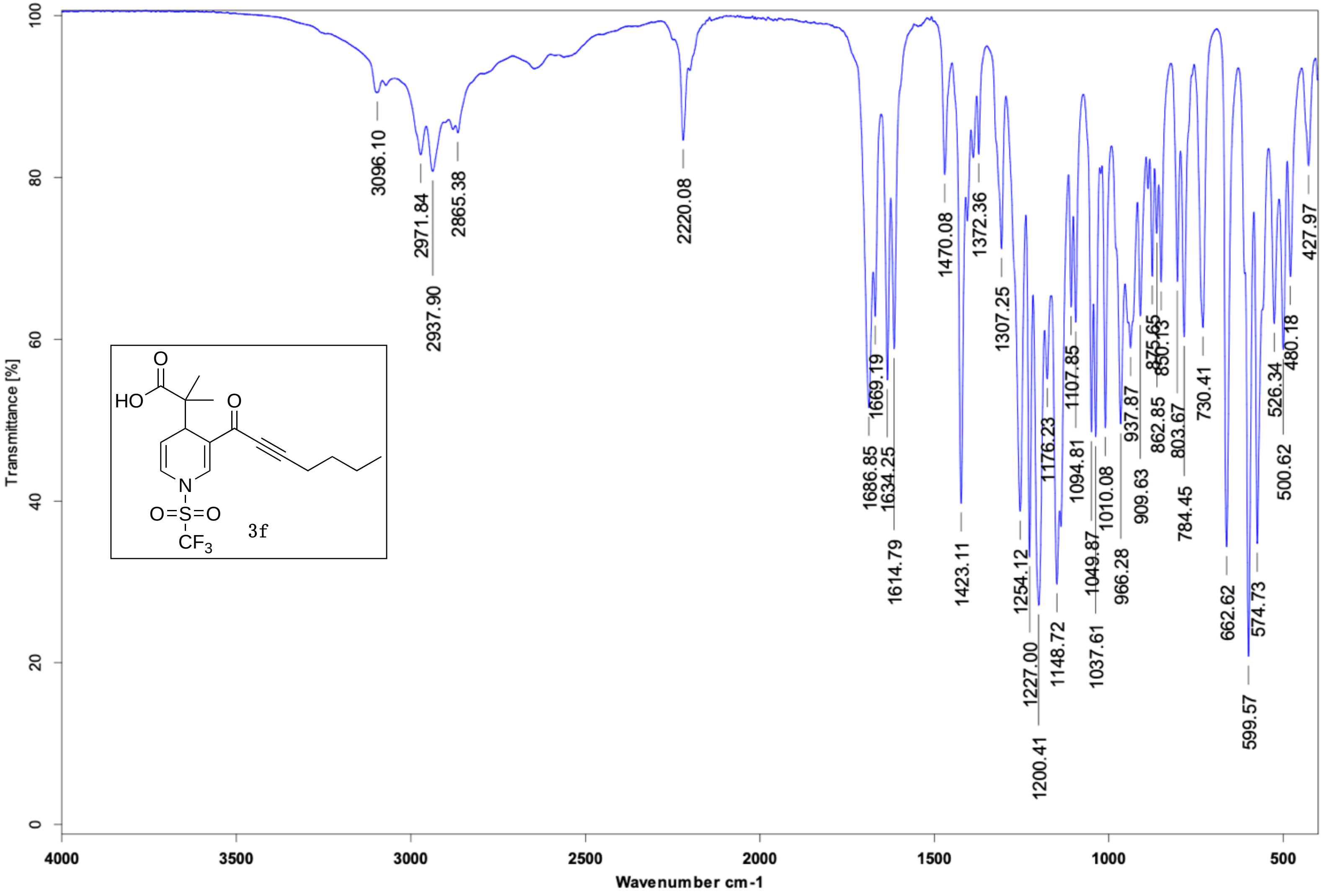

Figure S23. FTIR of **3f**.

Description:
Ionization Mode:ESI+
History:Determine m/z[Peak Detect[Centroid,30,Area];Correct Base[1.0%];Smooth[5]];Correct Base[5.0%];Average...

Mass Calibration data:Cal_Peg_600
Created:11/28/2018 2:23:02 PM
Created by:AccuTOF

Charge number:1
Tolerance:100.00(mmu)
Unsaturation Number:0.0 .. 50.0 (Fraction:Both)
Element:^{12}C:0 .. 17, ^{1}H:0 .. 21, ^{19}F:1 .. 3, ^{14}N:1 .. 1, ^{16}O:0 .. 5, ^{32}S:0 .. 1

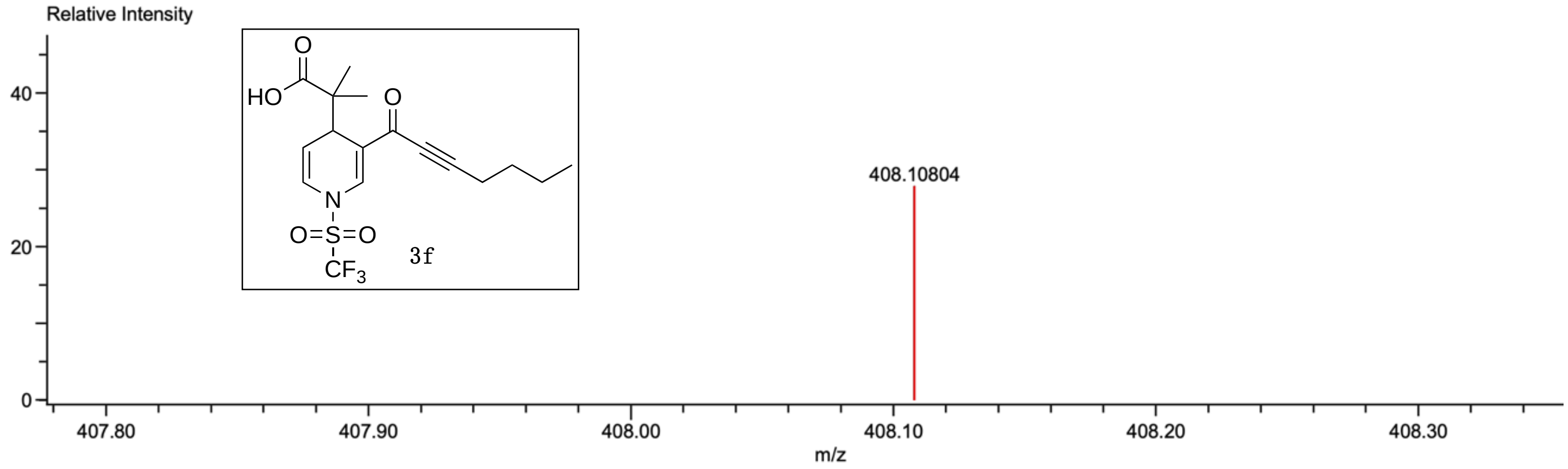

Mass	Intensity	Calc. Mass	Mass Difference (mmu)	Mass Difference (ppm)	Possible Formula	Unsaturation Number
408.10804	345096.48	408.10925	-1.21	-2.97	$^{12}C_{17}{}^{1}H_{21}{}^{19}F_{3}{}^{14}N_{1}{}^{16}O_{5}{}^{32}S_{1}$	7.5

Figure S24. HRMS-DART^{+} (19 eV) of **3f**.

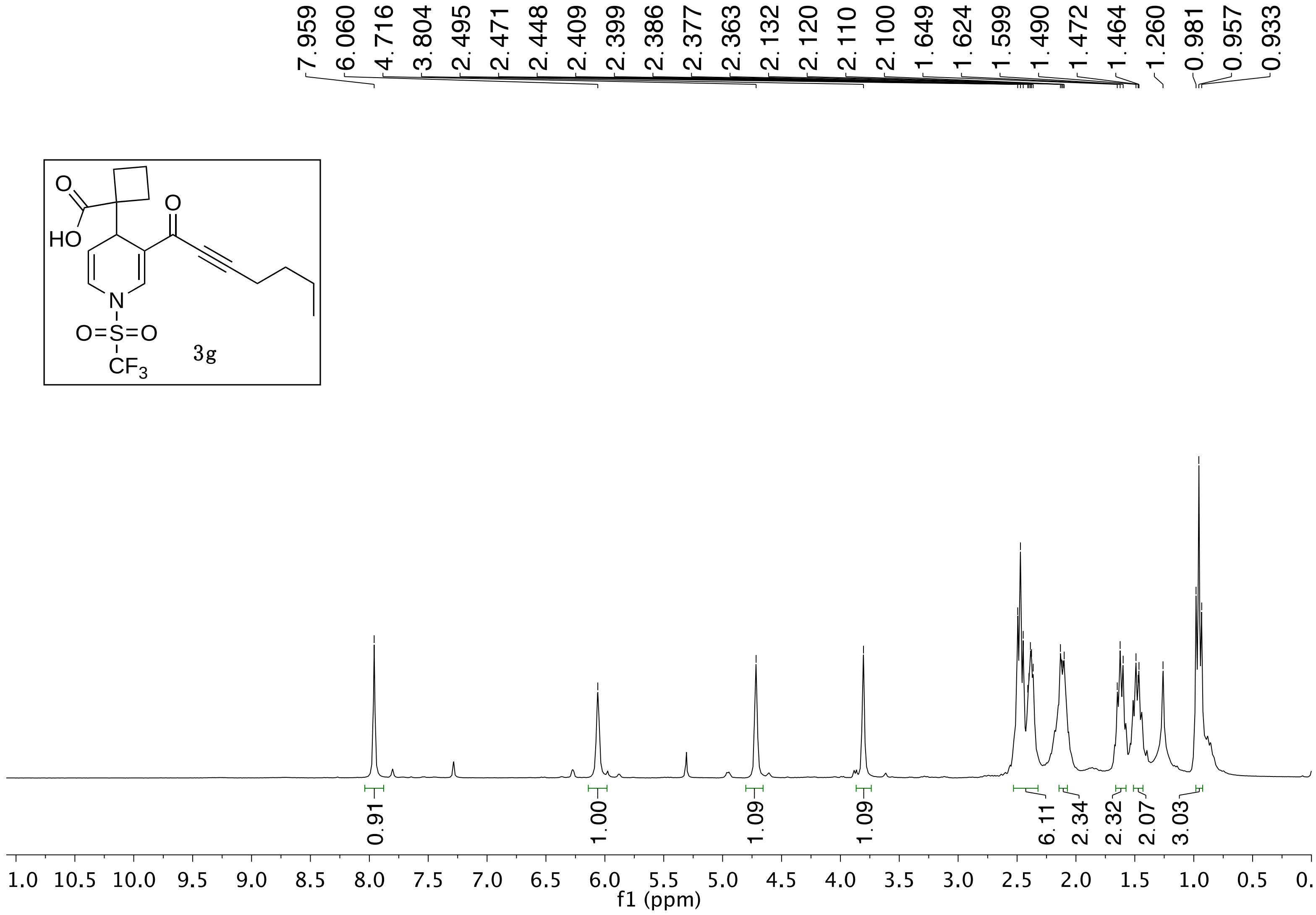

Figure S25. ^{1}H NMR (300 MHz/$CDCl_3$/TMS) of **3g**.

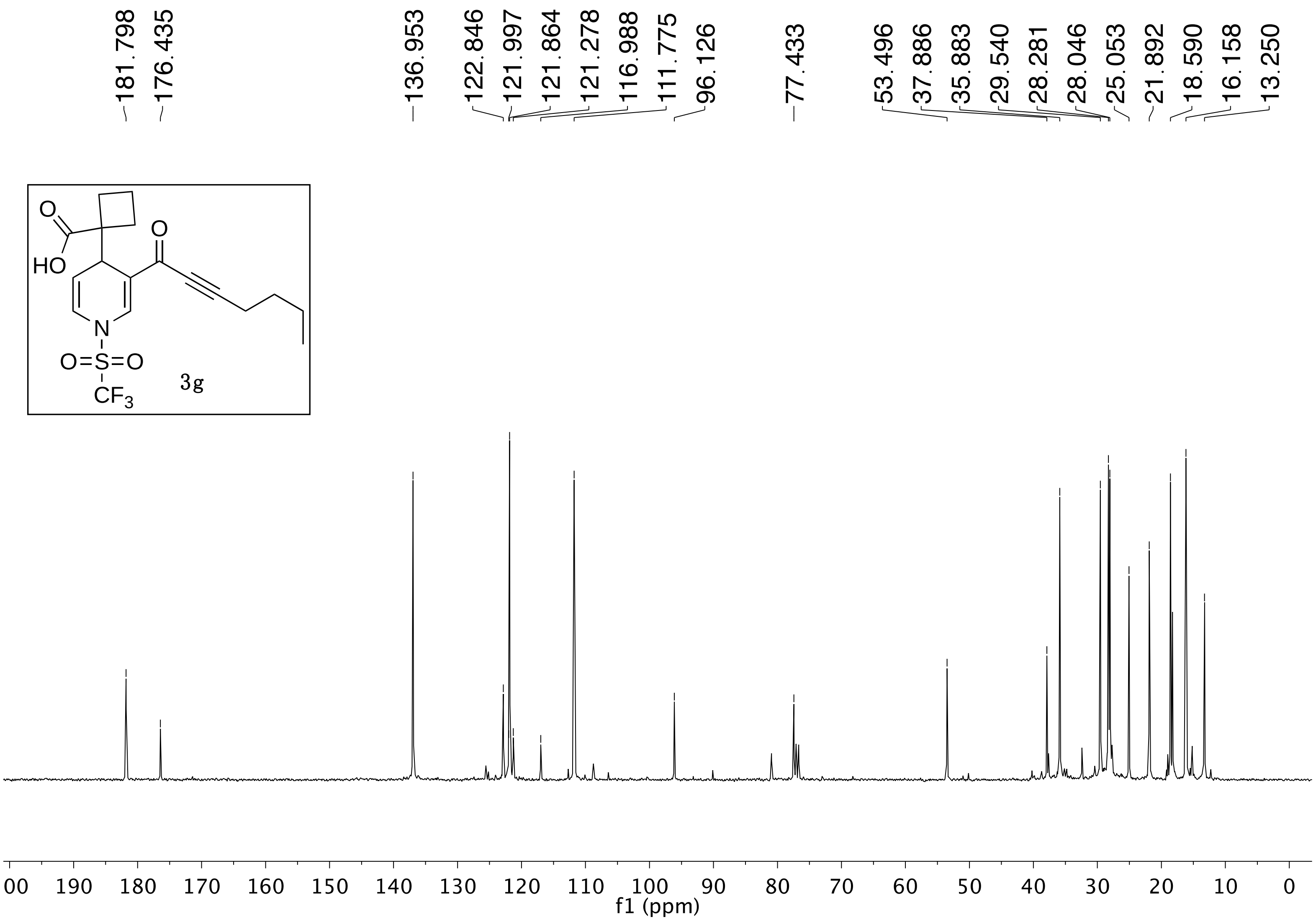

Figure S26. ^{13}C NMR (75 MHz/$CDCl_3$/TMS) of **3g**.

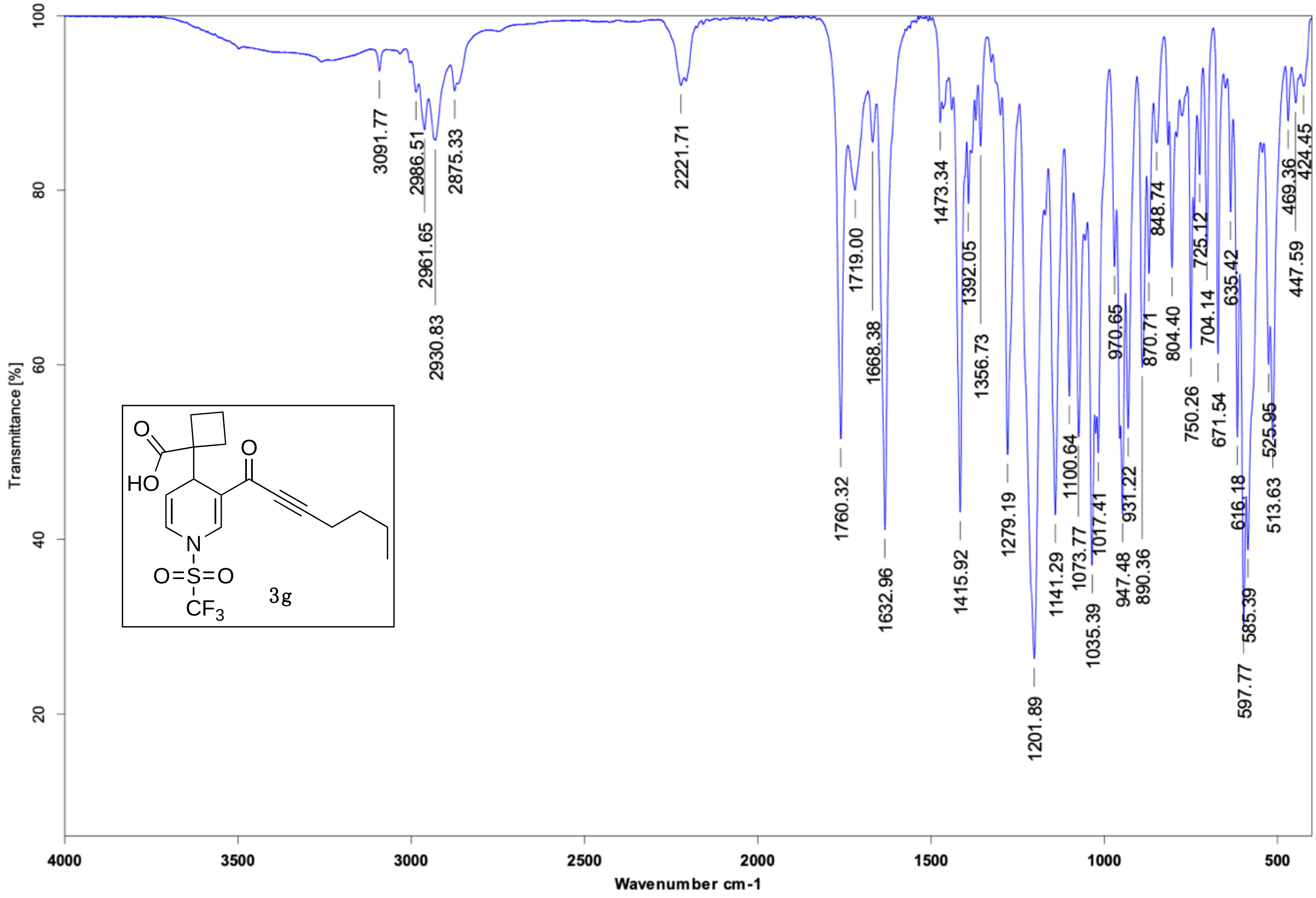

Figure S27. FTIR of **3g**.

Description:
Ionization Mode:ESI+
History:Determine m/z[Peak Detect[Centroid,30,Area];Correct Base[1.0%];Smooth[5]];Correct Base[5.0%];Average...

Mass Calibration data:Cal_Peg_600
Created:11/28/2018 2:25:40 PM
Created by:AccuTOF

Charge number:1
Tolerance:100.00(mmu)
Unsaturation Number:0.0 .. 50.0 (Fraction:Both)
Element:^{12}C:0 .. 18, ^{1}H:0 .. 21, ^{19}F:1 .. 3, ^{14}N:1 .. 1, ^{16}O:0 .. 5, ^{32}S:0 .. 1

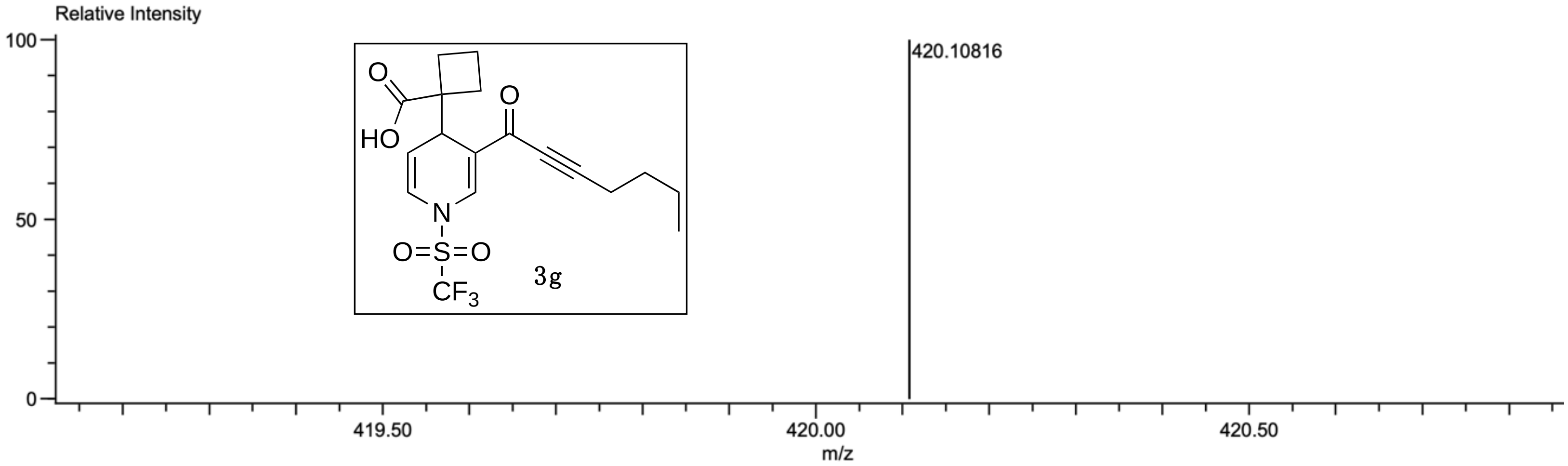

Mass	Intensity	Calc. Mass	Mass Difference (mmu)	Mass Difference (ppm)	Possible Formula	Unsaturation Number
420.10816	415259.35	420.10925	-1.09	-2.60	$^{12}C_{18}{}^{1}H_{21}{}^{19}F_{3}{}^{14}N_{1}{}^{16}O_{5}{}^{32}S_{1}$	8.5

Figure S28. HRMS-DART^{+} (19 eV) of **3g**.

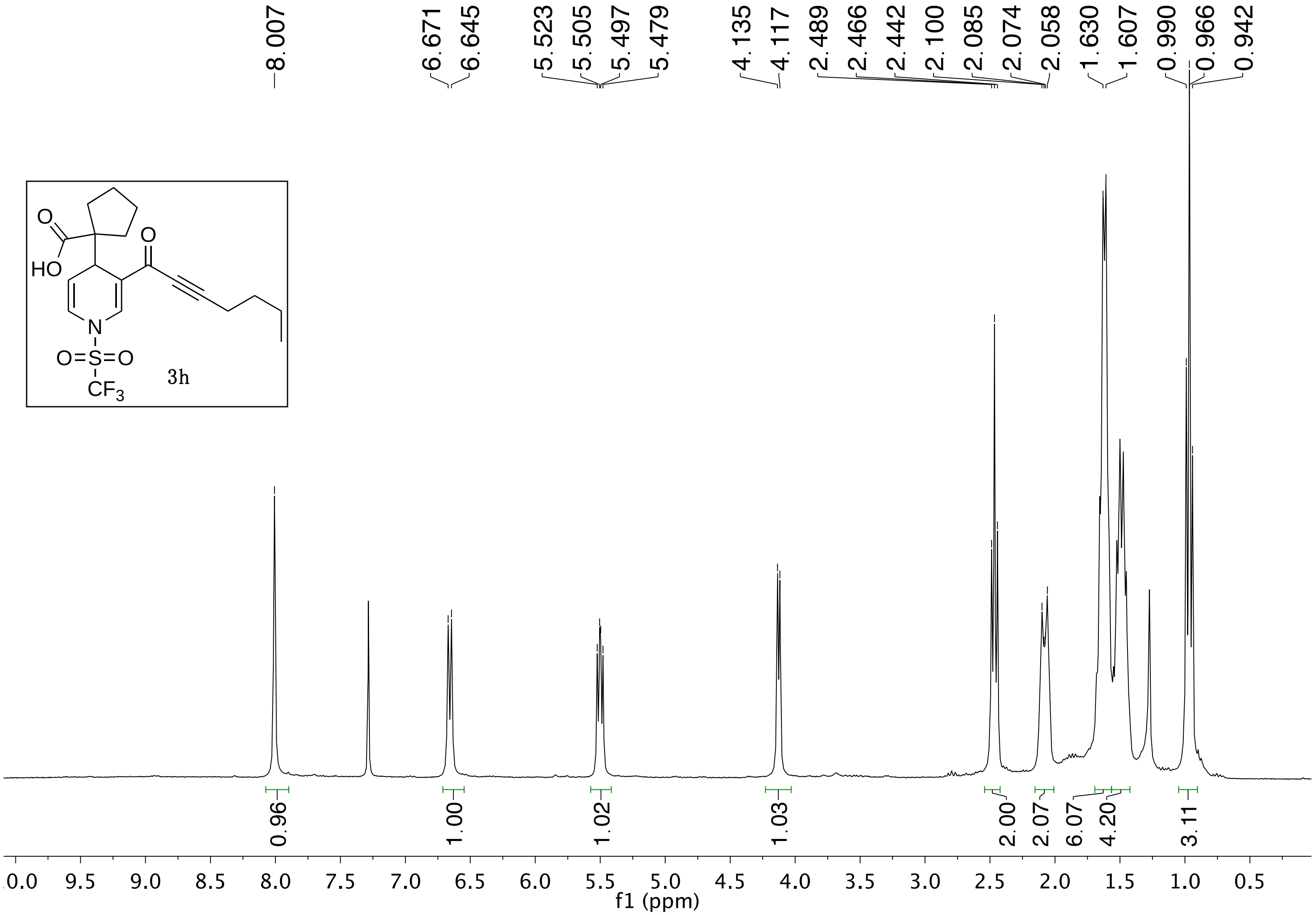

Figure S29. ^{1}H NMR (300 MHz/$CDCl_3$/TMS) of **3h**.

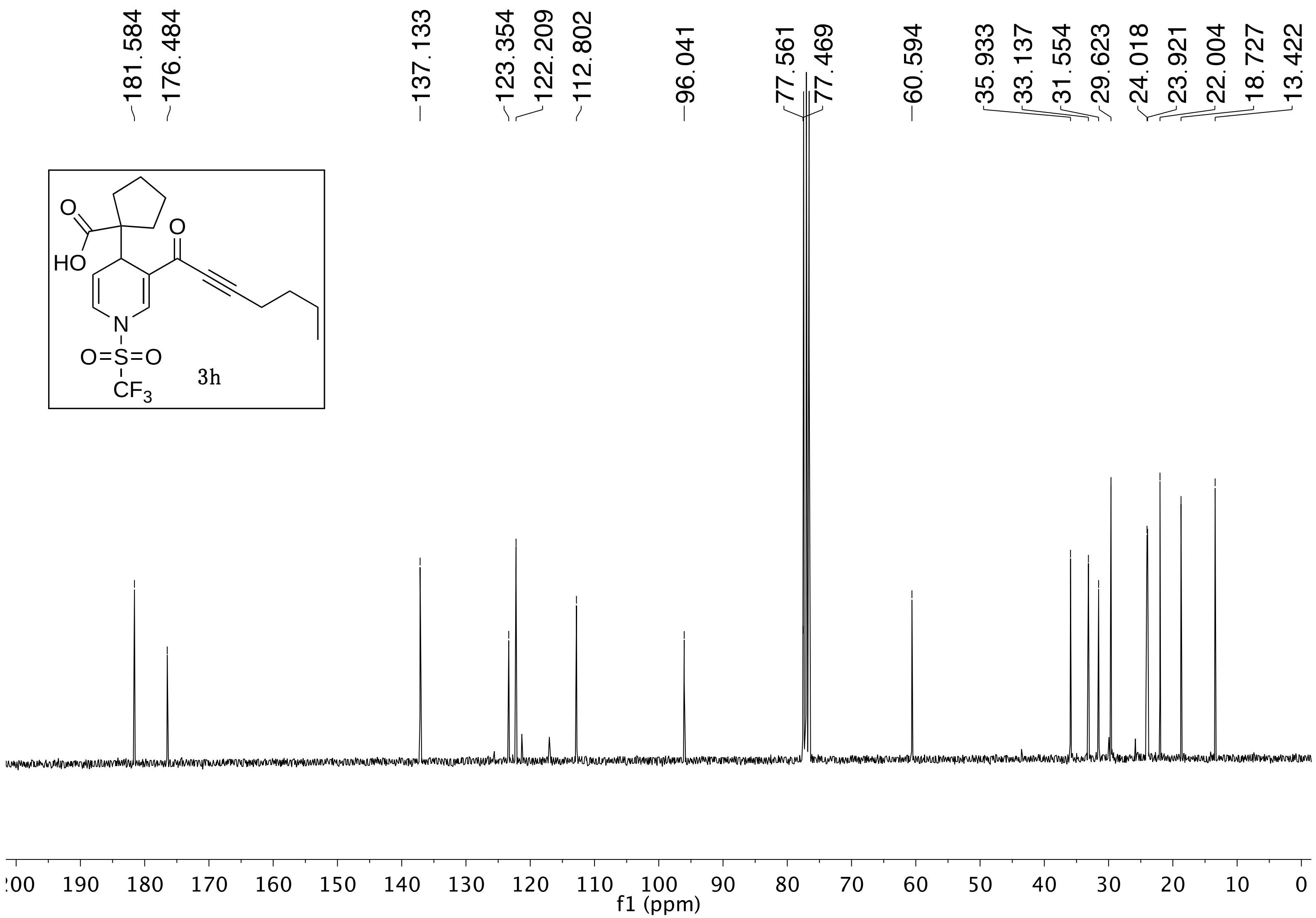

Figure S30. ^{13}C NMR (75 MHz/$CDCl_3$/TMS) of **3h**.

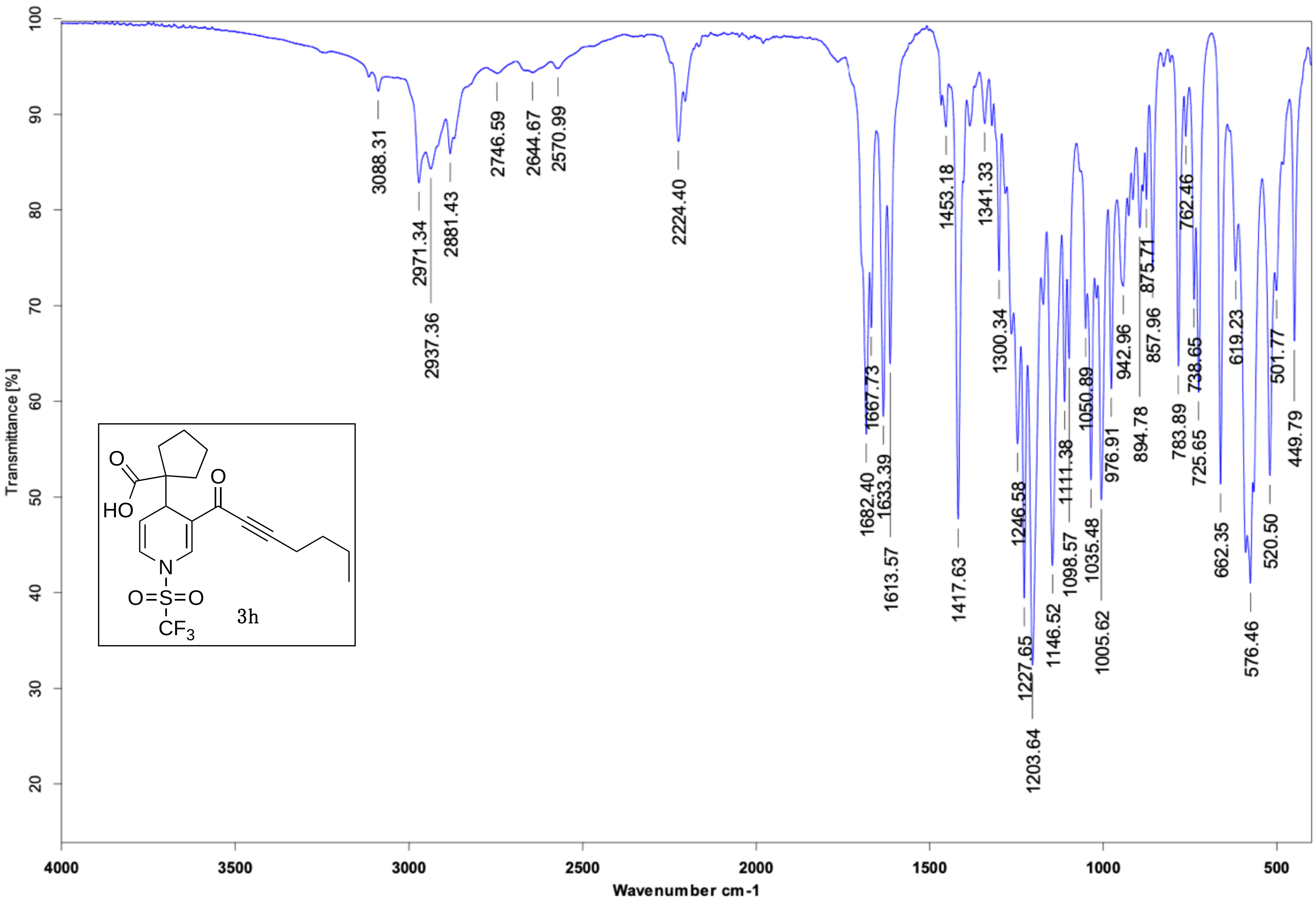

Figure S31. FTIR of **3h**.

Description:
Ionization Mode:ESI+
History:Determine m/z[Peak Detect[Centroid,30,Area];Correct Base[1.0%];Smooth[5]];Correct Base[5.0%];Average...

Mass Calibration data:Cal_Peg_600
Created:11/30/2018 11:23:52 AM
Created by:AccuTOF

Charge number:1 Tolerance:3.00(mmu) Unsaturation Number:0.0 .. 50.0 (Fraction:Both)

Element:^{12}C:0 .. 19, ^{1}H:0 .. 23, ^{19}F:1 .. 3, ^{14}N:0 .. 1, ^{16}O:0 .. 5, ^{32}S:1 .. 1

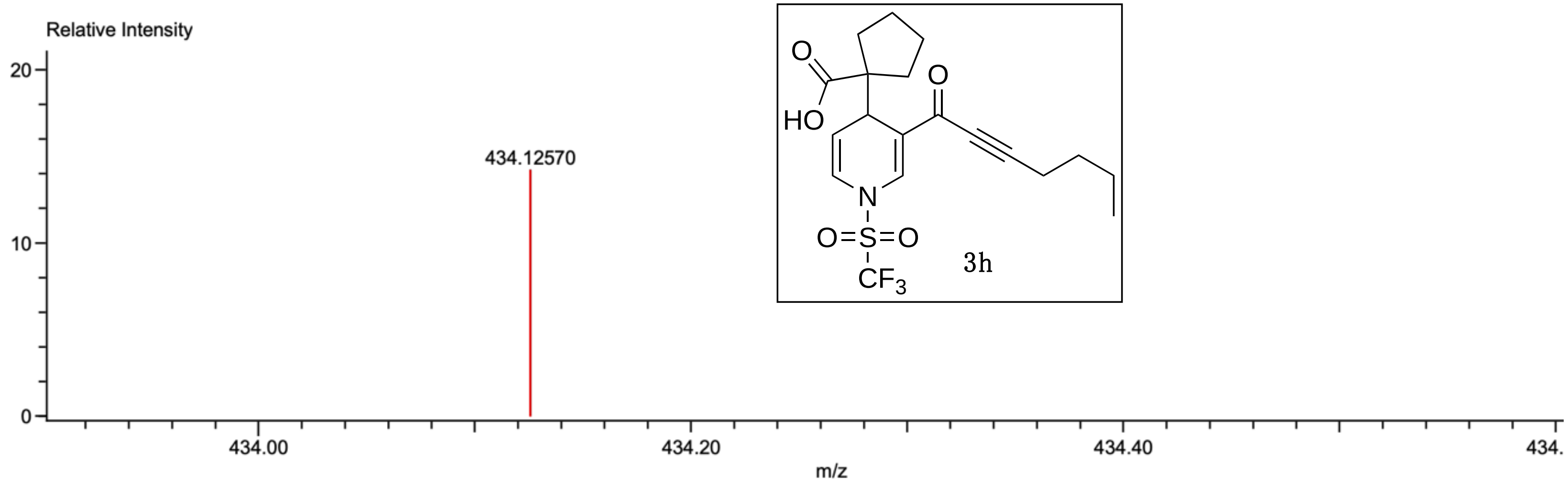

3h

Mass	Intensity	Calc. Mass	Mass Difference (mmu)	Mass Difference (ppm)	Possible Formula	Unsaturation Number
434.12570	10662.18	434.12490	0.80	1.85	$^{12}C_{19}{}^{1}H_{23}{}^{19}F_{3}{}^{14}N_{1}{}^{16}O_{5}{}^{32}S_{1}$	8.5

Figure S32. HRMS-DART^{+} (19 eV) of **3h**.

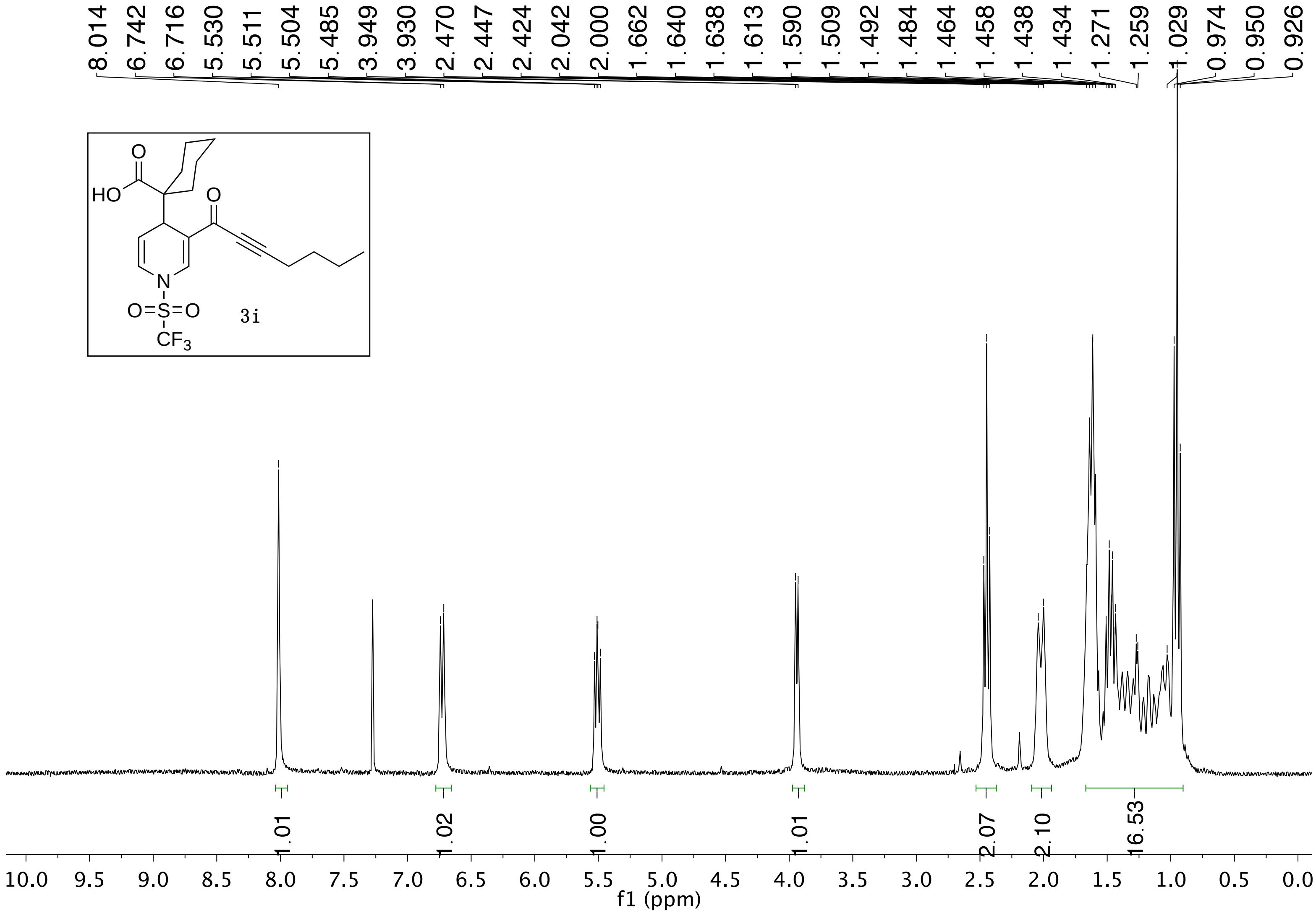

Figure S33. ^{1}H NMR (300 MHz/$CDCl_3$/TMS) of **3i**.

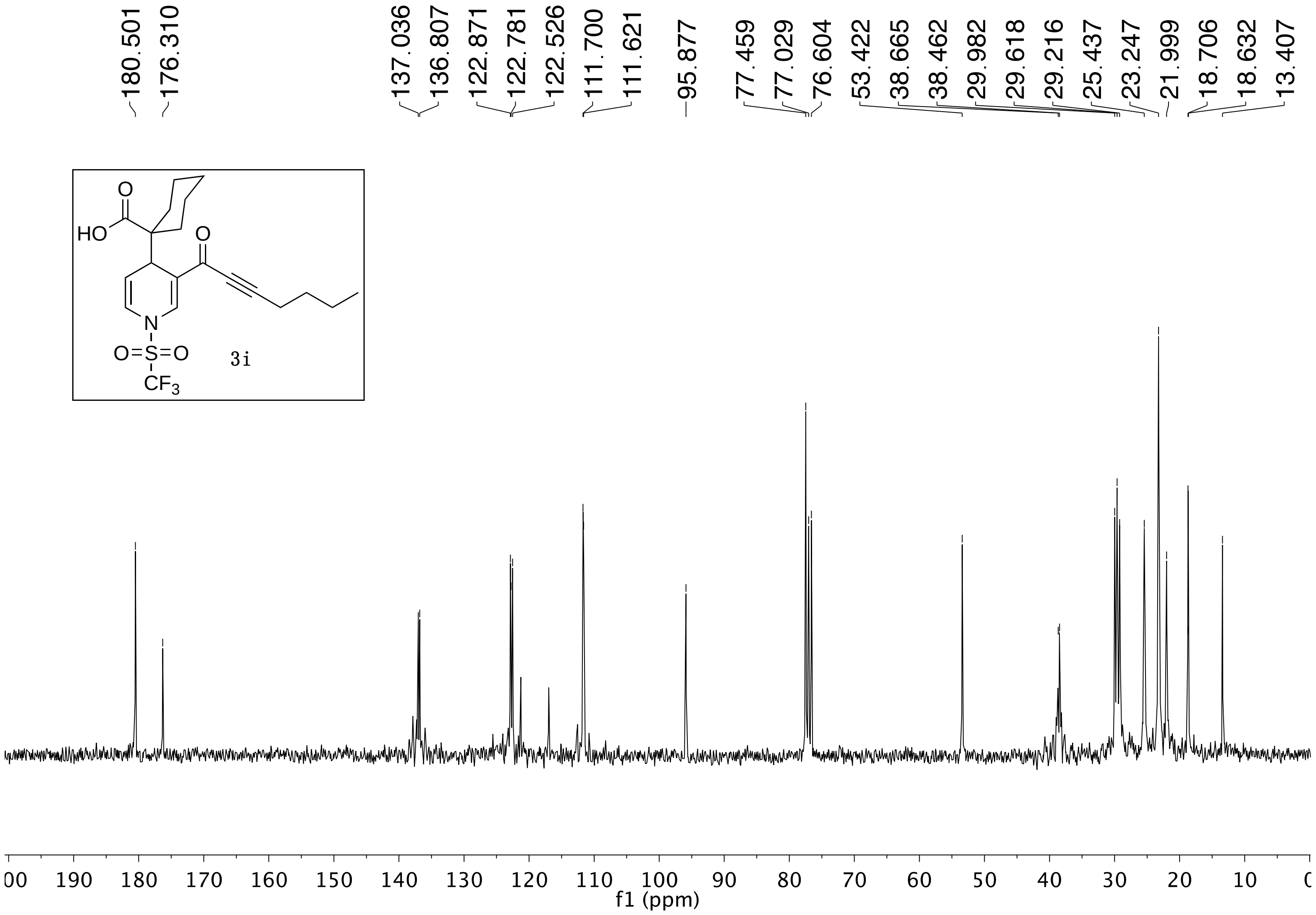

Figure S34. ^{13}C NMR (75 MHz/$CDCl_3$/TMS) of **3i**.

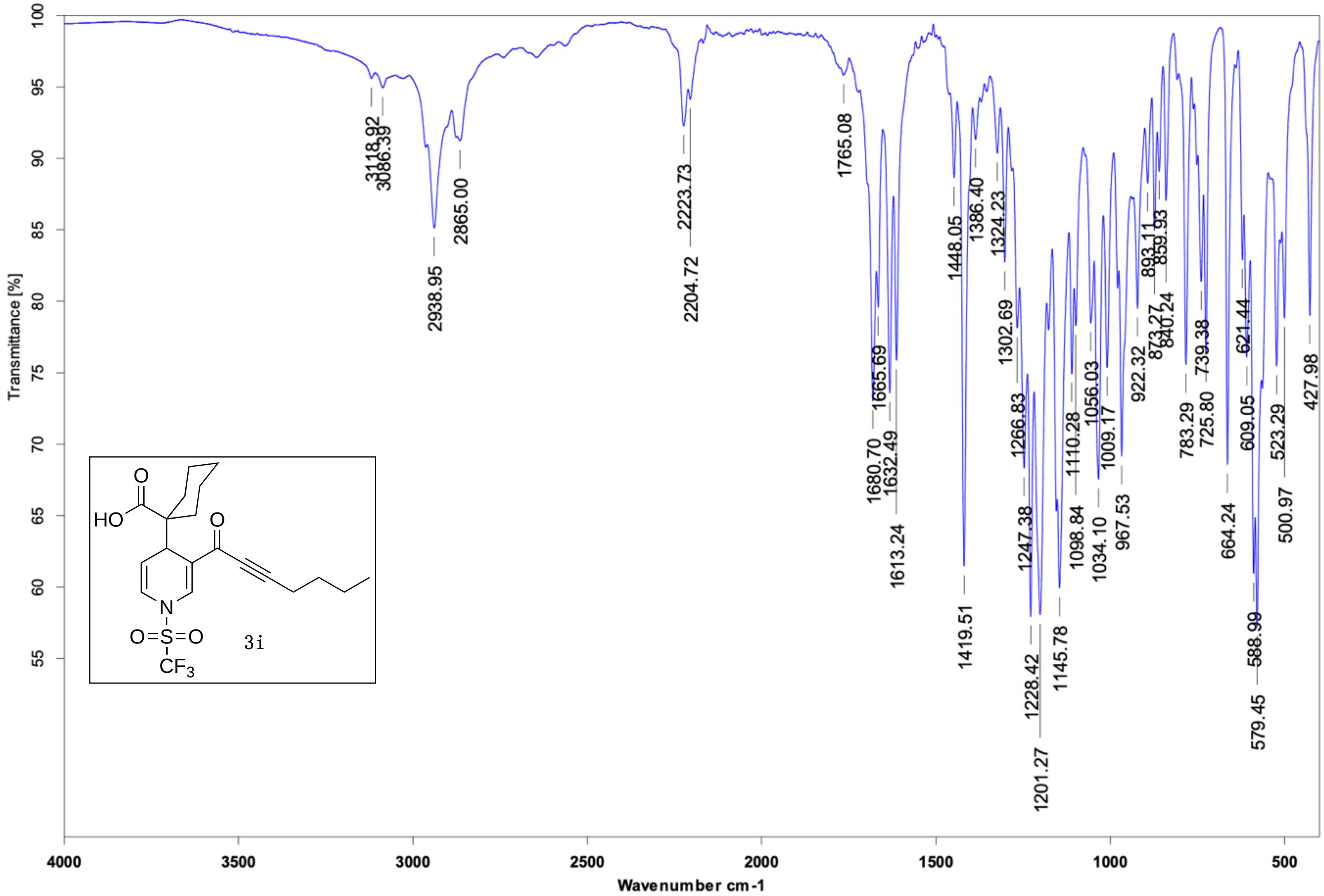

Figure S35. FTIR of **3i**.

Description:
Ionization Mode:ESI+
History:Determine m/z[Peak Detect[Centroid,30,Area];Correct Base[1.0%];Smooth[5]];Correct Base[5.0%];Average...

Mass Calibration data:Cal_Peg_600
Created:11/28/2018 2:32:19 PM
Created by:AccuTOF

Charge number:1
Tolerance:100.00(mmu)
Unsaturation Number:0.0 .. 50.0 (Fraction:Both)
Element:^{12}C:0 .. 20, ^{1}H:0 .. 25, ^{19}F:1 .. 3, ^{14}N:1 .. 1, ^{16}O:0 .. 5, ^{32}S:0 .. 1

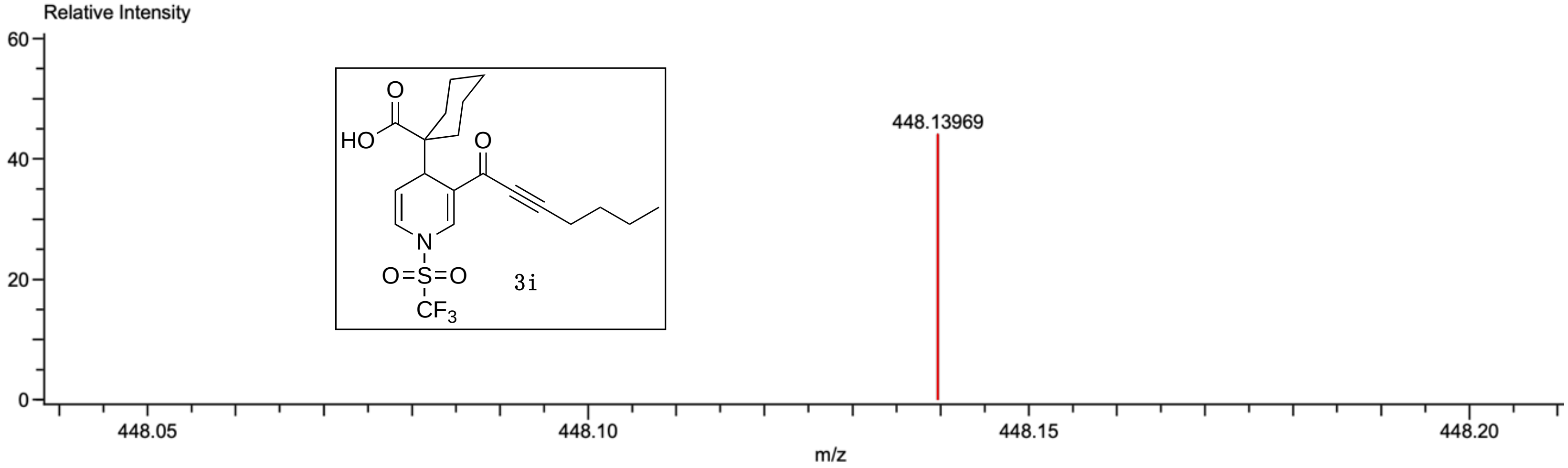

Mass	Intensity	Calc. Mass	Mass Difference (mmu)	Mass Difference (ppm)	Possible Formula	Unsaturation Number
448.13969	17150.00	448.14055	-0.86	-1.92	$^{12}C_{20}{}^{1}H_{25}{}^{19}F_{3}{}^{14}N_{1}{}^{16}O_{5}{}^{32}S_{1}$	8.5

Figure S36. HRMS-DART^{+} (19 eV) of **3i**.

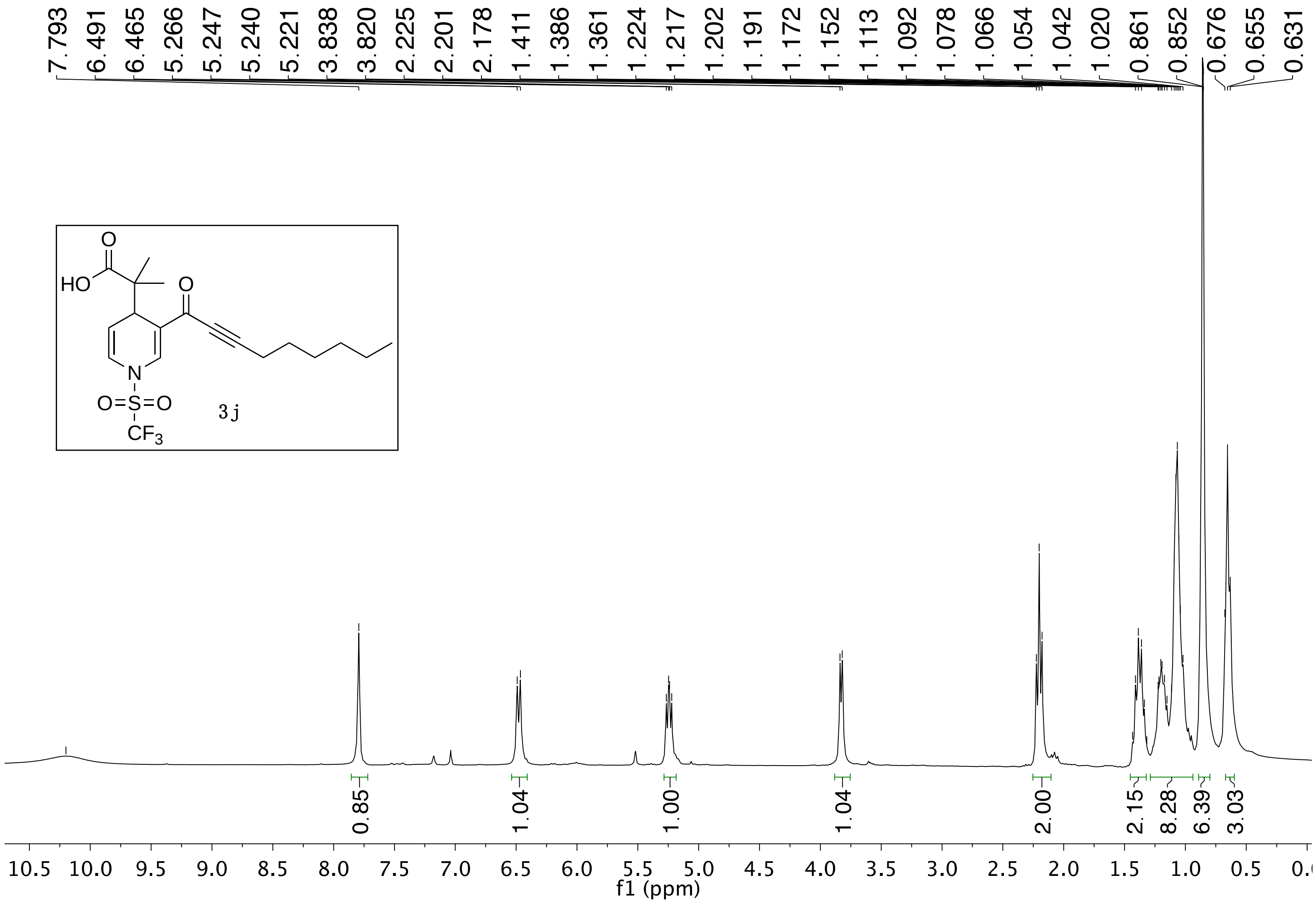

Figure S37. ^{1}H NMR (300 MHz/$CDCl_3$/TMS) of **3j**.

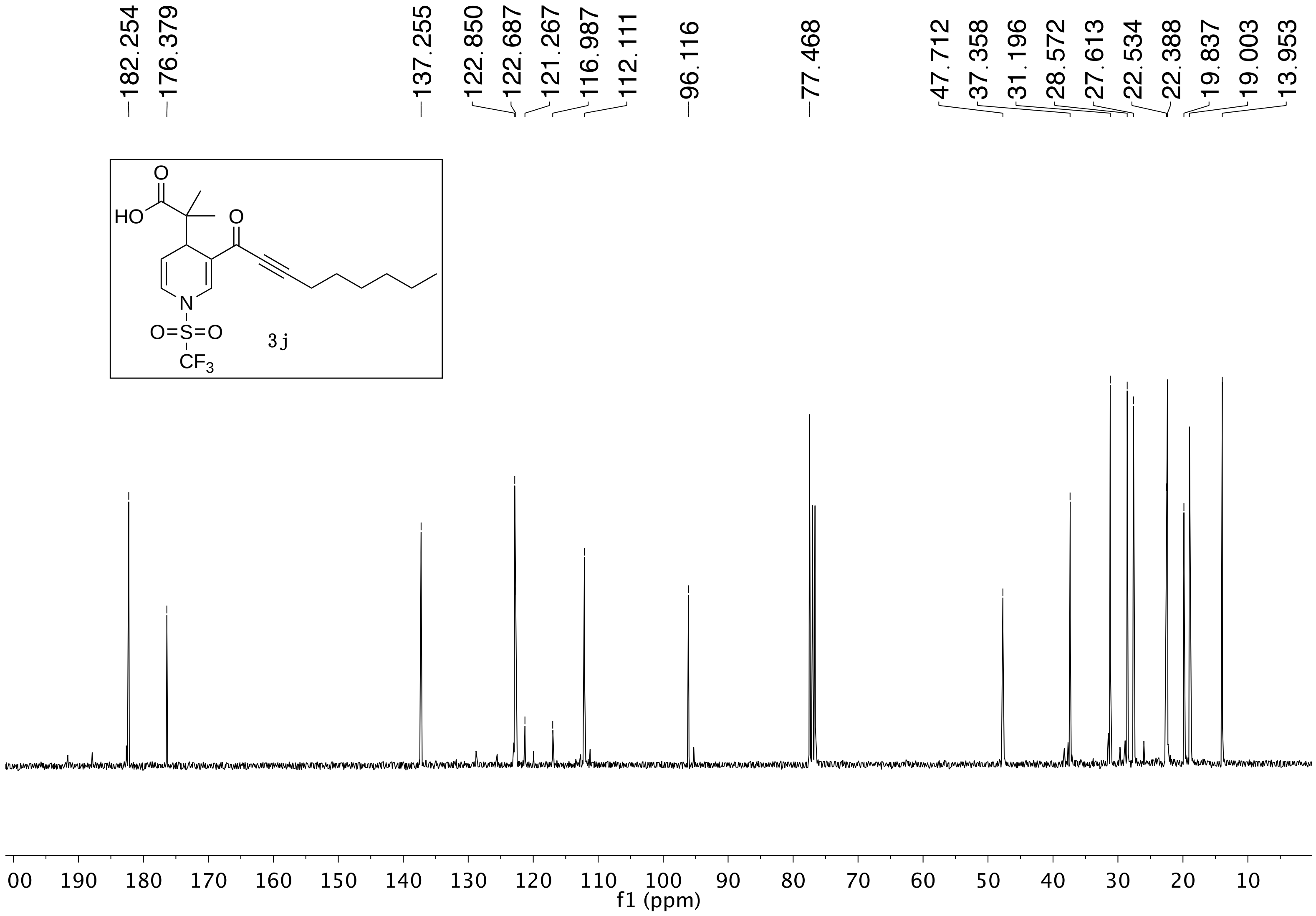

Figure S38. ^{13}C NMR (75 MHz/$CDCl_3$/TMS) of **3j**.

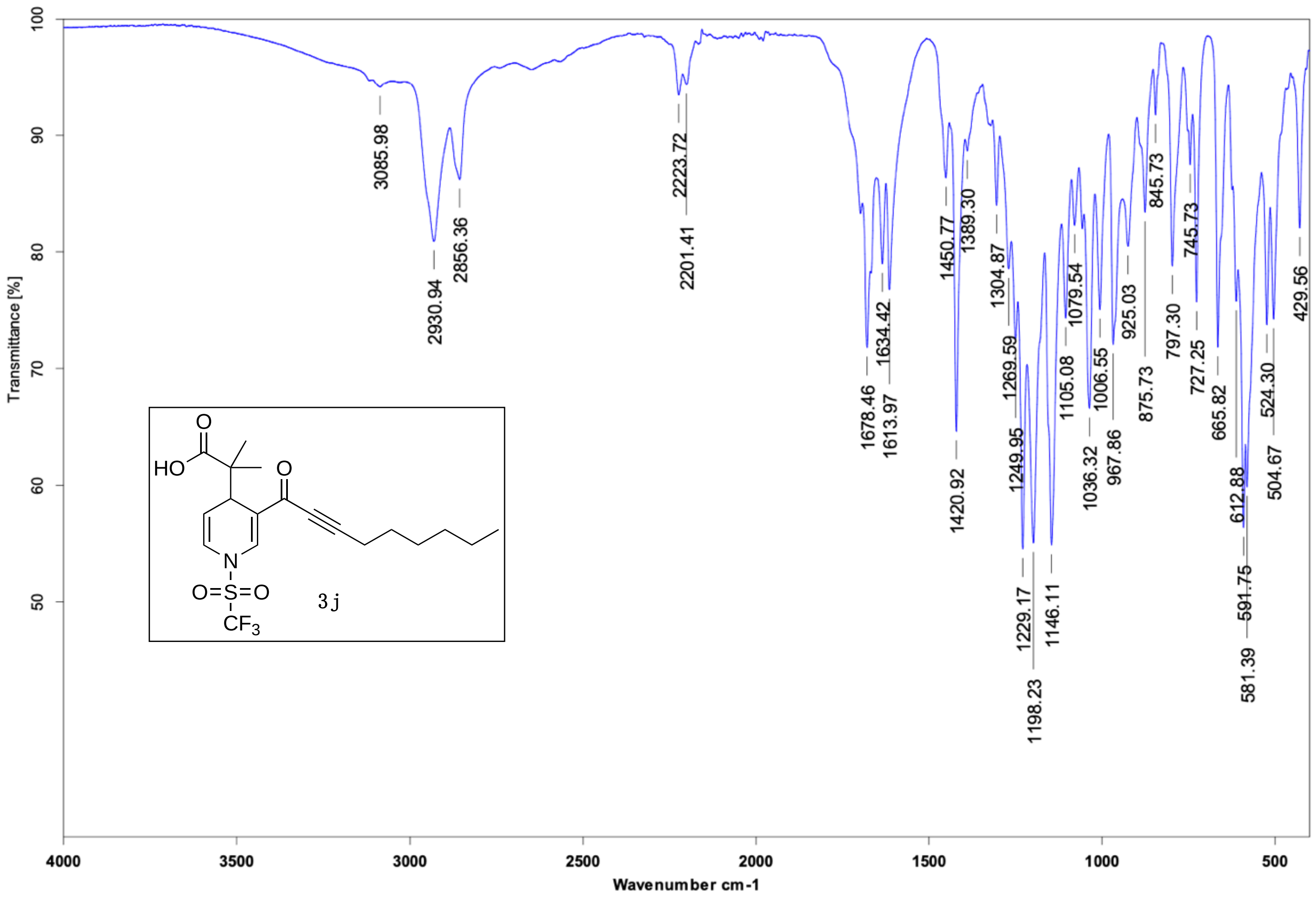

Figure S39. FTIR of **3j**.

Description:
Ionization Mode:ESI+
History:Determine m/z[Peak Detect[Centroid,30,Area];Correct Base[1.0%];Smooth[5]];Correct Base[5.0%];Average...

Mass Calibration data:Cal_Peg_600
Created:11/28/2018 2:34:33 PM
Created by:AccuTOF

Charge number:1
Tolerance:100.00(mmu)
Unsaturation Number:0.0 .. 50.0 (Fraction:Both)
Element:^{12}C:0 .. 19, ^{1}H:0 .. 25, ^{19}F:1 .. 3, ^{14}N:1 .. 1, ^{16}O:0 .. 5, ^{32}S:0 .. 1

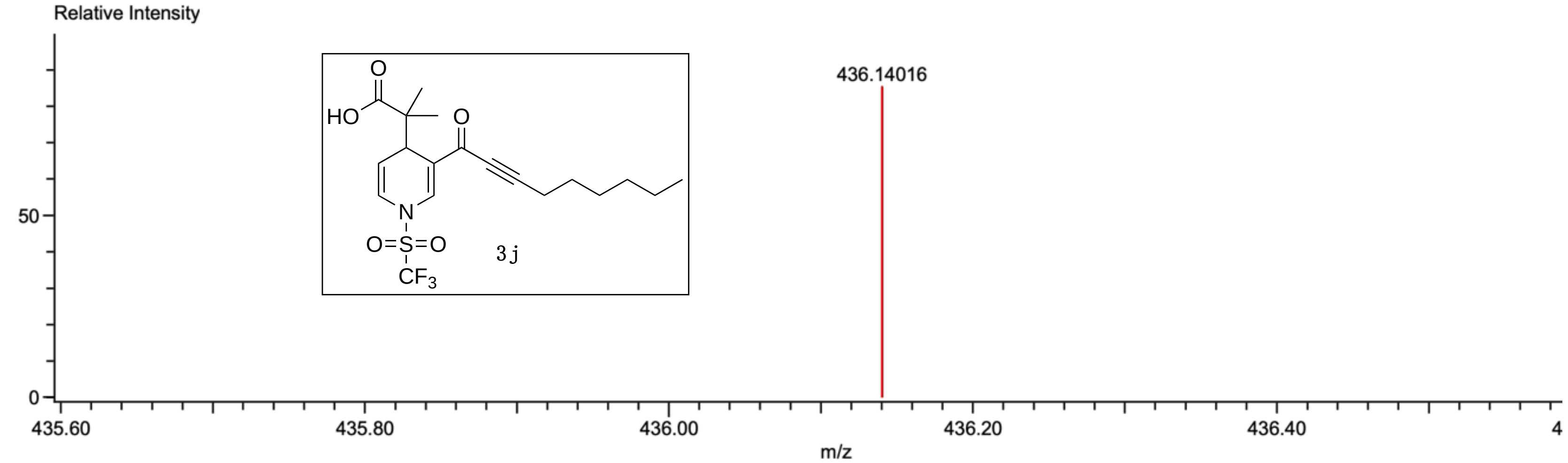

Mass	Intensity	Calc. Mass	Mass Difference (mmu)	Mass Difference (ppm)	Possible Formula	Unsaturation Number
436.14016	340435.65	436.14055	-0.39	-0.89	$^{12}C_{19}{}^{1}H_{25}{}^{19}F_{3}{}^{14}N_{1}{}^{16}O_{5}{}^{32}S_{1}$	7.5

Figure S40. HRMS-DART^{+} (19 eV) of **3j**.

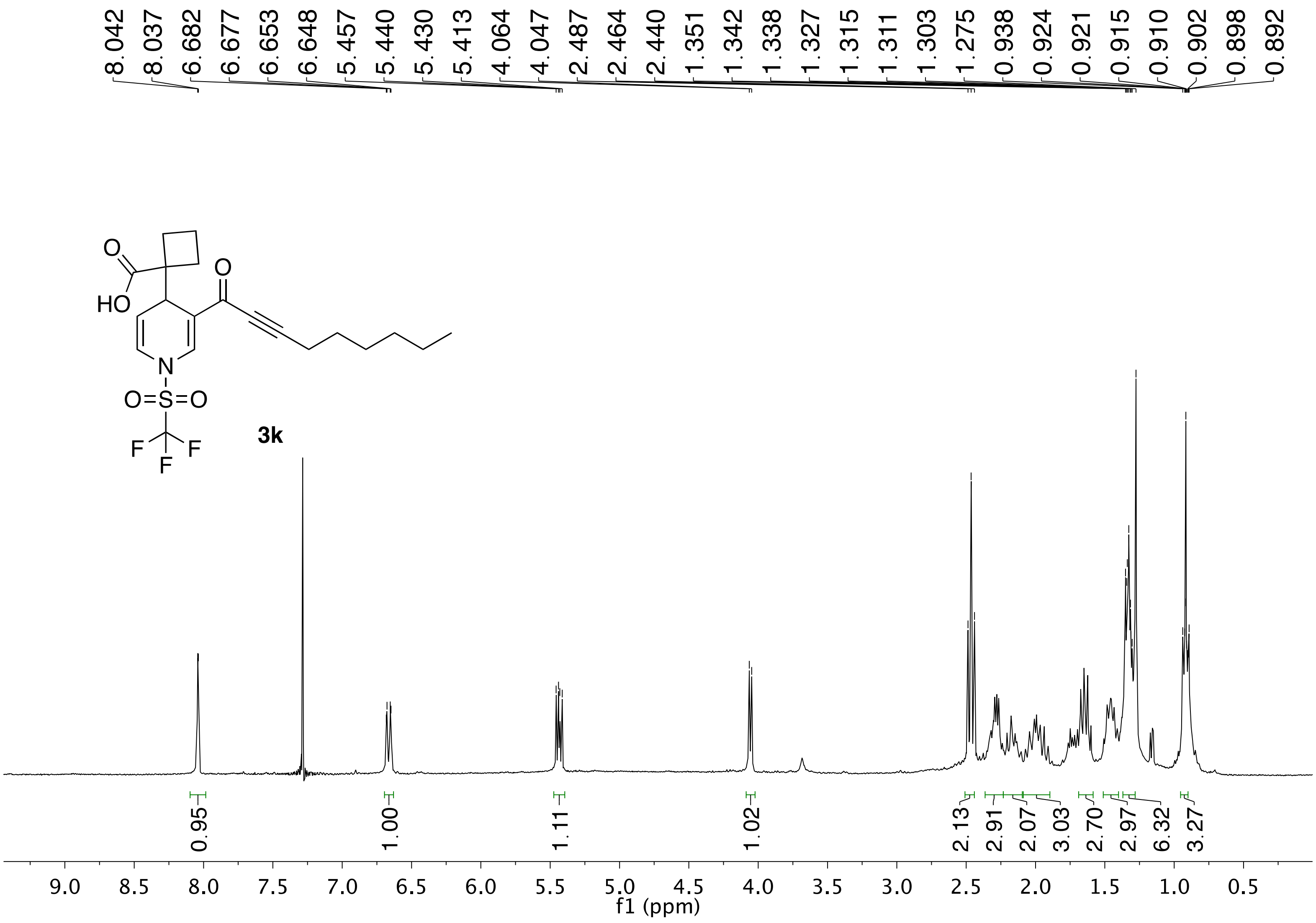

Figure S41. ^{1}H NMR (300 MHz/$CDCl_3$/TMS) of **3k**.

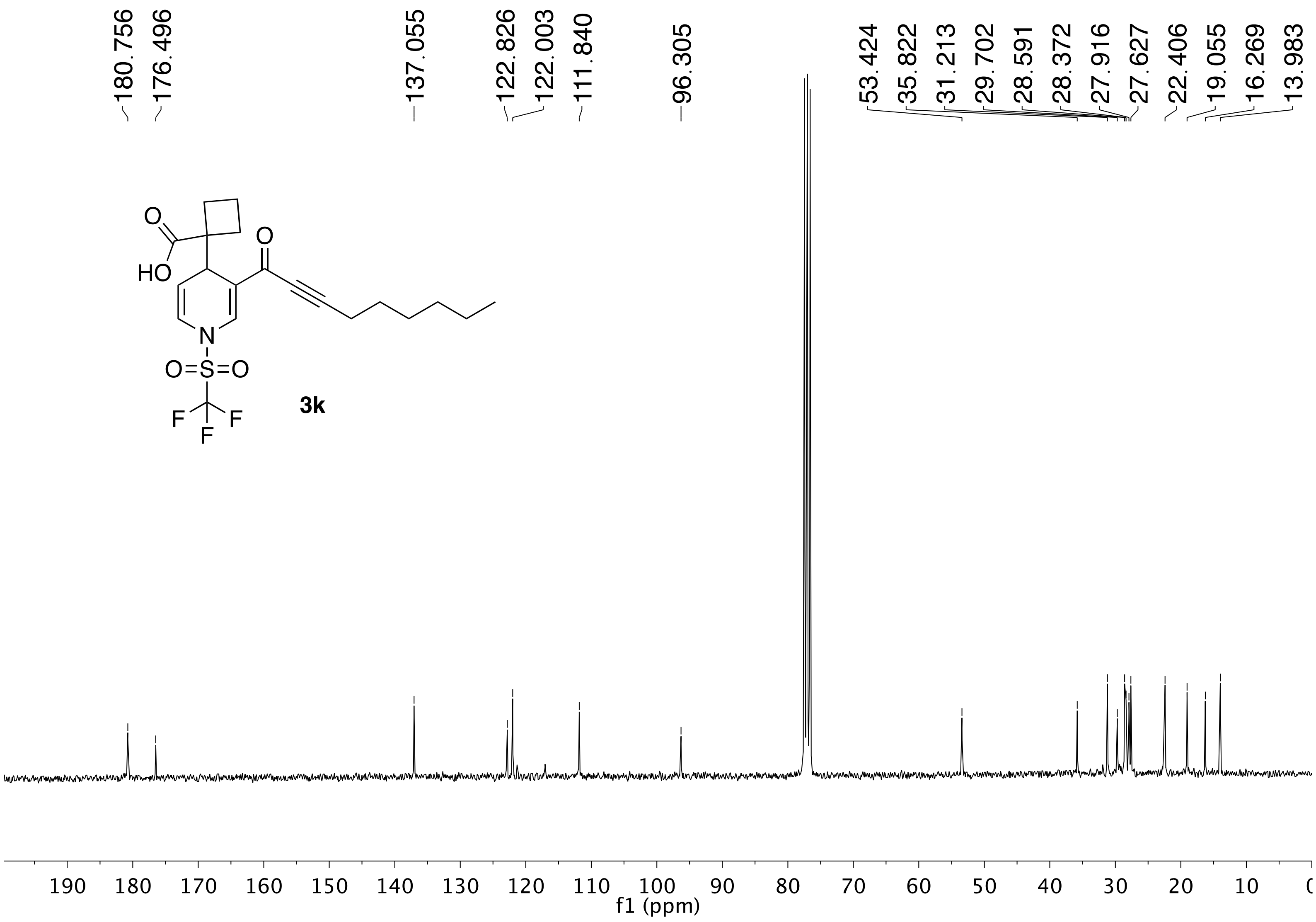

Figure S42. ^{13}C NMR (75 MHz/$CDCl_3$/TMS) of **3k**.

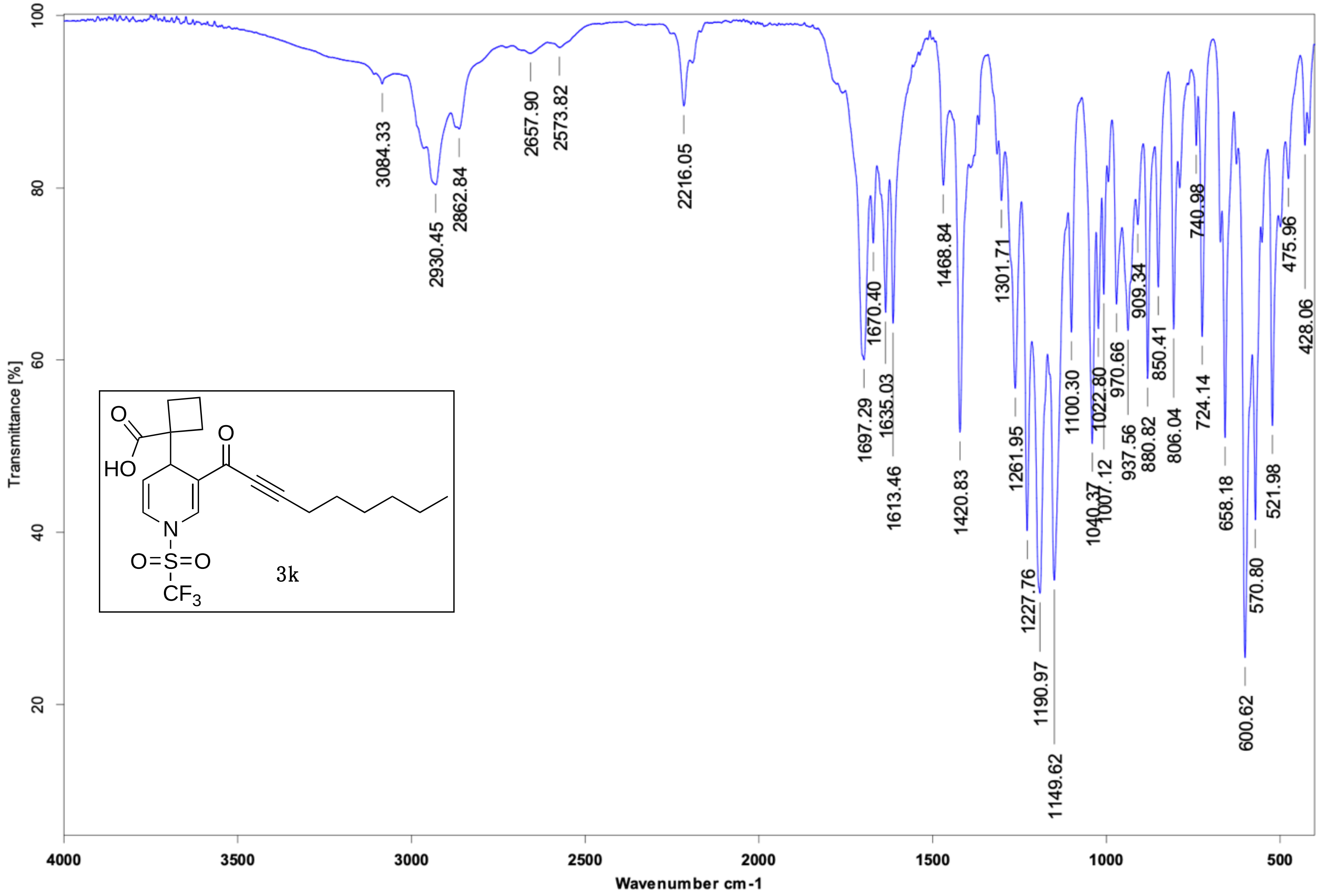

Figure S43. FTIR of **3k**.

Description:
Ionization Mode:ESI+
History:Determine m/z[Peak Detect[Centroid,30,Area];Correct Base[1.0%];Smooth[5]];Correct Base[5.0%];Average...

Mass Calibration data:Cal_Peg_600
Created:11/28/2018 2:36:20 PM
Created by:AccuTOF

Charge number:1
Tolerance:100.00(mmu)
Unsaturation Number:0.0 .. 50.0 (Fraction:Both)
Element:^{12}C:0 .. 20, ^{1}H:0 .. 25, ^{19}F:1 .. 3, ^{14}N:1 .. 1, ^{16}O:0 .. 5, ^{32}S:0 .. 1

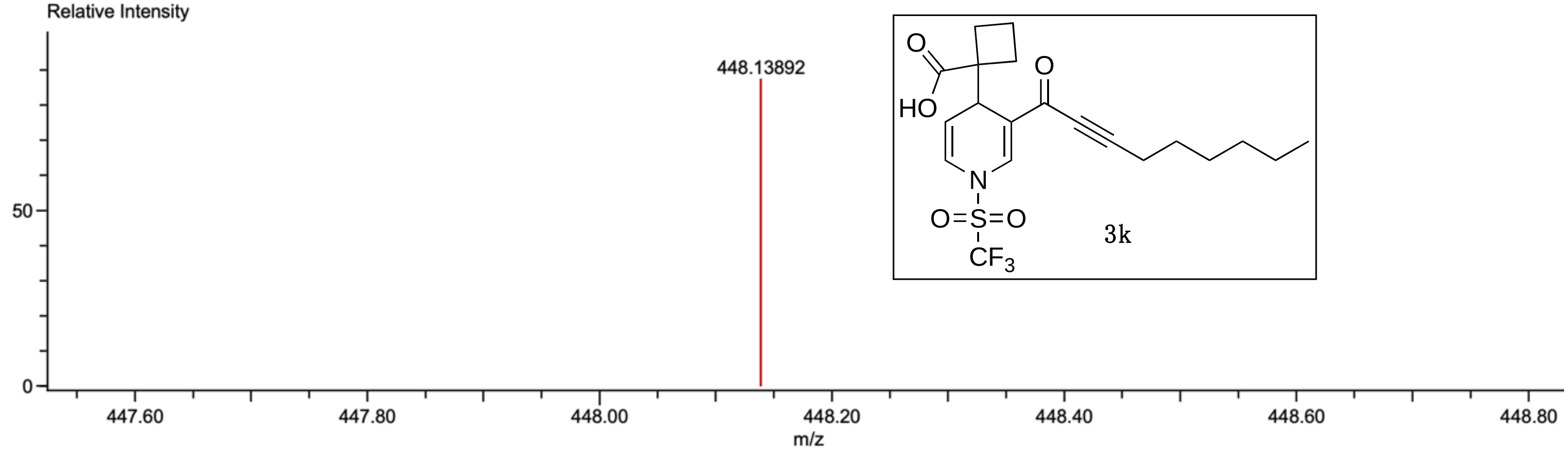

Mass	Intensity	Calc. Mass	Mass Difference (mmu)	Mass Difference (ppm)	Possible Formula	Unsaturation Number
448.13892	794418.42	448.14055	-1.63	-3.64	$^{12}C_{20}{}^{1}H_{25}{}^{19}F_{3}{}^{14}N_{1}{}^{16}O_{5}{}^{32}S_{1}$	8.5

Figure S44. HRMS-DART^{+} (19 eV) of **3k**.

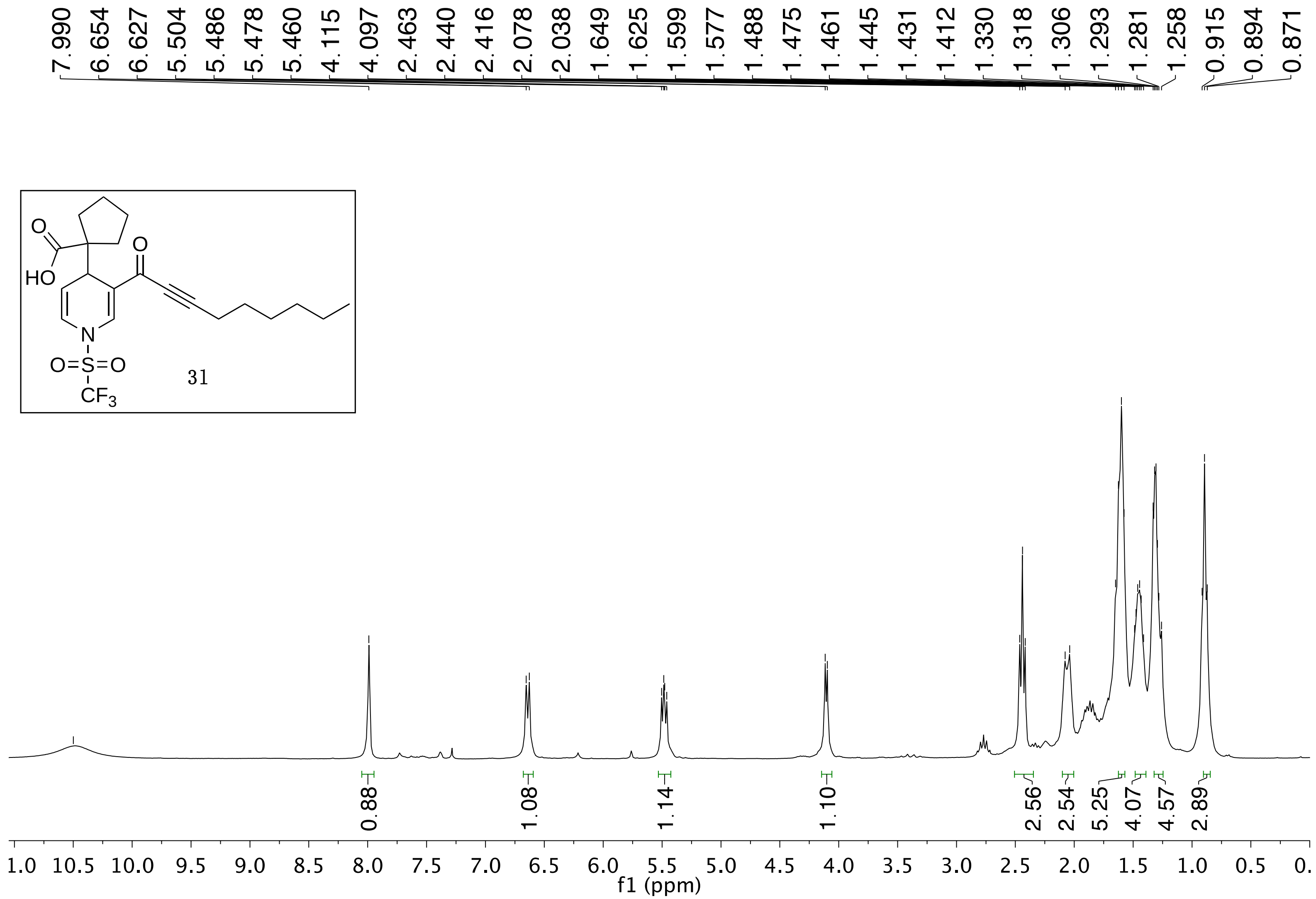

Figure S45. ^{1}H NMR (300 MHz/CDCl$_3$/TMS) of **3l**.

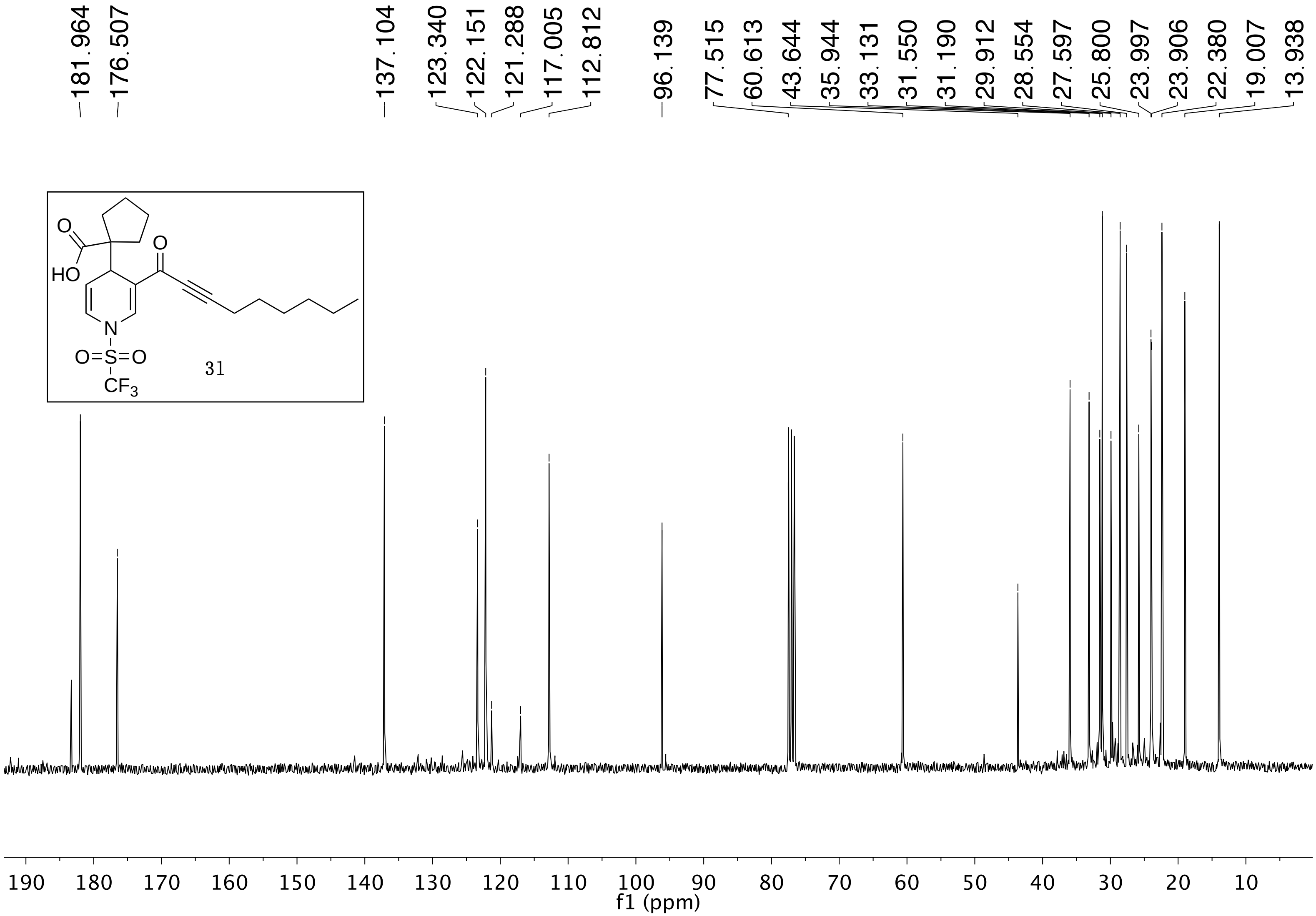

Figure S46. ^{13}C NMR (75 MHz/$CDCl_3$/TMS) of **3l**.

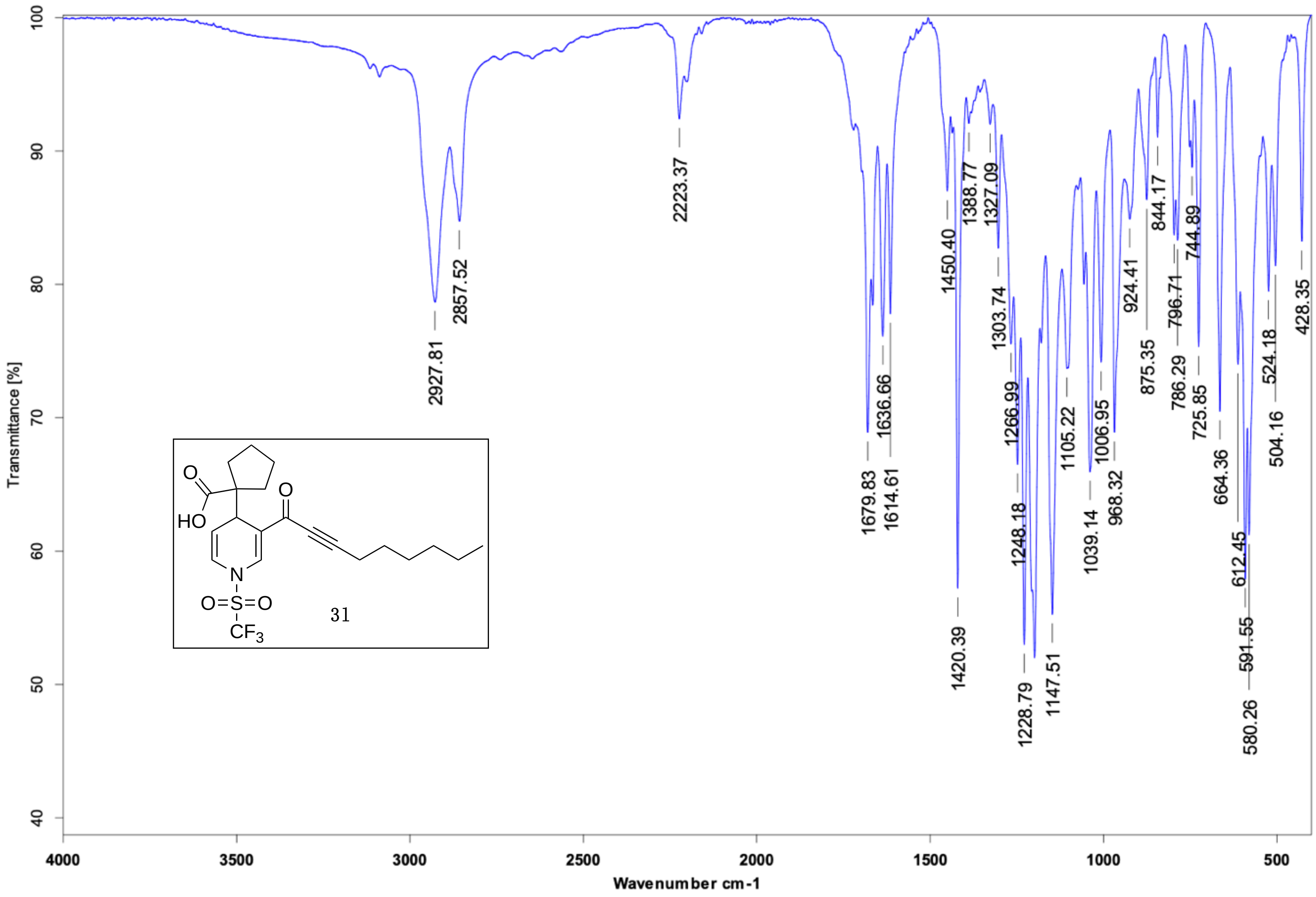

Figure S47. FTIR of **3l**.

Description:
Ionization Mode:ESI+
History:Determine m/z[Peak Detect[Centroid,30,Area];Correct Base[];Smooth[5]];Correct Base[5.0%];Average(MS[...

Mass Calibration data:Cal_PEG_600
Created:10/6/2022 11:44:12 AM
Created by:AccuTOF

Charge number:1 Tolerance:5.00(mmu)
Element:^{12}C:0 .. 22, ^{1}H:0 .. 30, ^{19}F:0 .. 3, ^{14}N:0 .. 1, ^{16}O:1 .. 6, ^{32}S:1 .. 1

Unsaturation Number:0.0 .. 50.0 (Fraction:Both)

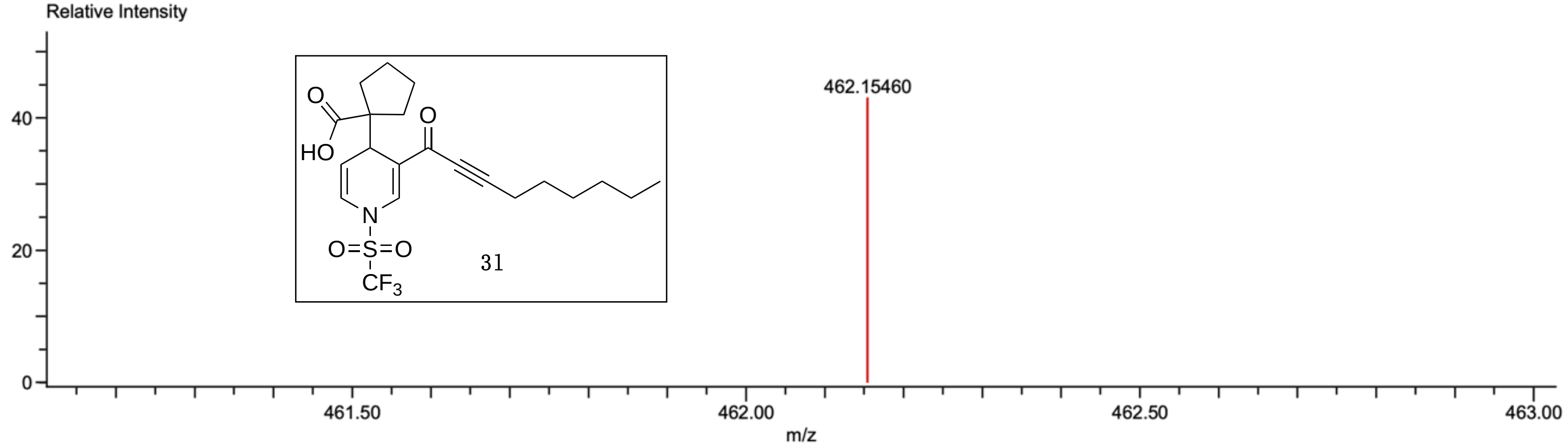

Mass	Intensity	Calc. Mass	Mass Difference (mmu)	Mass Difference (ppm)	Possible Formula	Unsaturation Number
462.15460	69692.22	462.15620	-1.60	-3.46	$^{12}C_{21}{}^{1}H_{27}{}^{19}F_{3}{}^{14}N_{1}{}^{16}O_{5}{}^{32}S_{1}$	8.5

Figure S48. HRMS-DART^{+} (19 eV) of **3l**.

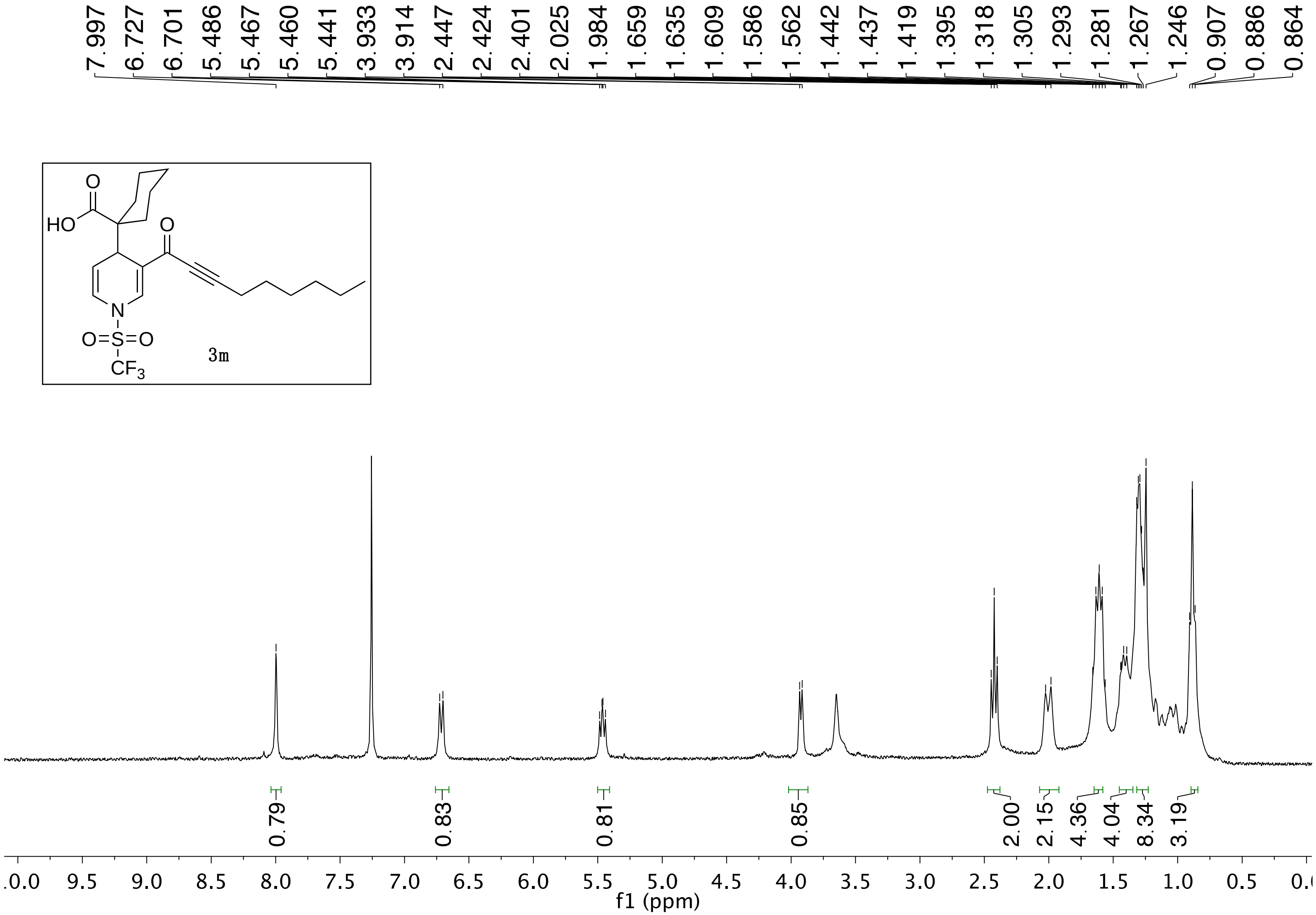

Figure S49. ^{1}H NMR (300 MHz/$CDCl_3$/TMS) of **3m**.

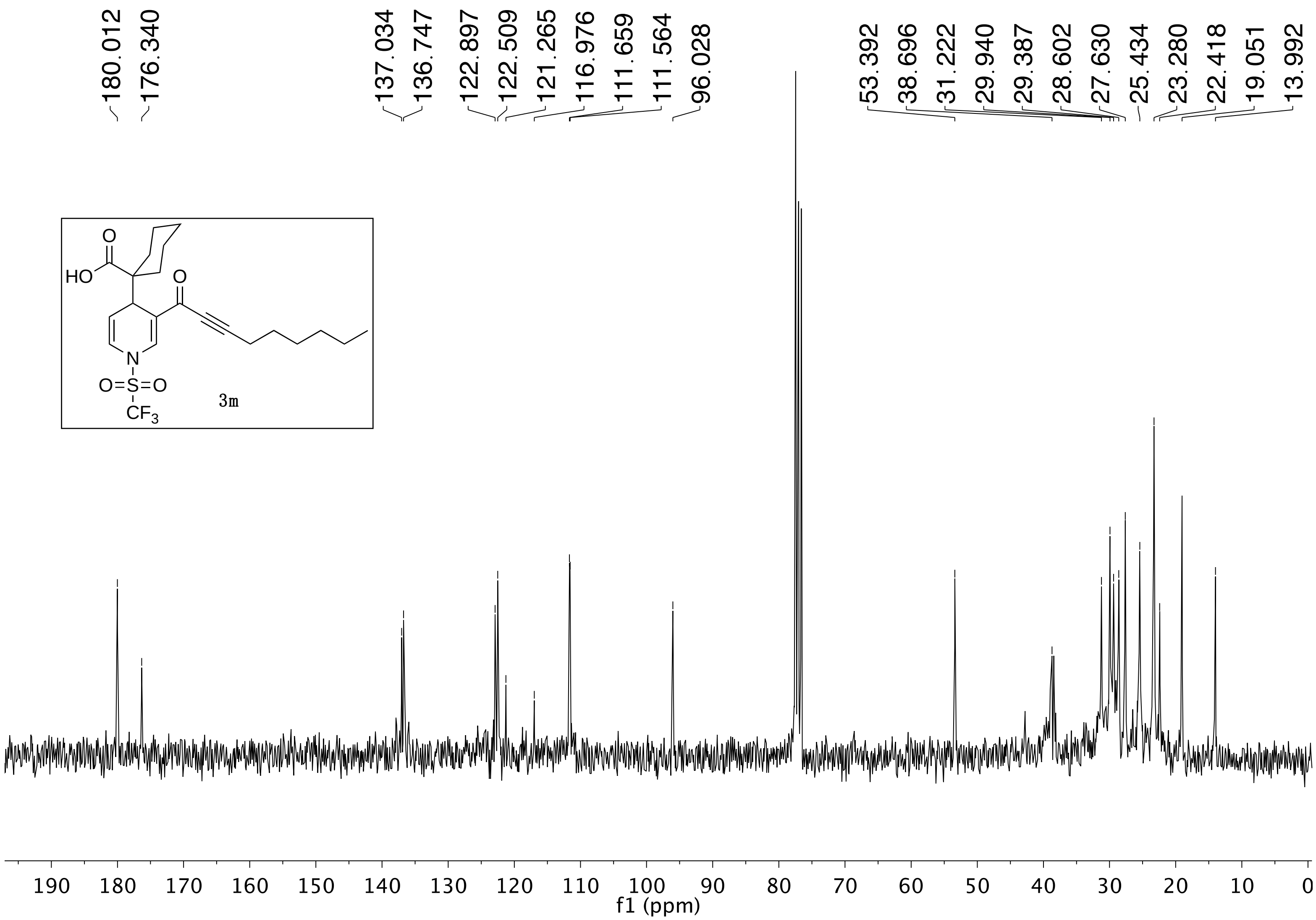

Figure S50. ^{13}C NMR (75 MHz/$CDCl_3$/TMS) of **3m**.

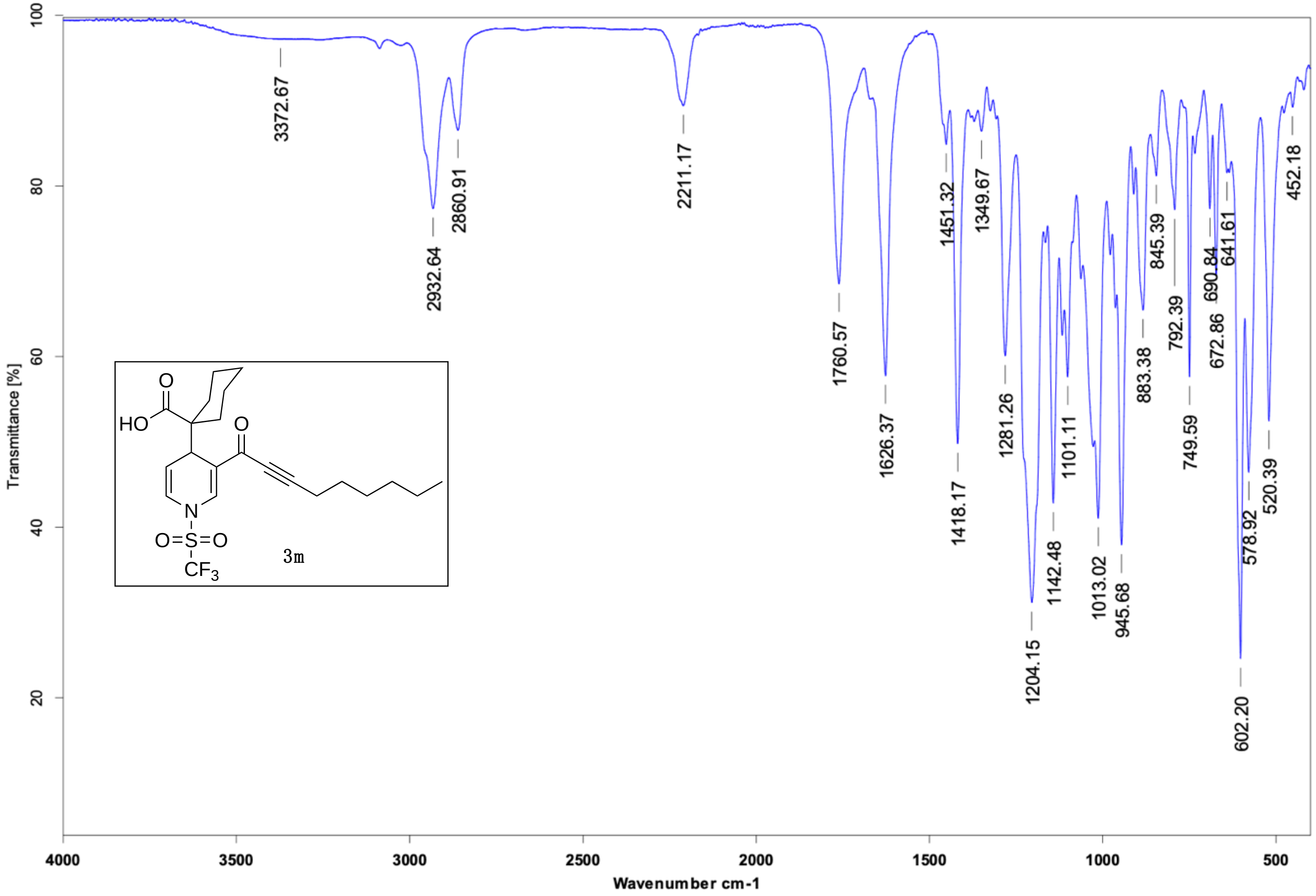

Figure S51. FTIR of **3m**.

Description:
Ionization Mode:ESI+
History:Determine m/z[Peak Detect[Centroid,30,Area];Correct Base[1.0%];Smooth[5]];Correct Base[5.0%];Average...

Mass Calibration data:Cal_Peg_600
Created:11/28/2018 2:42:05 PM
Created by:AccuTOF

Charge number:1
Tolerance:100.00(mmu)
Unsaturation Number:0.0 .. 50.0 (Fraction:Both)
Element:^{12}C:0 .. 22, ^{1}H:0 .. 29, ^{19}F:1 .. 3, ^{14}N:1 .. 1, ^{16}O:0 .. 5, ^{32}S:0 .. 1

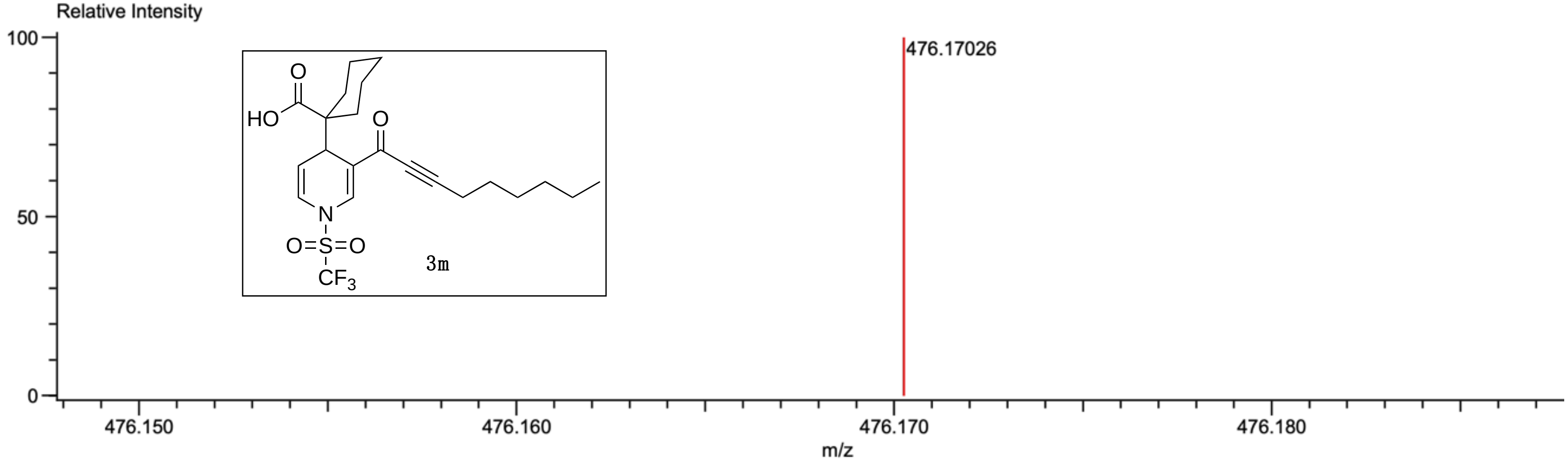

Mass	Intensity	Calc. Mass	Mass Difference (mmu)	Mass Difference (ppm)	Possible Formula	Unsaturation Number
476.17026	131468.93	476.17185	-1.59	-3.34	$^{12}C_{22}{}^{1}H_{29}{}^{19}F_{3}{}^{14}N_{1}{}^{16}O_{5}{}^{32}S_{1}$	8.5

Figure S52. HRMS-DART+ (19 eV) of **3m**.

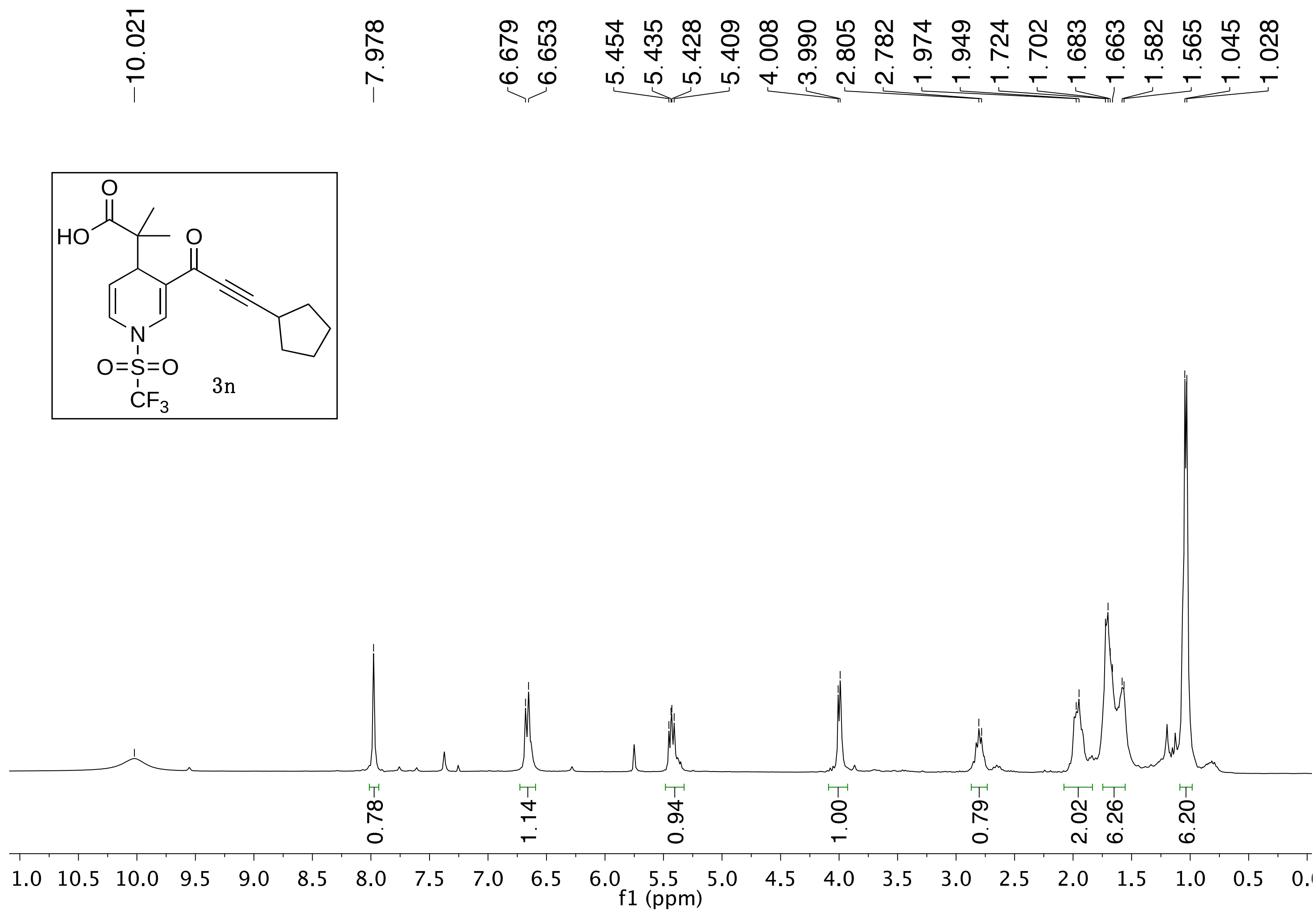

Figure S53. ^{1}H NMR (300 MHz/$CDCl_3$/TMS) of **3n**.

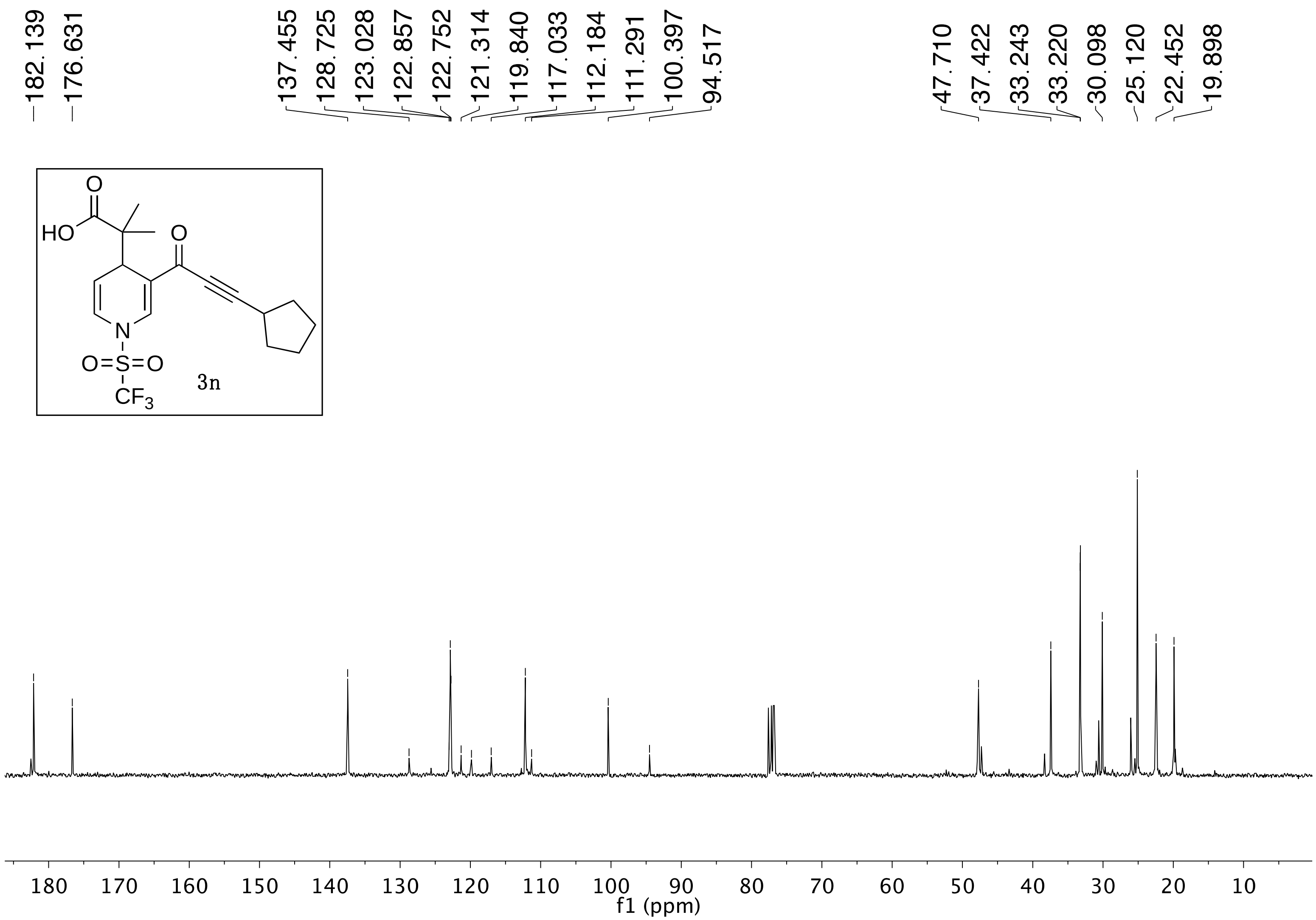

Figure S54. ^{13}C NMR (75 MHz/$CDCl_3$/TMS) of **3n**.

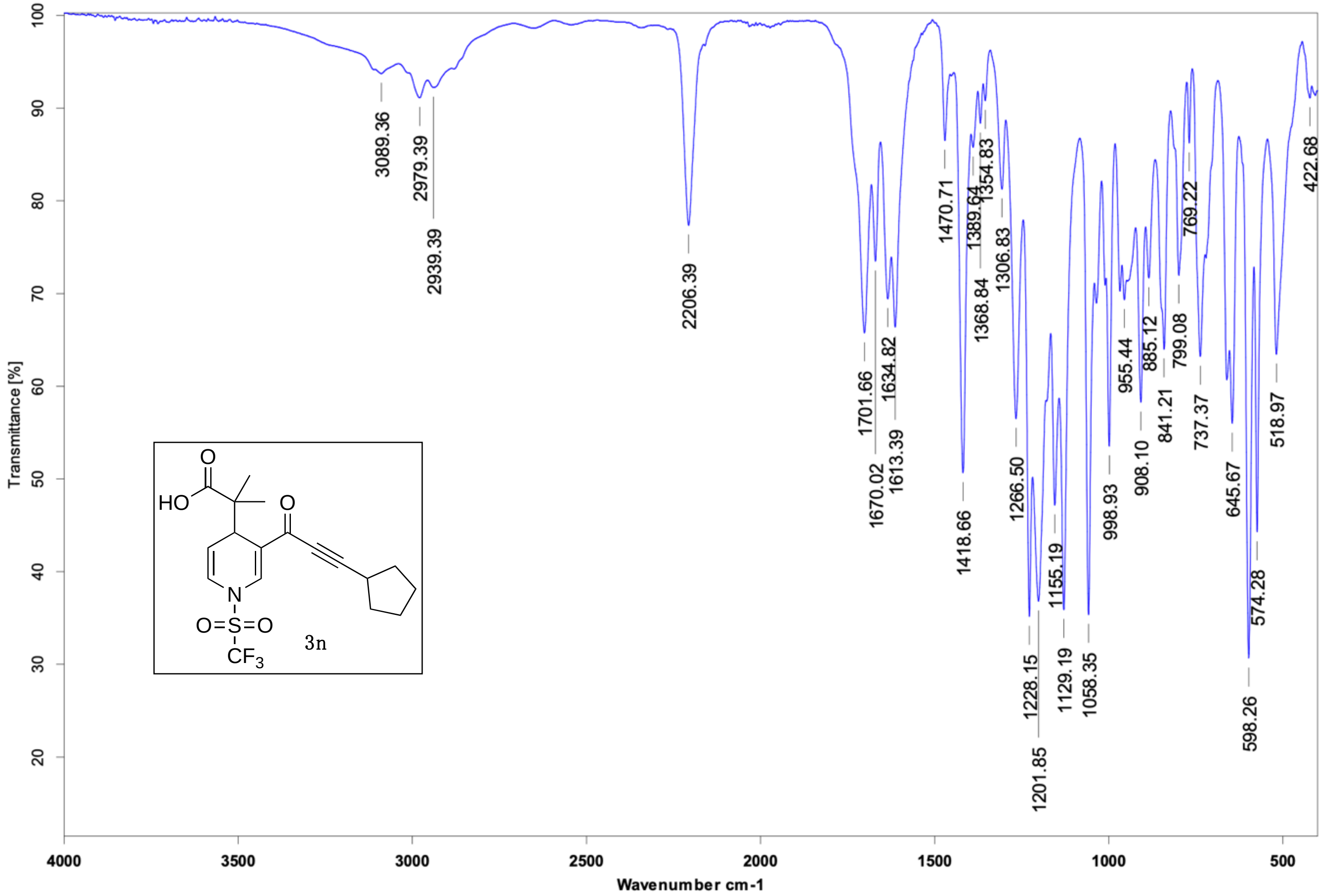

Figure S55. FTIR of **3n**.

Description:
Ionization Mode:ESI+
History:Determine m/z[Peak Detect[Centroid,30,Area];Correct Base[];Smooth[5]];Correct Base[5.0%];Average(MS[...

Mass Calibration data:Cal_PEG_600
Created:10/6/2022 11:41:18 AM
Created by:AccuTOF

Charge number:1 Tolerance:5.00(mmu) Unsaturation Number:0.0 .. 50.0 (Fraction:Both)
Element:^{12}C:0 .. 20, ^{1}H:0 .. 22, ^{19}F:0 .. 3, ^{14}N:0 .. 1, ^{16}O:1 .. 6, ^{32}S:1 .. 1

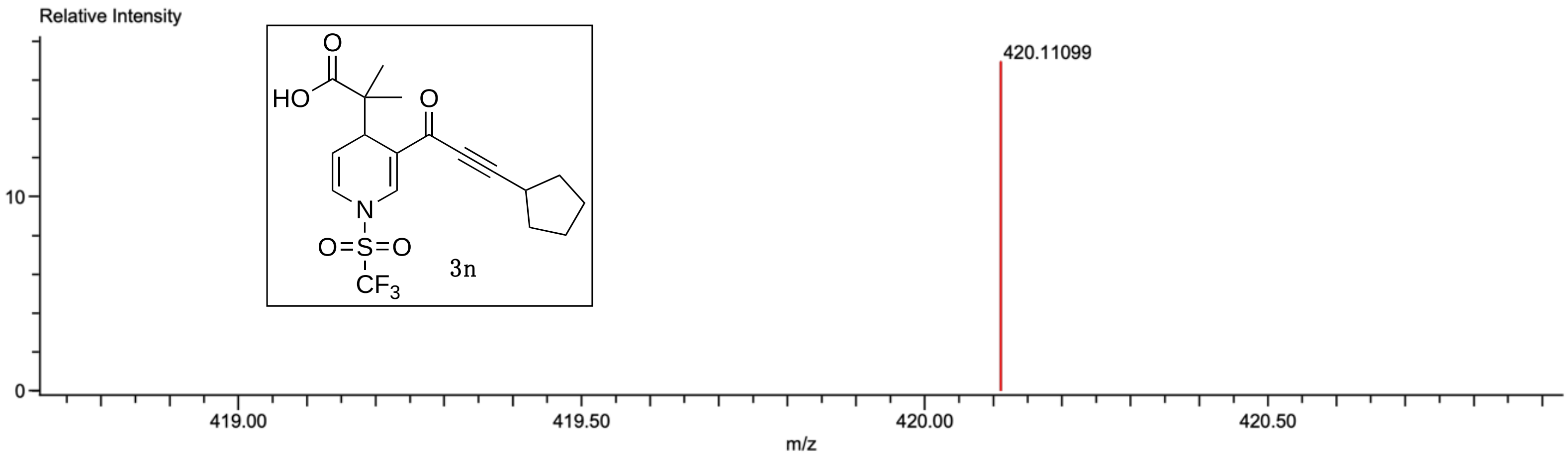

Mass	Intensity	Calc. Mass	Mass Difference (mmu)	Mass Difference (ppm)	Possible Formula	Unsaturation Number
420.11099	36713.63	420.10925	1.73	4.13	$^{12}C_{18}{}^{1}H_{21}{}^{19}F_{3}{}^{14}N_{1}{}^{16}O_{5}{}^{32}S_{1}$	8.5

Figure S56. HRMS-DART^{+} (19 eV) of **3n**.

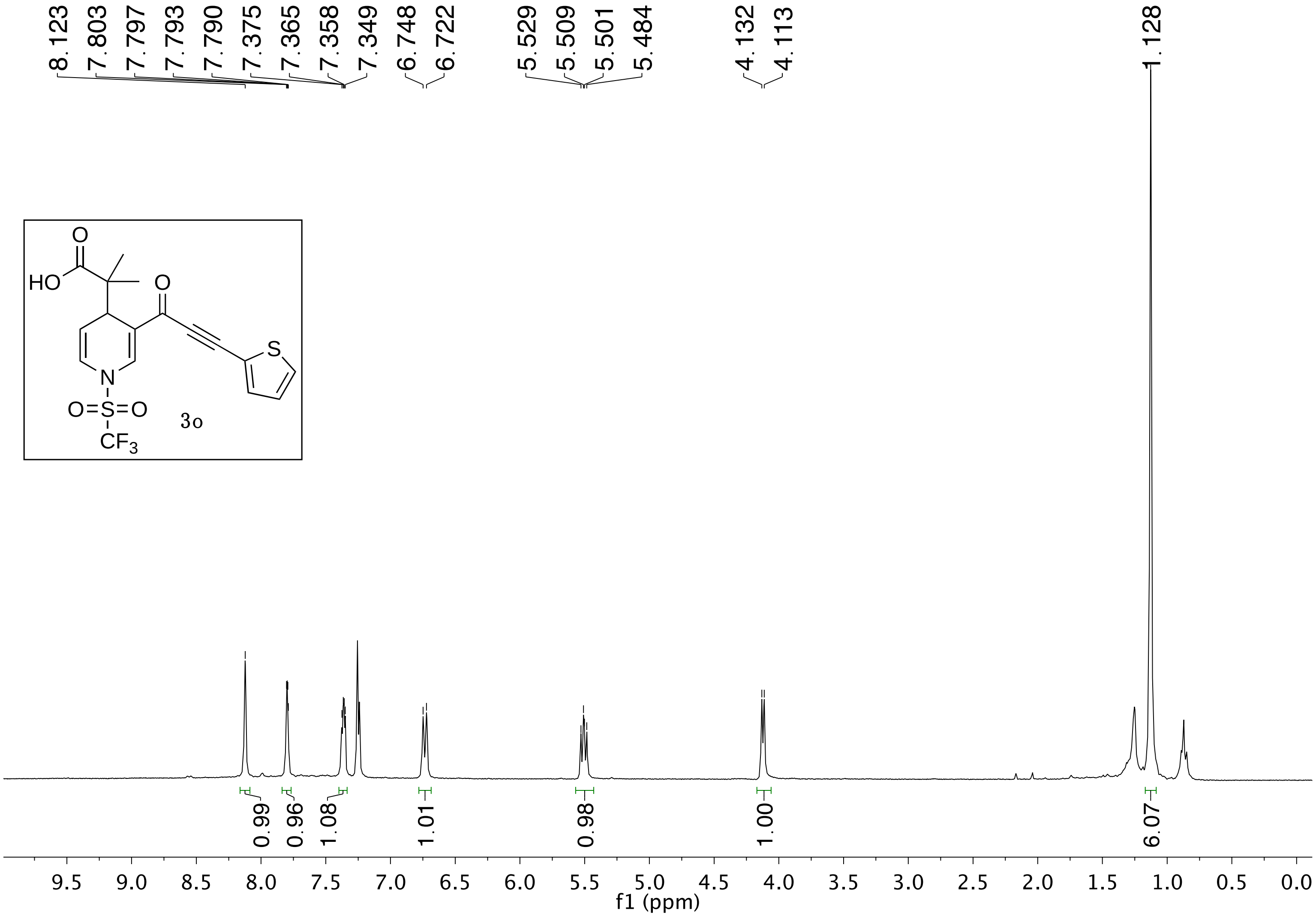

Figure S57. ^{1}H NMR (300 MHz/$CDCl_3$/TMS) of **3o**.

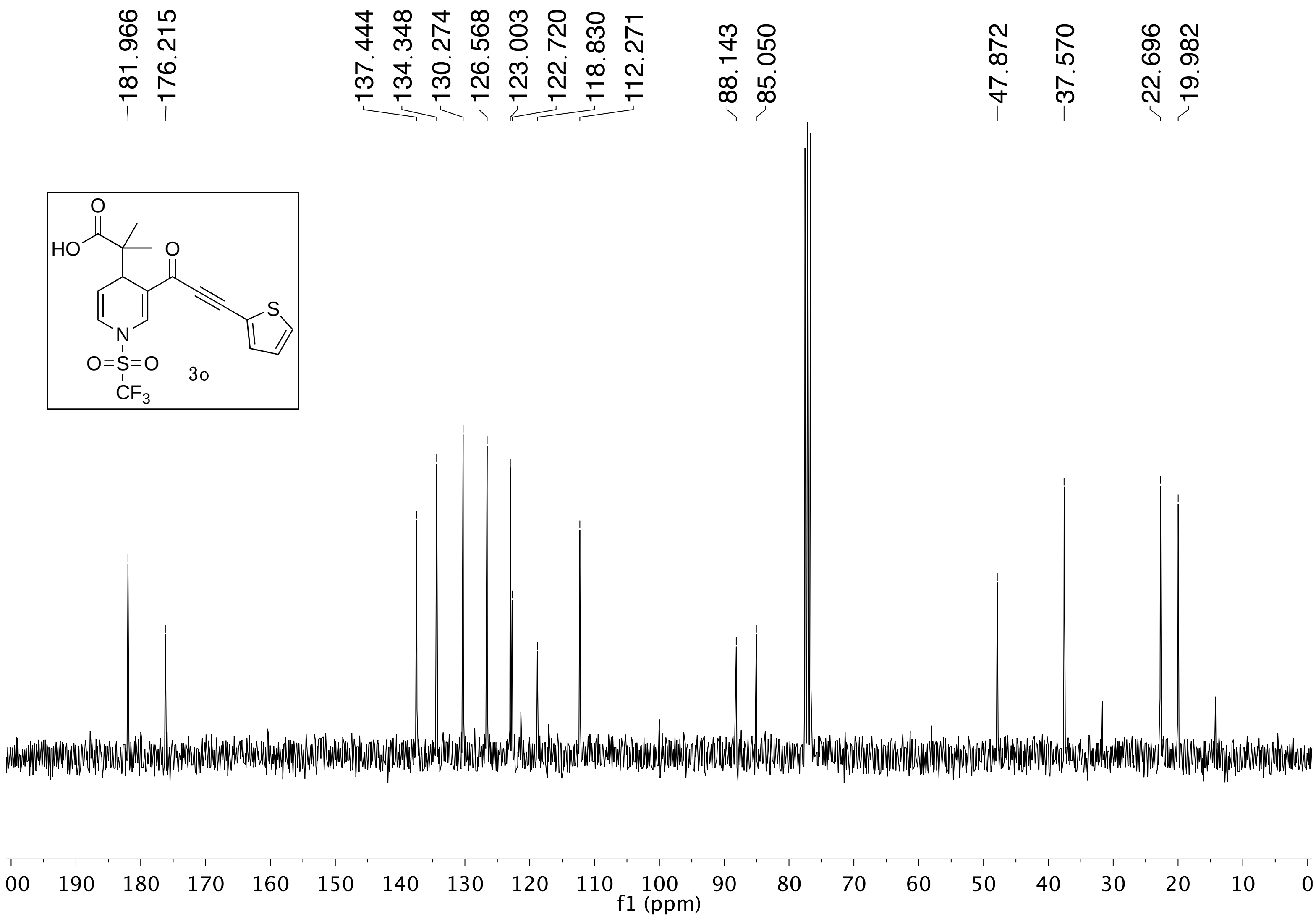

Figure S58. ^{13}C NMR (75 MHz/$CDCl_3$/TMS) of **3o**.

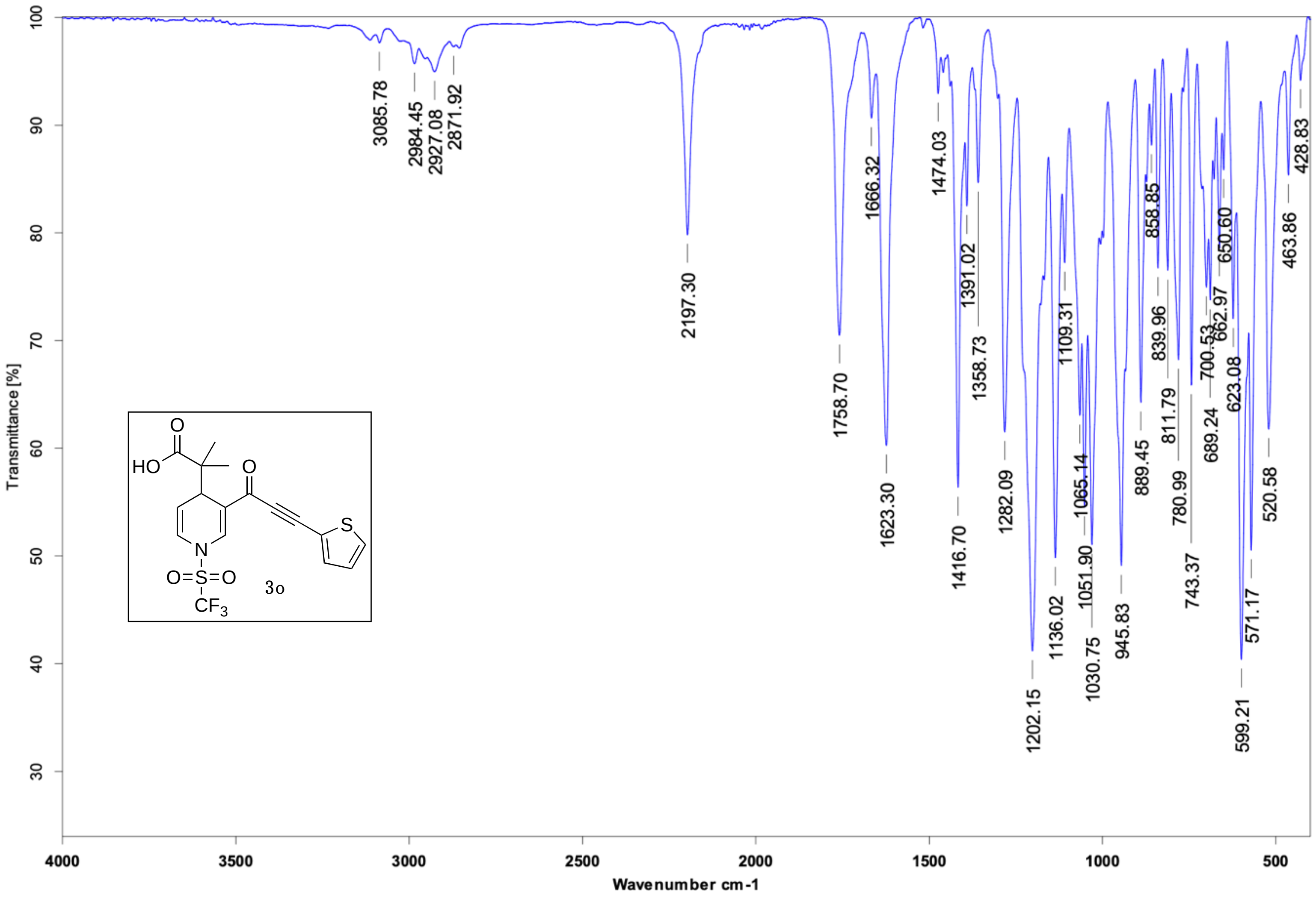

Figure S59. FTIR of **3o**.

Description:
Ionization Mode:ESI+
History:Determine m/z[Peak Detect[Centroid,30,Area];Correct Base[1.0%];Smooth[5]];Correct Base[5.0%];Average...

Mass Calibration data:Cal_Peg_600
Created:11/22/2018 1:42:46 PM
Created by:AccuTOF

Charge number:1
Tolerance:3.00(mmu)
Unsaturation Number:0.0 .. 50.0 (Fraction:Both)
Element:^{12}C:0 .. 17, ^{1}H:0 .. 15, ^{19}F:0 .. 3, ^{14}N:0 .. 1, ^{16}O:0 .. 5, ^{32}S:0 .. 2

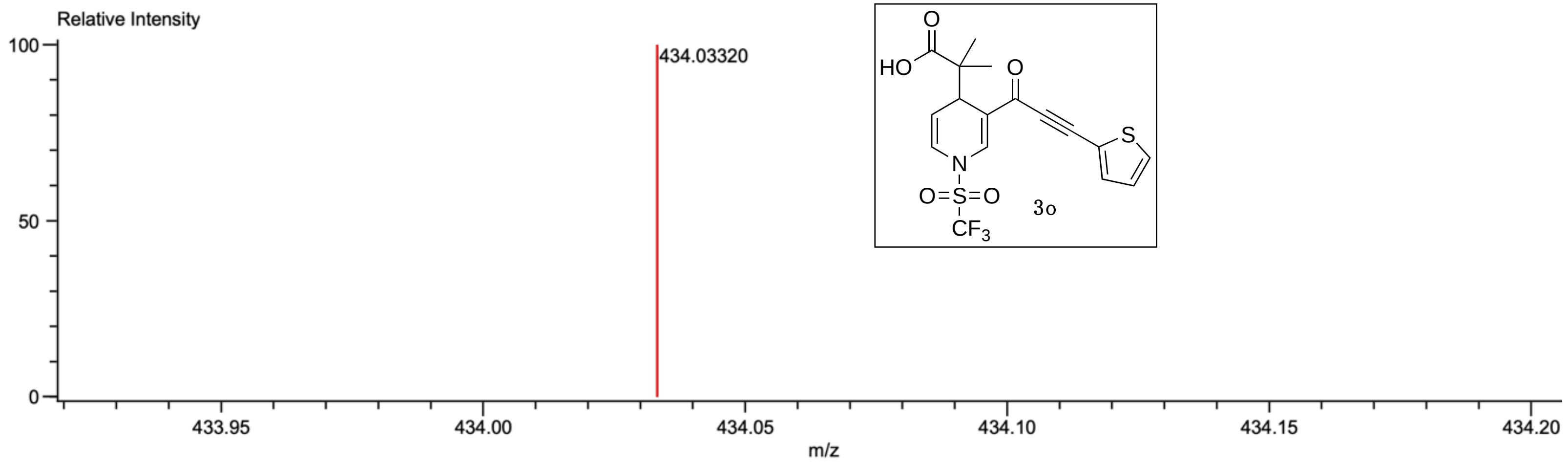

3o

Mass	Intensity	Calc. Mass	Mass Difference (mmu)	Mass Difference (ppm)	Possible Formula	Unsaturation Number
434.03320	1425761.75	434.03437	-1.18	-2.71	$^{12}C_{17}{}^{1}H_{15}{}^{19}F_{3}{}^{14}N_{1}{}^{16}O_{5}{}^{32}S_{2}$	11.5

Figure S60. HRMS-DART+ (19 eV) of **3o**.

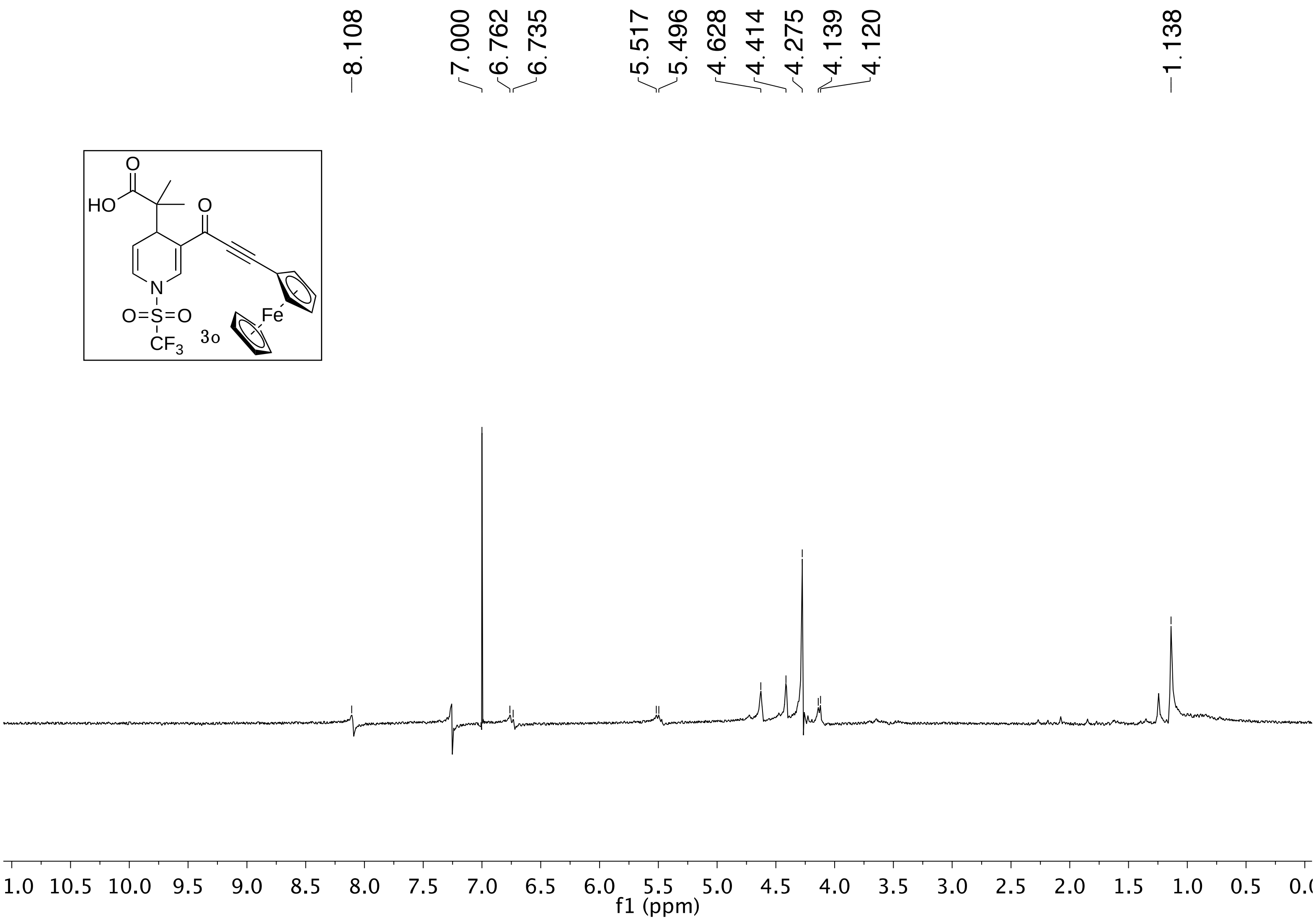

Figure S61. ^1H NMR (300 MHz/$CDCl_3$/TMS) of **3p**.

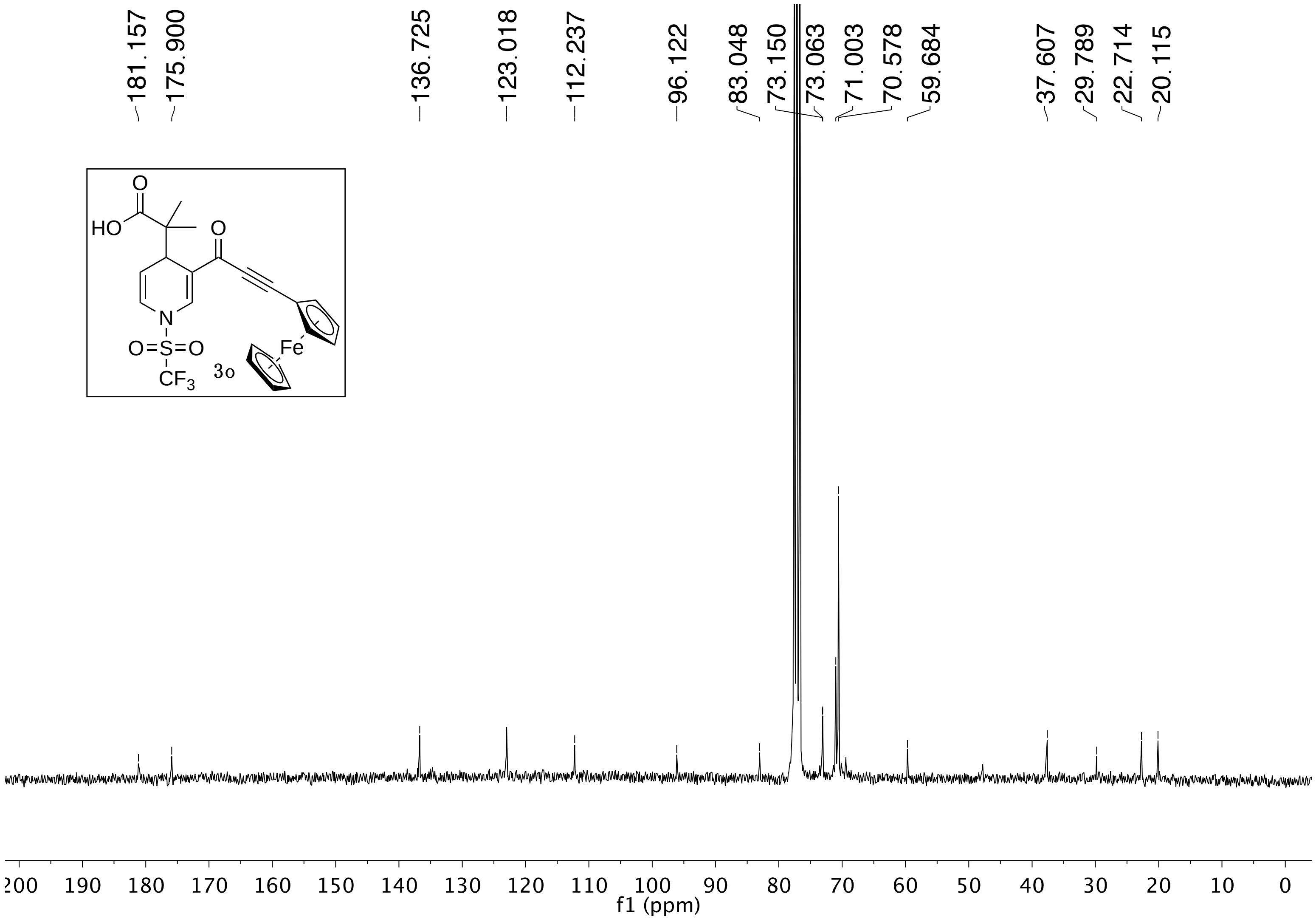

Figure S62. ^{13}C NMR (75 MHz/$CDCl_3$/TMS) of **3p**.

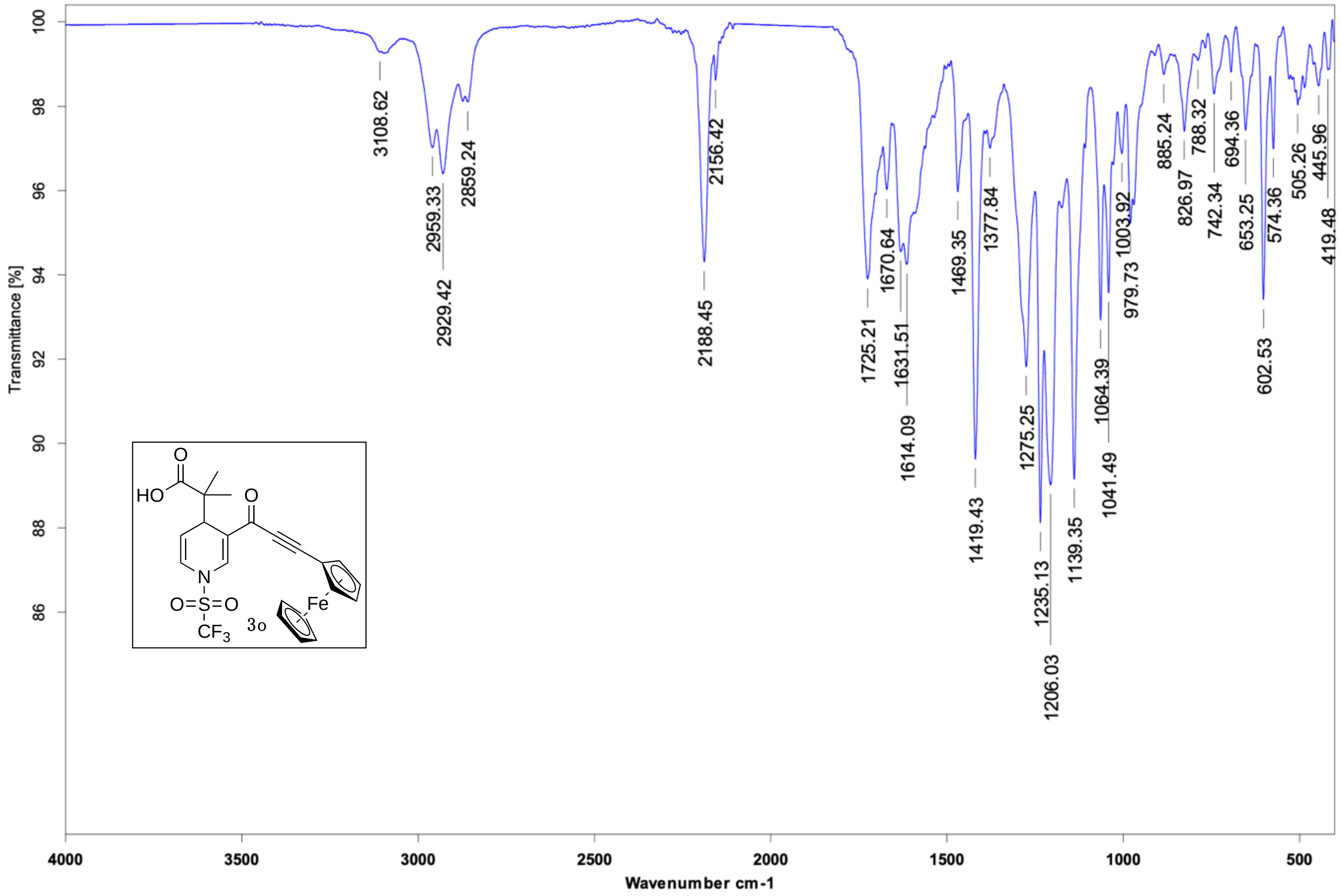

Figure S63. FTIR of **3p**.

Description:
Ionization Mode:ESI+
History:Determine m/z[Peak Detect[Centroid,30,Area];Correct Base[1.0%];Smooth[5]];Correct Base[5.0%];Average...

Mass Calibration data:Cal_Peg_600
Created:11/22/2018 2:00:41 PM
Created by:AccuTOF

Charge number:1
Tolerance:3.00(mmu)
Unsaturation Number:0.0 .. 50.0 (Fraction:Both)
Element:^{12}C:0 .. 23, ^{1}H:15 .. 21, ^{19}F:0 .. 3, ^{56}Fe:1 .. 1, ^{14}N:0 .. 1, ^{16}O:0 .. 5, ^{32}S:0 .. 1

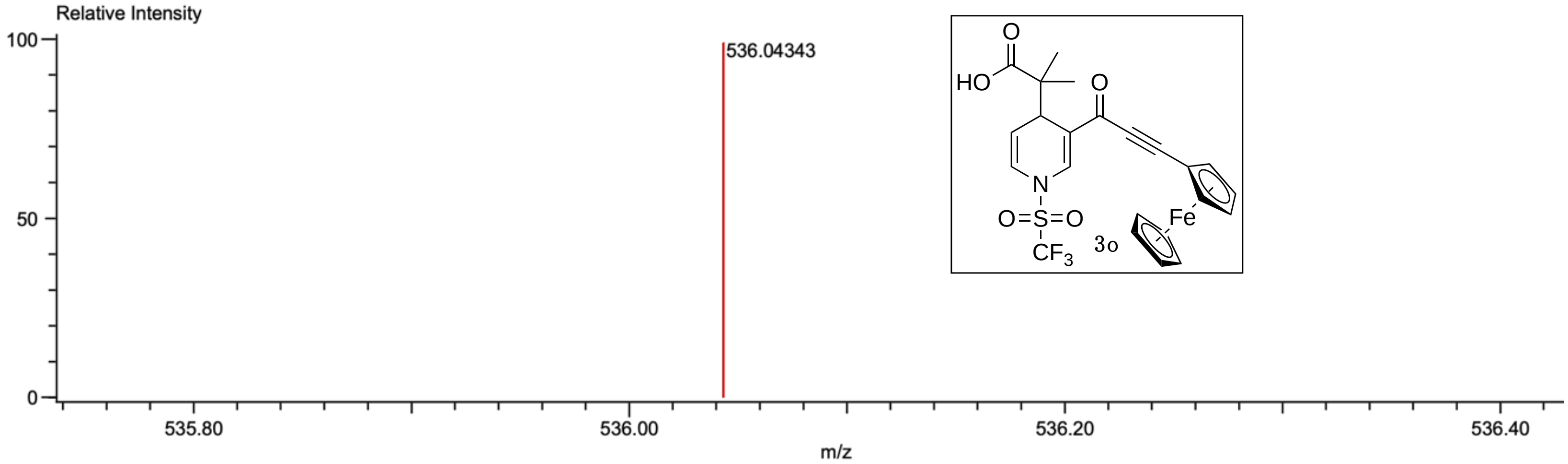

Mass	Intensity	Calc. Mass	Mass Difference (mmu)	Mass Difference (ppm)	Possible Formula	Unsaturation Number
536.04343	872634.69	536.04419	-0.77	-1.43	$^{12}C_{23}{}^{1}H_{21}{}^{19}F_{3}{}^{56}Fe_{1}{}^{14}N_{1}{}^{16}O_{5}{}^{32}S_{1}$	14.0

Figure S64. HRMS-DART+ (19 eV) of **3p**.
